# Approximation of Classical Two-Phase Flows of Viscous Incompressible Fluids by a Navier–Stokes/Allen–Cahn System

**DOI:** 10.1007/s00205-024-02020-9

**Published:** 2024-09-03

**Authors:** Helmut Abels, Julian Fischer, Maximilian Moser

**Affiliations:** 1 Fakultät für Mathematik, Universität Regensburg, 93040 Regensburg, Germany; 2 Institute of Science and Technology Austria, Am Campus 1, 3400 Klosterneuburg, Austria

**Keywords:** Primary: 76T06, Secondary: 35Q30, 35Q35, 35R35, 76D05, 76D45

## Abstract

We show convergence of the Navier–Stokes/Allen–Cahn system to a classical sharp interface model for the two-phase flow of two viscous incompressible fluids with same viscosities in a smooth bounded domain in two and three space dimensions as long as a smooth solution of the limit system exists. Moreover, we obtain error estimates with the aid of a relative entropy method. Our results hold provided that the mobility $m_\varepsilon >0$ in the Allen–Cahn equation tends to zero in a subcritical way, i.e., $m_\varepsilon = m_0 \varepsilon ^\beta$ for some $\beta \in (0,2)$ and $m_0>0$. The proof proceeds by showing via a relative entropy argument that the solution to the Navier–Stokes/Allen–Cahn system remains close to the solution of a perturbed version of the two-phase flow problem, augmented by an extra mean curvature flow term $m_\varepsilon H_{\Gamma _t}$ in the interface motion. In a second step, it is easy to see that the solution to the perturbed problem is close to the original two-phase flow.

## Introduction

During its evolution, the interface between two immiscible fluids may undergo topological changes, such as the merging or pinchoff of droplets. Mathematically, when modeling the interface classically as a $(d-1)$-dimensional manifold, this creates challenges for the analysis and for numerical approximations. Diffuse-interface models circumvent these problems by replacing the sharp interface by a diffuse transition layer of a finite width $\varepsilon >0$, reducing the problem to a set of PDEs posed on the entire domain. However, this procedure comes at the cost of introducing an additional modeling error: For many diffuse-interface models for fluid-fluid interfaces it has remained an open problem to rigorously show convergence to the original sharp-interface model in the limit of vanishing interface width $\varepsilon \rightarrow 0$, even prior to any topology change. In the present work, we prove the convergence of a diffuse-interface approximation for one of the most fundamental macroscopic models for a fluid-fluid interface, the Navier–Stokes equation for two immiscible incompressible fluids separated by a sharp interface with surface tension. To the best of our knowledge, the present work is the first general^1^ quantitative convergence result for any diffuse-interface approximation of the standard free boundary problem for the interface between two immiscible incompressible viscous fluids.

More specifically, in this contribution we rigorously identify the sharp-interface limit of the following Navier–Stokes/Allen–Cahn system

1.1a $$\begin{aligned} \partial _t {\textbf{v}}_\varepsilon +{\textbf{v}}_\varepsilon \cdot \nabla {\textbf{v}}_\varepsilon -\Delta {\textbf{v}}_\varepsilon +\nabla p_\varepsilon&= -\varepsilon {\text {div}}(\nabla \varphi _\varepsilon \otimes \nabla \varphi _\varepsilon )&\quad&\text {in}\ \Omega \times (0,T_0), \end{aligned}$$

1.1b $$\begin{aligned} {\text {div}}{\textbf{v}}_\varepsilon&= 0&\quad&\text {in}\ \Omega \times (0,T_0), \end{aligned}$$

1.1c $$\begin{aligned} \partial _t \varphi _\varepsilon +{\textbf{v}}_\varepsilon \cdot \nabla \varphi _\varepsilon&=m_\varepsilon \left( \Delta \varphi _\varepsilon - \tfrac{1}{\varepsilon ^{2}} W'(\varphi _\varepsilon )\right)&\quad&\text {in}\ \Omega \times (0,T_0), \end{aligned}$$

1.1d $$\begin{aligned} ({\textbf{v}}_\varepsilon ,\varphi _\varepsilon )|_{\partial \Omega }&= (0,-1)&\quad&\text {on }\partial \Omega \times (0,T_0), \end{aligned}$$

1.1e $$\begin{aligned} ({\textbf{v}}_\varepsilon ,\varphi _\varepsilon ) |_{t=0}&= ({\textbf{v}}_{0,\varepsilon },\varphi _{0,\varepsilon })&\quad&\text {in }\Omega , \end{aligned}$$

in a bounded domain $\Omega \subseteq {\mathbb {R}}^d$, $d=2,3$, with smooth boundary. Here ${\textbf{v}}_\varepsilon :\Omega \times (0,T_0)\rightarrow {\mathbb {R}}^d$ is the mean velocity of the fluid mixture, $p_\varepsilon :\Omega \times (0,T_0)\rightarrow {\mathbb {R}}$ its pressure and $\varphi _\varepsilon :\Omega \times (0,T_0)\rightarrow {\mathbb {R}}$ an order parameter (e.g. the volume fraction difference of the fluids) related to the different phases, where the values $\varphi _\varepsilon =\pm 1$ describe that only one fluid is present. Moreover, $\varepsilon >0$ is a constant related to the thickness of the diffuse interface and $m_\varepsilon >0$ is a constant diffusion coefficient, which depends on $\varepsilon >0$. Here $W:{\mathbb {R}}\rightarrow {\mathbb {R}}$ is a double well-potential, satisfying standard assumptions. More precisely, we assume that *W* is twice continuously differentiable and we have, for some $c>0$,

$$\begin{aligned} W(\pm 1) = 0, \quad W(-s)=W(s), \quad W(s)\ge c\min \{|s-1|^2, |s+1|^2\} \end{aligned}$$

for all $s\in {\mathbb {R}}$. A standard example is $W(s) = c(1-s^2)^2$ for $c>0$.

This model was introduced by Liu and Shen in [30] to describe a two-phase flow for incompressible fluids with the same viscosity and densities. For simplicity we have set the densities and viscosities to one. The model can be considered as the analogue of the well-know “model H”, cf. [21, 24], if one replaces the convective Cahn-Hilliard equation by a convective Allen–Cahn equation. A first analytic study of the system (1.1a)–(1.1e) was done by Gal and Grasselli [19] in the case of a bounded smooth domain in two space dimensions, where the existence of global and exponential attractors and convergence to stationary solutions was shown. For a more general model with different densities and viscosities Jiang et al. [25] proved the existence of weak solutions globally in time (in two and three space dimensions). Moreover, in the case of two space dimensions they proved global well-posedness in the strong sense and studied the longtime behavior of strong solutions. We refer to Giorgini et al. [20] for a mass-conserving variant of the Navier–Stokes/Allen–Cahn system with different densities and further references.

It is the purpose of this contribution to study the limit (1.1a)–(1.1e) as $\varepsilon \rightarrow 0$ in the case that $m_\varepsilon \rightarrow _{\varepsilon \rightarrow 0} 0$ suitably. Then one expects to have convergence to solutions of the classical two-phase Navier–Stokes equation with surface tension:

1.2a $$\begin{aligned} \partial _t {\textbf{v}}_0^\pm +{\textbf{v}}_0^\pm \cdot \nabla {\textbf{v}}_0^\pm -\Delta {\textbf{v}}_0^\pm +\nabla p^\pm _0&= 0&\qquad&\text {in }\Omega ^{\pm }_t, t\in [0,T_0], \end{aligned}$$

1.2b $$\begin{aligned} {\text {div}}{\textbf{v}}_0^\pm&= 0&\qquad&\text {in }\Omega ^{\pm }_t, t\in [0,T_0], \end{aligned}$$

1.2c $$\begin{aligned} \llbracket 2D{\textbf{v}}_0^\pm -p_0^\pm I \rrbracket {\textbf{n}}_{\Gamma _t}&= -\sigma H_{\Gamma _t}{\textbf{n}}_{\Gamma _t}  &   \text {on }\Gamma _t, t\in [0,T_0], \end{aligned}$$

1.2d $$\begin{aligned} \llbracket {\textbf{v}}_0^\pm \rrbracket&=0  &   \text {on }\Gamma _t, t\in [0,T_0], \end{aligned}$$

1.2e $$\begin{aligned} V_{\Gamma _t}&={\textbf{n}}_{\Gamma _t}\cdot {\textbf{v}}_0^\pm  &   \text {on }\Gamma _t, t\in [0,T_0], \end{aligned}$$

1.2f $$\begin{aligned} {\textbf{v}}_0^-|_{\partial \Omega }&= 0  &   \text {on }\partial \Omega \times (0,T_0), \end{aligned}$$

1.2g $$\begin{aligned} \Gamma _0=\Gamma ^0,\qquad {\textbf{v}}_0^\pm |_{t=0}&= {\textbf{v}}_{0,0}^\pm  &   \text { in }\Omega ^{\pm }_0. \end{aligned}$$

Here $(\Gamma _t)_{t\in [0,T_0]}$ is an evolving $(d-1)$-dimensional submanifold of $\Omega$ such that $\Omega$ is the disjoint union of two smooth domains $\Omega ^\pm _t$ and $\Gamma _t$ as well as $\partial \Omega _t^\pm =\Gamma _t$ for every $t\in [0,T_0]$. Here the absence of a boundary contact of $\Gamma _t$ is assumed for all $t\in [0,T_0]$. Moreover, ${\textbf{v}}_0^\pm (.,t):\Omega ^\pm _t\rightarrow {\mathbb {R}}^d$ and $p_0^\pm (.,t):\Omega ^\pm _t\rightarrow {\mathbb {R}}$ are the velocity and pressure of two fluids filling $\Omega ^+_t$ and $\Omega _t^-$ for every $t\in [0,T_0]$, ${\textbf{n}}_{\Gamma _t}$ denotes the interior normal of $\Gamma _t$ with respect to $\Omega ^+_t$, $H_{\Gamma _t}$ and $V_{\Gamma _t}$ denote the mean curvature (sum of principle curvature) and normal velocity, resp., of $\Gamma _t$ with respect to the orientation given by ${\textbf{n}}_{\Gamma _t}$. Furthermore, $\llbracket f \rrbracket (s)= \lim _{h\rightarrow 0+}(f(s+h{\textbf{n}}_{\Gamma _t}(s))-f(s+h{\textbf{n}}_{\Gamma _t}(s)))$, $s\in \Gamma _t$, denotes the jump of a function *f* defined in a neighborhood of $\Gamma _t$ and $D{\textbf{v}}_0^\pm :=\frac{1}{2}\left( \nabla {\textbf{v}}_0^\pm +(\nabla {\textbf{v}}_0^\pm )^\top \right)$ is the symmetric gradient. Finally, $\sigma = \int _{-1}^1\sqrt{2W(s)}\,ds>0$ is a surface tension coefficient. For what follows we set that

1.3 $$\begin{aligned} \Gamma :=\bigcup _{t\in [0,T_0]}\Gamma _t\times \{t\},\quad \Omega ^\pm :=\bigcup _{t\in [0,T_0]}\Omega _t^\pm \times \{t\}, \quad {\textbf{v}}^0:=\sum _\pm {\textbf{v}}_0^\pm \chi _{\Omega ^\pm }. \end{aligned}$$

For given ${\textbf{v}}_\varepsilon ={\textbf{v}}$ the convective Allen–Cahn equation, i.e., (1.1c)–(1.1d), was discussed formally by the first author in [2] and it was shown formally that the limit system is given by the transport Eqs. (1.2e)–(1.2f) in the case that $m_\varepsilon =m_0 \varepsilon$ for some $m_0>0$. We note that in this case these arguments can be extended to show formally convergence of the full system (1.1a)–(1.1e) to (1.2a)–(1.2f) by combining it with the arguments in [5] for a Navier–Stokes/Cahn-Hilliard system. But in the case that $m_\varepsilon =m_0 \varepsilon ^\beta$ for some $\beta >2$ nonconvergence was shown in the sense that, in general,

$$\begin{aligned} \varphi _\varepsilon (x,t) = \theta _0\left( \frac{d_{\Gamma _t}(x)}{\varepsilon }\right) + O(\varepsilon )\qquad \text {as }\varepsilon \rightarrow 0, \end{aligned}$$

where $d_{\Gamma _t}$ is the signed distance function to $\Gamma _t$, no longer holds and a weak formulation of the right-hand side of (1.1a) does not converge to the mean curvature functional $\sigma H_{\Gamma _t}{\textbf{n}}_{\Gamma _t}\delta _{\Gamma _t}$, which appears in a weak formulation of (1.2a) and (1.2c). Here $\theta _0:{\mathbb {R}}\rightarrow {\mathbb {R}}$ is the so-called optimal profile, which is the unique solution of

$$\begin{aligned} -\theta _0''(s)+ W'(\theta _0(s))= 0\quad \text {for all }s\in {\mathbb {R}},\quad \theta _0(s)\rightarrow _{s\rightarrow \pm \infty } \pm 1,\quad \theta _0(0)=0. \end{aligned}$$

Therefore convergence of the full system (1.1a)–(1.1e) cannot be expected in this case. We note that in this case the counterexample given in [6] for a Navier–Stokes/Cahn-Hilliard system in a radially symmetric situation can be adapted to the present Navier–Stokes/Allen–Cahn system. It is the purpose of the present contribution to show convergence of solutions of (1.1a)–(1.1e) to the smooth solution of (1.2a)–(1.2f) on a time interval $[0,T_0]$ for which the latter exists in the case of a subcritical scaling of the mobility $m_\varepsilon = m_0\varepsilon ^\beta$ for some $\beta \in (0,2)$. Moreover, we will derive error estimates with the aid of a relative entropy method. We note that this is the first rigorous convergence result for a vanishing mobility $m_\varepsilon \rightarrow _{\varepsilon \rightarrow 0}0$ in this regime, which includes the natural choice $m_\varepsilon =m_0\varepsilon$. Finally, let us remark that the derivation of a similar convergence result using a relative entropy method was attempted in the recent work [26]; however, as the approach of [26] relies on the invalid estimate [26, equation (2.4)], it overlooks the need to devise a careful estimate for the critical interface stretching term that forms the main challenge for our result.

Except for [4], so far only convergence in the case of a non-vanishing mobility $m_\varepsilon = m_0>0$ for all $\varepsilon >0$ has been shown. First this was done in the case of a Stokes/Allen–Cahn system with same viscosities by Abels and Liu [7], then for a Navier–Stokes/Allen–Cahn system with different viscosities in Abels and Fei [3], both in two space dimensions, and by Hensel and Liu [22] for the Navier–Stokes/Allen–Cahn system with same viscosities in two and three space dimensions. We note that the first two results are based on a refined spectral estimate for the linearized Allen–Cahn operator and rigorous asymptotic expansions, while the latter result uses the relative entropy method similarly as for the convergence of the Allen–Cahn equation to the mean curvature flow shown by Fischer, Laux, and Simon [17]. In the case of a non-vanishing mobility the limit system consists of a system, where (1.2e) is replaced by a convective mean curvature flow equation

$$\begin{aligned} V_{\Gamma _t} = {\textbf{n}}_{\Gamma _t}\cdot {\textbf{v}}_0^\pm + m_0 H_{\Gamma _t}\qquad \text {on }\Gamma _t,t\in [0,T_0]. \end{aligned}$$

In the contribution by Abels, Fei, and Moser [4] convergence of a Navier–Stokes/Allen–Cahn system with different viscosities was shown in the special case of $m_\varepsilon = m_0 \sqrt{\varepsilon }$ and two space dimensions using the same method as in [3, 7] refined for this degenerate case. We note that the arguments could be extended to $m_\varepsilon =m_0\varepsilon ^{\beta }$ for $\beta \in (0,\tfrac{1}{2}]$, but the case $\beta =\frac{1}{2}$ seems to be critical for the estimates in this contribution and new ideas and refinements seem to be needed to treat the cases with $\beta >\frac{1}{2}$ with this method. At this stage, the relative entropy method used in the present contribution appears to be more flexible.

In the following we consider a situation, in which the limit system (1.2a)–(1.2g) is known to possess a unique smooth solution for some $T_0>0$. We note that strong well-posedness of this system was extensively studied starting with the results by Denisova and Solonnikov [14]. Moreover, it was shown by Prüss and Simonett [34] that strong solutions become analytic instantaneously in time. We refer to Köhne, Prüss, and Wilke [27] and the monograph by Prüss and Simonett [35] for results on local well-posedness in an $L^p$-setting and further references. Finally, we note that global-in-time existence of a notion of weak solutions, called varifold solutions, was shown in [1] and weak-strong uniqueness for these kind of solutions was shown by Fischer and Hensel [16].

In general, there are two main mathematical approaches to the quantitative justification of sharp-interface limits: an approach pioneered by de Mottoni and Schatzman [13] and Chen [12] relies on a matched asymptotic expansion around the sharp-interface limit to obtain an approximate solution to the diffuse interface model; by means of a stability analysis of the linearized operator, it is possible to derive rates of convergence. This approach has recently also been successfully adapted to our Navier–Stokes/Allen–Cahn system with mobility $m_\varepsilon =\varepsilon ^{1/2}$ by Fei and the first and the third author [4]. An alternative approach – recently developed by the second author, Laux, and Simon [17] – proceeds via a suitably defined relative entropy. In [17], the relative entropy approach is used to give a short proof of convergence of the Allen–Cahn equation towards mean curvature flow (valid for well-prepared initial data and as long as a classical solution to the latter exists); this result was extended in [23] to interfaces with boundary contact and in [29] to the anisotropic case. The general approach has found numerous further applications: In [18], convergence of the vectorial Allen–Cahn equation with multi-well potential towards multiphase mean curvature flow has been established by the second author and Marveggio; the case of the vectorial Allen–Cahn equation with two-well potential has been considered by Liu [31, 32]. Furthermore, Laux and Liu [28] have obtained the sharp-interface limit for a model for liquid crystals. Hensel and Liu [22] have used the relative entropy approach to study the sharp-interface limit of the Navier–Stokes/Allen–Cahn system (1.1a)–(1.1e) in the regime of nonvanishing mobility, deriving a Navier–Stokes/mean curvature flow system in the limit. In general, a key advantage of the relative entropy approach to sharp interface limits is its robustness, for instance requiring only convergence of the initial energy of solutions to the phase-field model. In contrast, the approach of matched asymptotic expansions may be used to establish an approximation of the diffuse interface model to arbitrary order.

The main result of our contribution is as follows:

### Theorem 1.1

(Convergence) Let $m_\varepsilon :=m_0\varepsilon ^\beta$ for $\varepsilon >0$, where $m_0>0$ and $\beta \in (0,2)$ are fixed, $q=2$ if $d=2$, and $q=\frac{4}{3}$ if $d=3$. Moreover, let $T_0>0$ be such that the two-phase Navier–Stokes system with surface tension (1.2a)–(1.2g) has a smooth solution $({\textbf{v}}_0^\pm ,p_0^\pm ,\Gamma )$ on $[0,T_0]$. Let $({\textbf{v}}_\varepsilon , \varphi _\varepsilon )$ with ${\textbf{v}}_\varepsilon \in L^\infty (0,T_0;L^2_\sigma (\Omega ))\cap L^2(0,T_0;H^1_0(\Omega )^d)$, $\varphi _\varepsilon \in L^2(0,T_0;H^2(\Omega ))\cap W^1_{q}(0,T_0;L^2(\Omega ))$ for $\varepsilon >0$ be energy-dissipating weak solutions to the Navier–Stokes/Allen–Cahn system (1.1a)–(1.1e) on $[0,T_0]$ as in Remark 1.2 below for the constant mobility $m_\varepsilon$ and for initial data $({\textbf{v}}_{0,\varepsilon },\varphi _{0,\varepsilon })$ with energy uniformly bounded with respect to $\varepsilon$ and satisfying

1.4 $$\begin{aligned} \begin{aligned}&\sum _{\pm }\int _{\Omega _0^\pm } \frac{1}{2}|{\textbf{v}}_{0,\varepsilon }-{\textbf{v}}^\pm _{0,0}|^2\,dx + \int _\Omega \frac{\varepsilon }{2}|\nabla \varphi _{0,\varepsilon }|^2+ \frac{1}{\varepsilon } W(\varphi _{0,\varepsilon })-(\xi \cdot \nabla \psi _{0,\varepsilon })\,dx\\&\quad + \int _\Omega |\sigma \chi _{\Omega ^+_0} -\psi _{0,\varepsilon }|\min \{{\text {dist}}_{\Gamma ^0},1\}\,dx\le C\frac{\varepsilon ^2}{m_\varepsilon }\end{aligned} \end{aligned}$$

for $\varepsilon >0$ sufficiently small, where $\psi (r):=\int _{-1}^r\sqrt{2W(s)}\,ds$ and $\psi _{0,\varepsilon }:=\psi (\varphi _{0,\varepsilon })$. Set $\psi _\varepsilon :=\psi (\varphi _\varepsilon )$.

Then, for $\varepsilon >0$ small and a.e. $T\in [0,T_0]$, it holds that

1.5a $$\begin{aligned} \Vert ({\textbf{v}}_\varepsilon -{\textbf{v}}^0)(.,T)\Vert _{L^2(\Omega )} + \Vert \sigma \chi _{\Omega ^+_T} -\psi _\varepsilon (.,T)\Vert _{L^1(\Omega )}&\le C\left( \frac{\varepsilon }{\sqrt{m_\varepsilon }}+m_\varepsilon \right) \end{aligned}$$

1.5b $$\begin{aligned} \Vert \nabla {\textbf{v}}_\varepsilon -\nabla {\textbf{v}}^0\Vert _{L^2(0,T_0;L^2(\Omega ))}&\le C\left( \frac{\varepsilon }{\sqrt{m_\varepsilon }}+m_\varepsilon \right) \end{aligned}$$

for some $C>0$ independent of $\varepsilon >0$ and $T\in [0,T_0]$. Finally, there are well-prepared initial data $({\textbf{v}}_{0,\varepsilon },\varphi _{0,\varepsilon })$ for $\varepsilon >0$ small in the sense that (1.4) is satisfied, even with rate $\varepsilon ^2$.

This result will be a consequence of Corollary 3.2 below.

Let us briefly comment on some aspects of our main theorem. First, note that the choice $m_\varepsilon :=\varepsilon ^{2/3}$ leads to the best-possible overall convergence rate $O(\varepsilon ^{2/3})$ in (1.5). If one employs the Navier–Stokes/Allen–Cahn system as a numerical approximation of the two-phase flow with sharp interface, this thus suggests the choice of mobility $m_\varepsilon \sim \varepsilon ^{2/3}$.

Second, our theorem requires a lower bound on the mobility of the form $m_\varepsilon \gtrsim \varepsilon ^\beta$ for some $\beta <2$. With a slightly more careful argument, it would in fact be possible to weaken this assumption to $m_\varepsilon \gtrsim \varepsilon ^2 |\log \varepsilon |^3$. However, in the regime $m_\varepsilon \lesssim \varepsilon ^2$ one would expect that the Allen–Cahn term no longer suffices to stabilize the Modica-Mortola type profile of the diffuse interface, making it susceptible to stretching or squeezing by drift: To see this heuristically, notice for instance that in this scaling regime the reaction term $-\tfrac{m_\varepsilon }{\varepsilon ^2} W'(\varphi _\varepsilon )$ from the Allen–Cahn Eq. (1.1c) no longer suffices to drive the values of $\varphi _\varepsilon$ towards the minima of the potential *W* in finite time. This possible stretching of the profile by advection in turn is expected to lead to errorneous capillary effects in (1.1a) and hence convergence to a different limiting system; see also [6], in which this has been observed rigorously for certain choices $m_\varepsilon =\varepsilon ^\beta$, $\beta >2$.

### Remark 1.2

Let $q=2$ if $d=2$, and $q=\frac{4}{3}$ if $d=3$. We note that weak solutions $({\textbf{v}}_\varepsilon , \varphi _\varepsilon )$ with ${\textbf{v}}_\varepsilon \in L^\infty (0,T_0;L^2_\sigma (\Omega ))\cap L^2(0,T_0;H^1_0(\Omega )^d)$, $\varphi _\varepsilon \in L^2(0,T_0;H^2(\Omega ))\cap W^1_{q}(0,T_0;L^2(\Omega ))$ for $\varepsilon >0$ to the Navier–Stokes/Allen–Cahn system (1.1a)–(1.1e) on $[0,T_0]$ are understood in the sense of [22, Definition 3] with the difference that we only require $\varphi _\varepsilon \in W^1_{4/3}(0,T_0;L^2(\Omega ))$ if $d=3$. In particular, we assume that the energy inequality

$$\begin{aligned}&\int _\Omega \left( \frac{|{\textbf{v}}_\varepsilon (x,t)|^2}{2}+\frac{\varepsilon }{2}|\nabla \varphi _\varepsilon (x,t)|^2+ \frac{W(\varphi _\varepsilon (x,t))}{\varepsilon }\right) \,dx \\&\qquad +\int _0^t \int _\Omega \left( |\nabla {\textbf{v}}_\varepsilon (x,\tau )|^2+ |\partial _t \varphi _\varepsilon (x,\tau )+{\textbf{v}}_\varepsilon (x,\tau )\cdot \nabla \varphi _\varepsilon (x,\tau )|^2\right) \,dx \,d \tau \\&\quad \le \int _\Omega \left( \frac{|{\textbf{v}}_{0,\varepsilon }|^2}{2}+\frac{\varepsilon }{2}|\nabla \varphi _{0,\varepsilon }|^2+ \frac{W(\varphi _{0,\varepsilon })}{\varepsilon }\right) \,dx\quad \text {for every } t\in [0,T_0] \end{aligned}$$

holds true, which is essential for the following arguments. Existence of weak solutions follows from the results in [25]. We note that in the latter contribution the authors do not prove the energy inequality. However, it can be proved in a standard manner using the energy identity for the solutions of the Galerkin system in [25] and lower semi-continuity of norms. We also refer to [20, Theorem 3.1] for the mass-conserved variant of the system.

Let us comment on the novelty of our contribution. As mentioned before, this is the first convergence result for (1.1a)–(1.1e) in the case of a vanishing mobility $m_\varepsilon \rightarrow 0$, except for the special case $m_\varepsilon =m_0 \sqrt{\varepsilon }$ in two space dimensions. We use a relative entropy similar as in [22], which extends the one for the Allen–Cahn equation used in [17] to the coupled Navier–Stokes/Allen–Cahn system. The main step in the proof of convergence consists in showing a suitable estimate for the relative entropy. Parts of the arguments and calculations in our situation follow closely [22], but certain estimates degenerate as $m_\varepsilon \rightarrow 0$ and some terms become critical. Therefore essential new ingredients are needed.

More precisely, it will turn out that the main challenge in controlling the growth of the relative entropy for $m_\varepsilon \ll 1$ consist of controlling terms involving the failure of equipartition of energy such as

$$\begin{aligned} \int \left( \frac{\varepsilon }{2}|\partial _n\varphi _\varepsilon |^2-\frac{1}{\varepsilon }W(\varphi _\varepsilon )\right) \partial _n{\tilde{\eta }}\,dx. \end{aligned}$$

A naive direct estimate would bound such terms by the square root of the relative entropy, insufficient for a subsequent control of the growth via the Gronwall lemma. Instead, we shall see that by careful integration by parts arguments and an approximation of the diffuse interface by a graph, they may in fact be controlled by the dissipation term from the Allen–Cahn equation and the relative entropy, provided that $m_\varepsilon \gg \varepsilon ^2$.

We deal with these critical terms in Sects. 4.2–4.4. The estimates make use of a suitable parametrization of a suitably chosen level set $\{\varphi _\varepsilon = b(t)\}$ for some $b(t)\in (-\tfrac{1}{2},\tfrac{1}{2})$ up to an error controlled by the relative entropy using results from [16]. Moreover, it is essential for the construction of the relative entropy that we use sufficiently smooth solutions of a modification of the limit system (1.2a)–(1.2g), where the interface evolution (1.2e) is replaced by the convective mean curvature flow equation with vanishing mobility

$$\begin{aligned} V_{\Gamma _t} = {\textbf{n}}_{\Gamma _t}\cdot {\textbf{v}}_0^\pm + m_\varepsilon H_{\Gamma _t}\qquad \text {on }\Gamma _t,t\in [0,T_0]. \end{aligned}$$

This is used to obtain a sufficiently good approximation and control of some critical remainder terms. However, it makes the solution depend on $m_\varepsilon$, $\varepsilon$, respectively, and existence of such solutions together with uniform estimates in $\varepsilon >0$ sufficiently small needs to be shown. We note that existence of strong solutions for a fixed $m_\varepsilon >0$ locally in time was shown by the first and third author in [8] and in [22, Appendix]. But the existence time might depend on $\varepsilon$. To obtain the uniform bounds for small $\varepsilon >0$ a Hanzawa transformation is used to transform the modified system of the limit system (1.2a)–(1.2g). Then a fixed point argument can be used to obtain strong solutions for $m_\varepsilon$ sufficiently small using suitable uniform bounds for the $m_\varepsilon$-dependent linearized system in spaces of maximal $L^q$-regularity.

The structure of the contribution is as follows: in Sect. 2 we define the energy-type functionals, which will be essential for the proof of convergence, and study their coercivity properties. Afterwards the central stability estimate and convergence result is given in Sect. 3. The essential estimates for the relative entropy are done in Sect. 4. The proof of the stability estimate, which implies the convergence result, is given in Sect. 6. Finally, in Sect. 7 existence of strong solutions for the modification of the limit system (1.2a)–(1.2g) for sufficiently small $m_\varepsilon >0$ is shown and its difference to the solution of the real limit system is estimated by a multiple of $m_\varepsilon$, cf. Theorem 7.7 below.

Let us finish the introduction with some notation. For example, we denote with $W^k_p(\Omega )$ for $k\in {\mathbb {N}}$, $1\le p\le \infty$ and some domain $\Omega$ the Sobolev space with *k* weak derivatives and integrability exponent *p*. Moreover, let $H^k:=W^k_2$ and $L^2_\sigma (\Omega ):=\overline{\{{\textbf{v}}\in C_0^\infty (\Omega )^d:div \,{\textbf{v}}=0\}}^{L^2(\Omega )^d}$, where $C_0^\infty (\Omega )$ is the space of smooth functions with compact support in $\Omega$.

## Definition and Coercivity Properties of the Energy Functionals

In this section we define the energy/entropy functionals used for our Gronwall argument and show suitable coercivity properties. The definitions are similar to [22], but based on a modification of the limit system as mentioned in the introduction. To this end, we need some notation.

Let $({\textbf{v}}_\varepsilon , \varphi _\varepsilon )$ with ${\textbf{v}}_\varepsilon \in L^\infty (0,T_0;L^2_\sigma (\Omega ))\cap L^2(0,T_0;H^1_0(\Omega )^d)$ and $\varphi _\varepsilon \in L^2(0,T_0;H^2(\Omega ))\cap W^1_{q}(0,T_0;L^2(\Omega ))$, where $q=2$ if $d=2$ and $q=\frac{4}{3}$ if $d=3$, for $\varepsilon >0$ small be energy-dissipating weak solutions to the Navier–Stokes/Allen–Cahn system (1.1a)–(1.1e) on $[0,T_0]$ with constant mobility $m_\varepsilon >0$ as in Remark 1.2. Moreover, for $m_\varepsilon >0$ small let $({\textbf{v}}_{m_\varepsilon }^\pm ,p_{m_\varepsilon }^\pm ,(\Gamma _t^{m_\varepsilon })_{t\in [0,T_0]})$ be solutions of the two-phase Navier–Stokes equation with surface tension on $[0,T_0]$ but with the evolution equation $V_{\Gamma ^{m_\varepsilon }_t}={\textbf{n}}_{\Gamma ^{m_\varepsilon }_t}\cdot {\textbf{v}}_{m_\varepsilon }^\pm +m_\varepsilon H_{\Gamma ^{m_\varepsilon }_t}$ on $\Gamma _t^{m_\varepsilon }$ instead of (1.2e), cf. (7.1)–(7.7) and Theorem 7.9 in Sect. 7 below. We define that

2.1 $$\begin{aligned} \Gamma ^{m_\varepsilon }&:=\bigcup _{t\in [0,T_0]}\Gamma ^{m_\varepsilon }_t\times \{t\},\nonumber \\ \Omega ^{m_\varepsilon ,\pm }&:=\bigcup _{t\in [0,T_0]}\Omega _t^{m_\varepsilon ,\pm }\times \{t\}\quad \text{ and } \quad {{\textbf {v}}}^{m_\varepsilon }:=\sum _\pm {{\textbf {v}}}_{m_\varepsilon }^\pm \chi _{\Omega ^{m_\varepsilon ,\pm }}. \end{aligned}$$

Let $d_{\Gamma ^{m_\varepsilon }}$ be the signed distance function of $\Gamma ^{m_\varepsilon }$ and $P_{\Gamma ^{m_\varepsilon }}$ be the orthogonal projection on a tubular neighbourhood $\Gamma ^{m_\varepsilon }(2\delta )$ of $\Gamma ^{m_\varepsilon }$ of the width $2\delta$, where $\delta >0$ is sufficiently small and can be chosen independently of $m_\varepsilon >0$ sufficiently small, cf. Remark 7.8. Let $\Gamma ^{m_\varepsilon }_t(2\delta ):=\Gamma ^{m_\varepsilon }(2\delta )\cap ({\mathbb {R}}^d\times \{t\})$ be the time-slice. On $\Gamma ^{m_\varepsilon }(2\delta )$ we set that

2.2 $$\begin{aligned} {\textbf {n}} :={\textbf {n}} _{\Gamma ^{m_\varepsilon }}|_{P_{\Gamma ^{m_\varepsilon }}}\quad \text { and }\quad H:=H_{\Gamma ^{m_\varepsilon }}|_{P_{\Gamma ^{m_\varepsilon }}}, \end{aligned}$$

where we avoided to add $m_\varepsilon$ in the notation for ${\textbf {n}}$ and *H* for convenience, and we used the notation $f|_{P_{\Gamma ^{m_\varepsilon }}}:=f\circ P_{\Gamma ^{m_\varepsilon }}$ for suitable *f*. Moreover, we denote by $X_{m_\varepsilon }:=(d_{\Gamma ^{m_\varepsilon }},P_{\Gamma ^{m_\varepsilon }},pr _t)^{-1}$ on $(-2\delta ,2\delta )\times \Gamma ^{m_\varepsilon }$ the coordinate map describing the tubular neighbourhood strip $\Gamma ^{m_\varepsilon }(2\delta )$ and define the normal derivative and the tangential gradient by

2.3 $$\begin{aligned} \partial _n:={\textbf {n}} \cdot \nabla \quad \text { and }\quad \nabla _\tau :=(Id -{\textbf {n}} \otimes {\textbf {n}} )\nabla ,\quad \text {respectively}. \end{aligned}$$

On $\Gamma ^{m_\varepsilon }(2\delta )$ it holds that

2.4 $$\begin{aligned} \nabla&={\textbf {n}} \partial _n+\nabla _\tau ,\qquad |\nabla u|^2=|\partial _n u|^2+|\nabla _\tau u|^2\quad \text { for suitable }u, \end{aligned}$$

2.5 $$\begin{aligned} \partial _n&=[\partial _1(.\circ {X_{m_\varepsilon }})]\circ {X_{m_\varepsilon }^{-1}}\quad \text { and }\quad \nabla _\tau =D_xP_{\Gamma ^{m_\varepsilon }}\,[\nabla _{\Gamma ^{m_\varepsilon }_t}(.\circ {X_{m_\varepsilon }})]\circ {X_{m_\varepsilon }^{-1}}. \end{aligned}$$

Finally, let us recall that

2.6 $$\begin{aligned} \psi (r):=\int _{-1}^r \sqrt{2W(s)}\,ds,\quad \sigma :=\psi (1)\quad \text { and }\quad \psi _\varepsilon :=\psi (\varphi _\varepsilon ) \end{aligned}$$

and define that

2.7 $$\begin{aligned} {\textbf {n}} _\varepsilon :={\left\{ \begin{array}{ll} \frac{\nabla \varphi _\varepsilon }{|\nabla \varphi _\varepsilon |},\quad & \text { if }\nabla \varphi _\varepsilon \ne 0,\\ {\textbf {s}} ,\quad & \text { else}, \end{array}\right. } \end{aligned}$$

where ${\textbf {s}}$ is a fixed unit vector in ${\mathbb {R}}^d$. Then $\psi _\varepsilon$ and ${\textbf {n}} _\varepsilon$ are defined on $\Omega \times (0,T_0)$ and it holds that

$$\begin{aligned} {\textbf {n}} _\varepsilon |\nabla \varphi _\varepsilon |=\nabla \varphi _\varepsilon \quad \text { and }\quad {\textbf {n}} _\varepsilon |\nabla \psi _\varepsilon |=\nabla \psi _\varepsilon . \end{aligned}$$

### Relative Entropy

We define the relative entropy as follows for a.e. $t\in [0,T_0]$:

2.8 $$\begin{aligned} E[{\textbf{v}}_\varepsilon ,\varphi _\varepsilon | {\textbf{v}}^{m_\varepsilon },\Gamma ^{m_\varepsilon }](t)&:=\int _\Omega \frac{1}{2}|{\textbf{v}}_\varepsilon -{\textbf{v}}^{m_\varepsilon }|^2(x,t)\,\textrm{d}x + E[\varphi _\varepsilon |\Gamma ^{m_\varepsilon }](t), \end{aligned}$$

2.9 $$\begin{aligned} E[\varphi _\varepsilon |\Gamma ^{m_\varepsilon }](t)&:= \int _\Omega \frac{\varepsilon }{2}|\nabla \varphi _\varepsilon |^2(x,t) + \frac{1}{\varepsilon } W(\varphi _\varepsilon (x,t))-(\xi \cdot \nabla \psi _\varepsilon )(x,t)\,\textrm{d}x. \end{aligned}$$

Here we chose to introduce a separate notation for the second interface-related part for convenience. Here $\xi$ is an extension (with quadratic cutoff) of the unit normal on $\Gamma ^{m_\varepsilon }$. Note that the interface-related part of the relative entropy – being the same as the relative entropy in [17] – is motivated by the Modica-Mortola trick; as we shall see below, it controls both the error in the equipartition of the diffuse interface energy (2.14) and a tilt-excess type error quantity for the interface normal (2.13). At the same time, the time evolution of the relative entropy (2.9) can be calculated in a straightforward manner using the energy dissipation inequality for the Navier–Stokes/Allen–Cahn equation as well as the phase-field Eq. (1.1c).

For the precise definition of $\xi :{\overline{\Omega }}\times [0,T_0]\rightarrow {\mathbb {R}}^d$ and an accompanying $B:{\overline{\Omega }}\times [0,T_0]\rightarrow {\mathbb {R}}^d$ (which has the role of an approximate transport and rotational velocity for $\xi$) we first introduce suitable cutoff functions (analogous to [22, Proof of Theorem 1]). Let ${\bar{\eta }}:{\mathbb {R}}\rightarrow [0,1]$ be smooth and even such that ${\text {supp}}{\bar{\eta }}\subseteq [-1,1]$ and such that it satisfies the quadratic decay

2.10 $$\begin{aligned} 1-C_{{\bar{\eta }}} r^2 \le {\bar{\eta }}(r) \le 1-c_{{\bar{\eta }}} r^2 \quad \text { and }\quad |{\bar{\eta }}'(r)|\le C|r|\quad \text { for }r\in [-1,1], \end{aligned}$$

where $c_{{\bar{\eta }}}, C_{{\bar{\eta }}},C>0$. Moreover, let ${\tilde{\eta }}:{\mathbb {R}}\rightarrow [0,1]$ be smooth with ${\text {supp}}{\tilde{\eta }}\subseteq [-2,2]$ and ${\tilde{\eta }}=1$ on $[-1,1]$. Finally, we set $\eta _{m_\varepsilon }:={\bar{\eta }}(\frac{d_{\Gamma ^{m_\varepsilon }}}{\delta })$ and ${\tilde{\eta }}_{m_\varepsilon }:={\tilde{\eta }}(\frac{d_{\Gamma ^{m_\varepsilon }}}{\delta })$. Then we define

2.11 $$\begin{aligned} \xi := {\textbf {n}} \eta _{m_\varepsilon }\quad \text { and }\quad B:={\textbf{v}}^{m_\varepsilon }+m_\varepsilon H{\textbf {n}} {\tilde{\eta }}_{m_\varepsilon }, \end{aligned}$$

where ${\textbf {n}} ={\textbf {n}} (m_\varepsilon )$ and $H=H(m_\varepsilon )$ were defined in (2.2). For important properties of $\xi$ and *B* we refer to Lemma 2.2 below.

The relative entropy from (2.8) satisfies the following coercivity properties:

#### Lemma 2.1

(Coercivity Properties of the Relative Entropy) First, the relative entropy provides a control of the velocity error in terms of

2.12 $$\begin{aligned} \int _\Omega \frac{1}{2}|{\textbf{v}}_\varepsilon -{\textbf{v}}^{m_\varepsilon }|^2\,\textrm{d}x\le E[{\textbf{v}}_\varepsilon ,\varphi _\varepsilon | {\textbf{v}}^{m_\varepsilon },\Gamma ^{m_\varepsilon }]. \end{aligned}$$

Moreover, it yields a tilt-excess-type error estimate of the form

2.13 $$\begin{aligned} \int _\Omega (1-{\textbf {n}} _\varepsilon \cdot \xi )|\nabla \psi _\varepsilon |\,\textrm{d}x\le E[\varphi _\varepsilon |\Gamma ^{m_\varepsilon }]. \end{aligned}$$

Additionally, we have some control of the error in the equipartition of the energy in the sense

2.14 $$\begin{aligned} \int _\Omega \frac{1}{2}\left( \sqrt{\varepsilon }|\nabla \varphi _\varepsilon |-\frac{1}{\sqrt{\varepsilon }}\sqrt{2W(\varphi _\varepsilon )}\right) ^2\,\textrm{d}x \le E[\varphi _\varepsilon |\Gamma ^{m_\varepsilon }]. \end{aligned}$$

Furthermore, one obtains control of tangential derivatives and for the lack of equipartition of energy in normal direction: for a.e. $t\in [0,T_0]$ it holds that

2.15 $$\begin{aligned}&\int _{\Gamma ^{m_\varepsilon }_t(2\delta )} \frac{\varepsilon }{2}|\nabla _\tau \varphi _\varepsilon |^2(x,t) + \frac{1}{2}\left( \sqrt{\varepsilon }\partial _n\varphi _\varepsilon -\frac{1}{\sqrt{\varepsilon }}\sqrt{2W(\varphi _\varepsilon )}\right) ^2(x,t)\,\textrm{d}x\nonumber \\&\quad \le E[\varphi _\varepsilon |\Gamma ^{m_\varepsilon }](t), \end{aligned}$$

and one can replace $\partial _n\varphi _\varepsilon$ by $|\partial _n\varphi _\varepsilon |$ in the estimate. Finally, for some $C=C(\delta )>0$ it holds that

2.16 $$\begin{aligned} \int _\Omega \left( |{\textbf {n}} _\varepsilon -\xi |^2+\min \{d_{\Gamma ^{m_\varepsilon }}^2,1\}\right) \left( \varepsilon |\nabla \varphi _\varepsilon |^2+|\nabla \psi _\varepsilon |\right) \,\textrm{d}x&\le C E[\varphi _\varepsilon |\Gamma ^{m_\varepsilon }], \end{aligned}$$

2.17 $$\begin{aligned} \int _\Omega \left( \min \{d_{\Gamma ^{m_\varepsilon }},1\}+\sqrt{1-{\textbf {n}} _\varepsilon \cdot \xi }\right) \left| \varepsilon |\nabla \varphi _\varepsilon |^2-|\nabla \psi _\varepsilon |\right| \,\textrm{d}x&\le C E[\varphi _\varepsilon |\Gamma ^{m_\varepsilon }]. \end{aligned}$$

#### Proof

Up to (2.15) the estimates are analogous to Hensel, Liu [22, Lemma 5]. Note that one only uses the definitions and the elementary estimate $|\xi |\le 1- c\min \{d_{\Gamma ^{m_\varepsilon }}^2,1\}$, cf. also Lemma 2.2 below. The new estimate (2.15) is a simple consequence of the properties (2.4) of $\partial _n$ and $\nabla _\tau$. $\square$

#### Lemma 2.2

(Properties of $\xi$ and *B*) For $(\xi ,B)=(\xi ,B)(m_\varepsilon )$ from (2.11) we have

1. Regularity: for some $p>d+5$ it holds that
  $$\begin{aligned}&\xi \in C^1([0,T_0],C^0({\overline{\Omega }})^d) \cap C^0([0,T_0];C^2_c(\Omega )^d),\\&B\in C^0([0,T_0];C^{0,1}(\Omega )^d)\cap L^p(0,T_0;W^2_p(\Omega \setminus \Gamma ^{m_\varepsilon }_t)^d), \quad \\&\quad \nabla _\tau \nabla B\in L^\infty \left( \Gamma ^{m_\varepsilon }\left( \tfrac{3\delta }{2}\right) \right) ^{d^3}, \end{aligned}$$
  we have uniform bounds with respect to $m_\varepsilon$ and $B|_{\partial \Omega }=0$.
2. Coercivity and consistency: we have
  2.18 $$\begin{aligned} |\xi |&\le 1- c\min \{d_{\Gamma ^{m_\varepsilon }}^2,1\}&\quad&\text { in }\Omega \times [0,T], \end{aligned}$$
  2.19 $$\begin{aligned} \xi&={\textbf {n}} , \quad \nabla \cdot \xi = -H&\quad&\text { on }\Gamma ^{m_\varepsilon }, \end{aligned}$$
  2.20 $$\begin{aligned} \left| \frac{B-{\textbf{v}}^{m_\varepsilon }}{m_\varepsilon }\cdot \xi + \nabla \cdot \xi \right|&\le C\min \{d_{\Gamma ^{m_\varepsilon }},1\}&\quad&\text { a.e. in }\Omega \times [0,T], \end{aligned}$$
  where (2.19) is a relation for the mean curvature and (2.20) is an approximate equation for interface normal velocity.
3. Evolution equations for $\xi$: it holds (where $\nabla B$ is defined to be the Jacobian) that
  2.21 $$\begin{aligned} |(\partial _t+B\cdot \nabla )|\xi |^2|&\le C\min \{d_{\Gamma ^{m_\varepsilon }}^2,1\}  &   \text{ a.e. } \text{ in } \Omega \times [0,T], \end{aligned}$$
  2.22 $$\begin{aligned} |\partial _t\xi + (B\cdot \nabla )\xi + (Id -\xi \otimes \xi )(\nabla B)^\top \xi |&\le C\min \{d_{\Gamma ^{m_\varepsilon }},1\}  &   \text{ a.e. } \text{ in } \Omega \times [0,T], \end{aligned}$$

where (2.21) means that $|\xi |^2$ is approximately transported by the vector field *B* and (2.22) that $\xi$ is approximately transported and rotated by *B*.

#### Proof

The regularity and uniform bounds for $\xi$ are obtained directly from the ones for $d_{\Gamma ^{m_\varepsilon }}$ in Theorem 7.9. Moreover, ${\textbf{v}}^{m_\varepsilon }$ instead of *B* satisfies the regularity, uniform bounds and the boundary condition stated for *B* due to Theorem 7.9 together with embeddings and interpolation theory. Moreover, the second term in the definition (2.11) of *B* is contained in $C^0([0,T_0],C_c^2(\Omega )^2)$ because of Theorem 7.9 and due to the $m_\varepsilon$-prefactor and the well-known identities $H=(-\Delta d_{\Gamma ^{m_\varepsilon }})|_{P_{\Gamma ^{m_\varepsilon }}}$, ${\textbf {n}} =\nabla d_{\Gamma ^{m_\varepsilon }}$ and $P_{\Gamma ^{m_\varepsilon }}=Id -{\textbf {n}} d_{\Gamma ^{m_\varepsilon }}$.

The properties (2.18)–(2.19) are clear from the definition and the well-known identities ${\textbf {n}} =\nabla d_{\Gamma ^{m_\varepsilon }}$, $\nabla \cdot {\textbf {n}} =\Delta d_{\Gamma ^{m_\varepsilon }}$ on $\Gamma ^{m_\varepsilon }(2\delta )$ and $\Delta d_{\Gamma ^{m_\varepsilon }}|_{\Gamma ^{m_\varepsilon }}=-H$ on $\Gamma ^{m_\varepsilon }$. Moreover, a direct calculation gives

$$\begin{aligned} \frac{B-{\textbf{v}}^{m_\varepsilon }}{m_\varepsilon }\cdot \xi + \nabla \cdot \xi = H \eta _{m_\varepsilon }{\tilde{\eta }}_{m_\varepsilon } + \nabla \cdot \xi . \end{aligned}$$

The latter vanishes on $\Gamma ^{m_\varepsilon }$, hence (2.20) follows. Moreover,

2.23 $$\begin{aligned} -\partial _td_{\Gamma ^{m_\varepsilon }}=V_{\Gamma ^{m_\varepsilon }}={\textbf{v}}^{m_\varepsilon }\cdot {\textbf {n}} + m_\varepsilon H = B\cdot \nabla d_{\Gamma ^{m_\varepsilon }}\quad \text { on }\Gamma ^{m_\varepsilon } \end{aligned}$$

due to (7.6) for $m_\varepsilon$ instead of *m*, and therefore

2.24 $$\begin{aligned} (\partial _t+B\cdot \nabla )d_{\Gamma ^{m_\varepsilon }}=0\quad \text { on }\Gamma ^{m_\varepsilon }. \end{aligned}$$

We compute $|\xi |^2=\eta _{m_\varepsilon }^2$ and

$$\begin{aligned} (\partial _t+B\cdot \nabla )|\xi |^2=\frac{2}{\delta }\eta _{m_\varepsilon } {\bar{\eta }}'\left( \frac{d_{\Gamma ^{m_\varepsilon }}}{\delta }\right) (\partial _t+B\cdot \nabla )d_{\Gamma ^{m_\varepsilon }}. \end{aligned}$$

Due to (2.24), ${\bar{\eta }}'(0)=0$ together with a transformation in tubular neighbourhood coordinates and the Taylor Theorem, we obtain (2.21).

Finally, let us prove (2.22). Equation (2.23) yields, by definition,

$$\begin{aligned} \partial _td_{\Gamma ^{m_\varepsilon }}+{\textbf{v}}^{m_\varepsilon }|_{P_{\Gamma ^{m_\varepsilon }}}\cdot {\textbf {n}} + m_\varepsilon H = 0 \quad \text { on }\Gamma ^{m_\varepsilon }(2\delta ). \end{aligned}$$

Differentiating the previous identity implies

2.25 $$\begin{aligned} \partial _t{\textbf {n}} +\nabla ({\textbf{v}}^{m_\varepsilon }|_{P_{\Gamma ^{m_\varepsilon }}})^\top \cdot {\textbf {n}} + (\nabla {\textbf {n}} )^\top {\textbf{v}}^{m_\varepsilon }|_{P_{\Gamma ^{m_\varepsilon }}} + m_\varepsilon \nabla H = 0 \quad \text { on }\Gamma ^{m_\varepsilon }(2\delta ), \end{aligned}$$

where $\nabla ({\textbf{v}}^{m_\varepsilon }|_{P_{\Gamma ^{m_\varepsilon }}})=\nabla {\textbf{v}}^{m_\varepsilon }|_{P_{\Gamma ^{m_\varepsilon }}}\nabla P_{\Gamma ^{m_\varepsilon }}$ and it is well-known that $\nabla P_{\Gamma ^{m_\varepsilon }}|_{\Gamma ^{m_\varepsilon }}=Id -{\textbf {n}} \otimes {\textbf {n}}$ on $\Gamma ^{m_\varepsilon }$. Moreover, on $\Gamma ^{m_\varepsilon }$ it holds that

$$\begin{aligned}&\partial _t\xi + (B\cdot \nabla )\xi + (Id -\xi \otimes \xi )(\nabla B)^\top \xi \\&= \partial _t{\textbf {n}} + ({\textbf{v}}^{m_\varepsilon }+m_\varepsilon H{\textbf {n}} )\cdot \nabla {\textbf {n}} +(Id -{\textbf {n}} \otimes {\textbf {n}} )\left[ \nabla {\textbf{v}}^{m_\varepsilon }+m_\varepsilon ({\textbf {n}} \nabla H^\top +H\nabla {\textbf {n}} )\right] ^\top {\textbf {n}} \\&=\partial _t{\textbf {n}} + {\textbf{v}}^{m_\varepsilon }\cdot \nabla {\textbf {n}} +(Id -{\textbf {n}} \otimes {\textbf {n}} )(\nabla {\textbf{v}}^{m_\varepsilon })^\top {\textbf {n}} +m_\varepsilon \nabla H, \end{aligned}$$

where we used $(\nabla {\textbf {n}} )^\top {\textbf {n}} =0$ and ${\textbf {n}} \cdot \nabla {\textbf {n}} ={\textbf {n}} \cdot \nabla H=0$. Finally, due to ${\textbf {n}} =\nabla d_{\Gamma ^{m_\varepsilon }}$ on $\Gamma ^{m_\varepsilon }(2\delta )$ we have $\partial _{x_j}{\textbf {n}} =\nabla n_j$ for $j=1,...,d$ and hence ${\textbf{v}}^{m_\varepsilon }\cdot \nabla {\textbf {n}} =(\nabla {\textbf {n}} )^\top {\textbf{v}}^{m_\varepsilon }$ on $\Gamma ^{m_\varepsilon }(2\delta )$. Therefore we obtain (2.22) from (2.25). $\square$

### Bulk Error Functional

We define the bulk error functional for all $t\in [0,T_0]$ by

2.26 $$\begin{aligned} E_bulk [\varphi _\varepsilon |\Gamma ^{m_\varepsilon }](t):=\int _\Omega \left( \sigma \chi _{\Omega ^{m_\varepsilon ,+}_t} -\psi _\varepsilon (x,t)\right) \vartheta (x,t)\,\textrm{d}x, \end{aligned}$$

where $\vartheta :{\overline{\Omega }}\times [0,T_0]\rightarrow [0,1]$ is defined as $\vartheta :={\overline{\vartheta }}(\frac{d_{\Gamma ^{m_\varepsilon }}}{\delta })$, where ${\overline{\vartheta }}:{\mathbb {R}}\rightarrow [0,1]$ is smooth with

$$\begin{aligned} {\overline{\vartheta }}>0\text { on }(1,\infty ),\quad {\overline{\vartheta }}<0\text { on }(-\infty ,0)\quad \text { and }|{\overline{\vartheta }}|=1\text { on }{\mathbb {R}}\setminus [-1,1], \end{aligned}$$

as well as $c_{{\overline{\vartheta }}}|r|\le |{\overline{\vartheta }}(r)|\le C_{{\overline{\vartheta }}}|r|$ for all $r\in [-1,1]$ and some $c_{{\overline{\vartheta }}},C_{{\overline{\vartheta }}}>0$. Note that we use a different sign convention for ${\overline{\vartheta }}$ as in [22]. Hence $\vartheta$ is roughly proportional to the signed distance function of $\Gamma ^{m_\varepsilon }$ close to $\Gamma ^{m_\varepsilon }$ and appropriately truncated to $\pm 1$ outside. The required properties of $\vartheta$ will be shown in Lemma 2.4 below.

Let us now prove coercivity properties for the bulk error functional.

#### Lemma 2.3

(Coercivity Properties of $E_\text {bulk}$) It holds $\left( \sigma \chi _{\Omega ^{m_\varepsilon ,+}} -\psi _\varepsilon \right) \vartheta \ge 0$, in particular $E_bulk [\varphi _\varepsilon |\Gamma ^{m_\varepsilon }]\ge 0$. Moreover,

2.27 $$\begin{aligned}&\int _\Omega \left| \sigma \chi _{\Omega ^{m_\varepsilon ,+}} -\psi _\varepsilon \right| \min \{d_{\Gamma ^{m_\varepsilon }},1\}\,\text {d}x \nonumber \\  &\quad + \Vert \sigma \chi _{\Omega ^{m_\varepsilon ,+}} -\psi _\varepsilon \Vert _{L^1(\Omega )}^2 \le C E_bulk [\varphi _\varepsilon |\Gamma ^{m_\varepsilon }]. \end{aligned}$$

Finally, for all $c_0>0$ there exists $C=C(c_0)>0$ such that

2.28 $$\begin{aligned} \begin{aligned}&\int _\Omega |\sigma \chi _{\Omega ^{m_\varepsilon ,+}} -\psi _\varepsilon ||{{\textbf {v}}}_\varepsilon -{{\textbf {v}}}^{m_\varepsilon }|\,\text {d}x\\  &\quad \le c_0\int _\Omega |\nabla {{\textbf {v}}}_\varepsilon -\nabla {{\textbf {v}}}^{m_\varepsilon }|^2\,\text {d}x + C\left( E[{{\textbf {v}}}_\varepsilon ,\varphi _\varepsilon | {{\textbf {v}}}^{m_\varepsilon },\Gamma ^{m_\varepsilon }] + E_bulk [\varphi _\varepsilon |\Gamma ^{m_\varepsilon }]\right) .\end{aligned} \end{aligned}$$

#### Proof

Because of $|\varphi _\varepsilon |\le 1$ due to the maximum principle, it holds that $|\psi _\varepsilon |\le \sigma$, by definition. Hence the properties of ${\overline{\vartheta }}$ yield $\left( \sigma \chi _{\Omega ^{m_\varepsilon ,+}} -\psi _\varepsilon \right) \vartheta \ge 0$. Moreover,

$$\begin{aligned} |\sigma \chi _{\Omega ^{m_\varepsilon ,+}} -\psi _\varepsilon |\min \{d_{\Gamma ^{m_\varepsilon }},1\}\le C|\sigma \chi _{\Omega ^{m_\varepsilon ,+}} -\psi _\varepsilon ||\vartheta |=C\left( \sigma \chi _{\Omega ^{m_\varepsilon ,+}} -\psi _\varepsilon \right) \vartheta \end{aligned}$$

and this estimates the first term in (2.27). This yields that the second term in (2.27) is controlled by using the inequality (cf. [17, Proof of Theorem 1])

2.29 $$\begin{aligned} \left( \int _0^\delta |g|\,dr\right) ^2\le 2\Vert g\Vert _{L^\infty (0,\delta )}\int _0^\delta |g|(r)r\,dr\quad \text { for all }g\in L^\infty (0,\delta ), \end{aligned}$$

which is derived by dividing the square $[0,\delta ]^2$ into two triangles and applying Fubini’s theorem.

Finally, (2.28) can be shown analogously to [22, proof of (31)] with (2.27) and elementary estimates, in particular the Gagliardo-Nirenberg inequality in normal direction of $\Gamma ^{m_\varepsilon }$, the Hölder and Young inequality as well as (2.29). $\square$

#### Lemma 2.4

(Properties of $\vartheta$) For $\vartheta =\vartheta (m_\varepsilon )$ defined after (2.26) the following properties hold:

1. Regularity: it holds that
  $$\begin{aligned} \vartheta \in C^1([0,T_0],C^1({\overline{\Omega }})) \cap C^0([0,T_0];C^3({\overline{\Omega }})) \end{aligned}$$
  and we have uniform bounds with respect to $m_\varepsilon$.
2. Coercivity and consistency: we have
  2.30 $$\begin{aligned}&c\min \{d_{\Gamma ^{m_\varepsilon }},1\}\le |\vartheta |\le C\min \{d_{\Gamma ^{m_\varepsilon }},1\}\quad \text {for some }c,C>0, \end{aligned}$$
  2.31 $$\begin{aligned}&\vartheta >0\text { in }\Omega ^{m_\varepsilon ,+},\quad \vartheta <0\text { in }\Omega ^{m_\varepsilon ,-}. \end{aligned}$$
3. Evolution equation: it holds that
  2.32 $$\begin{aligned} |(\partial _t+B\cdot \nabla )\vartheta |\le C\min \{d_{\Gamma ^{m_\varepsilon }},1\}\quad \text { a.e. on }\Omega \times (0,T_0). \end{aligned}$$

#### Proof

The regularity and uniform bounds for $\vartheta$ follow directly from the ones for $d_{\Gamma ^{m_\varepsilon }}$ by Theorem 7.9. The estimates (2.30)–(2.31) follow directly from the definition of $\vartheta$ and the properties of ${\overline{\vartheta }}$. Moreover, (2.32) is shown via the chain rule and $(\partial _t+B\cdot \nabla )d_{\Gamma ^{m_\varepsilon }}=0$ on $\Gamma ^{m_\varepsilon }$, cf. (2.24). $\square$

## Stability Estimate and Convergence Result

In this section we formulate our main results on stability and quantitative convergence for solutions of the Navier–Stokes/Allen–Cahn system (1.1a)–(1.1e) towards solutions of the classical two-phase Navier–Stokes equation (1.2a)–(1.2g) for suitable scalings of the mobility $m_\varepsilon$.

We obtain the following stability result:

### Theorem 3.1

(**Stability Estimate**) Let $m_\varepsilon :=m_0\varepsilon ^\beta >0$, where $m_0>0$ and $\beta \in (0,2)$ are fixed. Let $T_0>0$ be such that the two-phase Navier–Stokes system with surface tension (1.2a)–(1.2g) has a smooth solution $({\textbf{v}}_0^\pm ,p_0^\pm ,\Gamma )$ on $[0,T_0]$. Moreover, let $({\textbf{v}}_{m_\varepsilon }^\pm ,p_{m_\varepsilon }^\pm ,\Gamma ^{m_\varepsilon })$ be strong solutions to the modified system (7.1)–(7.7) for $\varepsilon >0$ small, cf. Theorem 7.9 below. Furthermore, let $({\textbf{v}}_\varepsilon , \varphi _\varepsilon )$ for $\varepsilon >0$ be energy-dissipating weak solutions to the Navier–Stokes/Allen–Cahn system (1.1a)–(1.1e) on $[0,T_0]$ for the constant mobility $m_\varepsilon$ as in Remark 1.2 starting from initial data with energy uniformly bounded with respect to $\varepsilon$. We use the notation from Sect. 2, in particular we define the relative energy functional $E[{\textbf{v}}_\varepsilon ,\varphi _\varepsilon | {\textbf{v}}^{m_\varepsilon },\Gamma ^{m_\varepsilon }]$ and the bulk error functional $E_bulk [\varphi _\varepsilon |\Gamma ^{m_\varepsilon }]$ as in (2.8) and (2.26).

Then for $\varepsilon >0$ small and a.e. $T\in [0,T_0]$ it holds that

3.1 $$\begin{aligned} \begin{aligned}&\frac{1}{2}\Vert \nabla {\textbf{v}}_\varepsilon -\nabla {\textbf{v}}^{m_\varepsilon }\Vert _{L^2(0,T;L^2(\Omega ))}^2+E[{\textbf{v}}_\varepsilon ,\varphi _\varepsilon | {\textbf{v}}^{m_\varepsilon },\Gamma ^{m_\varepsilon }](T)+E_bulk [\varphi _\varepsilon |\Gamma ^{m_\varepsilon }](T)\\&\le e^{C(\beta ,m_0)T}\left( E[{\textbf{v}}_\varepsilon ,\varphi _\varepsilon | {\textbf{v}}^{m_\varepsilon },\Gamma ^{m_\varepsilon }](0)+E_bulk [\varphi _\varepsilon |\Gamma ^{m_\varepsilon }](0)+C\frac{\varepsilon ^2}{m_\varepsilon }\right) .\end{aligned} \end{aligned}$$

The proof is done via a Gronwall-type argument in Sect. 6, using coercivity properties for the relative entropy and the bulk error in Sect. 2 as well as preliminary estimates in Sects. 4 and 2.2. As an immediate consequence of Theorem 3.1 we get,

### Corollary 3.2

(Convergence Result) Let the assumptions of Theorem 3.1 hold. Moreover, let the initial data satisfy

3.2 $$\begin{aligned} E[{\textbf{v}}_\varepsilon ,\varphi _\varepsilon | {\textbf{v}}^0,\Gamma ](0)+E_bulk [\varphi _\varepsilon |\Gamma ](0) \le C\frac{\varepsilon ^2}{m_\varepsilon } \end{aligned}$$

for $\varepsilon >0$ small. Then for $\varepsilon >0$ small and a.e. $T\in [0,T_0]$ it holds that

3.3 $$\begin{aligned} \begin{aligned} \Vert ({\textbf{v}}_\varepsilon -{\textbf{v}}^{m_\varepsilon })(.,T)\Vert _{L^2(\Omega )} + \Vert \sigma \chi _{\Omega ^{m_\varepsilon ,+}_T} -\psi _\varepsilon (.,T)\Vert _{L^1(\Omega )}&\le Ce^{C(\beta ,m_0)T}\frac{\varepsilon }{\sqrt{m_\varepsilon }},\\ \Vert \nabla {\textbf{v}}_\varepsilon -\nabla {\textbf{v}}^{m_\varepsilon }\Vert _{L^2(0,T;L^2(\Omega ))}&\le Ce^{C(\beta ,m_0)T}\frac{\varepsilon }{\sqrt{m_\varepsilon }}\end{aligned} \end{aligned}$$

and for the true limit we obtain

3.4 $$\begin{aligned} \begin{aligned} \Vert ({\textbf{v}}_\varepsilon -{\textbf{v}}^0)(.,T)\Vert _{L^2(\Omega )} + \Vert \sigma \chi _{\Omega ^+_T} -\psi _\varepsilon (.,T)\Vert _{L^1(\Omega )}&\le C\left( e^{C(\beta ,m_0)T}\frac{\varepsilon }{\sqrt{m_\varepsilon }}+m_\varepsilon \right) ,\\ \Vert \nabla {\textbf{v}}_\varepsilon -\nabla {\textbf{v}}^0\Vert _{L^2(0,T;L^2(\Omega ))}&\le C\left( e^{C(\beta ,m_0)T}\frac{\varepsilon }{\sqrt{m_\varepsilon }}+m_\varepsilon \right) .\end{aligned} \end{aligned}$$

Finally, there are well-prepared initial data $({\textbf{v}}_{0,\varepsilon },c_{0,\varepsilon })$ for $\varepsilon >0$ small in the sense that (3.2) is satisfied, even with rate $\varepsilon ^2$.

### Proof

Estimate (3.3) is a direct consequence of Theorem 3.1 and the coercivity estimates (2.12) from Lemma 2.1 and (2.27) from Lemma 2.3. Then (3.4) follows from Theorem 7.7. Because of $({\textbf{v}}_{m_\varepsilon }^\pm ,\Gamma ^{m_\varepsilon })(0)=({\textbf{v}}_{0,0}^\pm ,\Gamma _0)$, we conclude that $E[{\textbf{v}}_\varepsilon ,\varphi _\varepsilon | {\textbf{v}}^{m_\varepsilon },\Gamma ^{m_\varepsilon }](0)=E[{\textbf{v}}_\varepsilon ,\varphi _\varepsilon | {\textbf{v}}^0,\Gamma ](0)$ and $E_bulk [\varphi _\varepsilon |\Gamma ^{m_\varepsilon }](0)=E_bulk [\varphi _\varepsilon |\Gamma ](0)$. The existence of well-prepared initial data is well-known, cf. [17, Proof of Theorem 1] and [23, Appendix B]. $\square$

## Relative Entropy Estimate

Let the assumptions and notation of Sect. 2 be in place. In this section we derive an inequality for the relative entropy $E[{\textbf{v}}_\varepsilon ,\varphi _\varepsilon | {\textbf{v}}^{m_\varepsilon },\Gamma ^{m_\varepsilon }]$ defined in (2.8) that can be employed later to obtain a Gronwall-type estimate. We use the notation

4.1 $$\begin{aligned} H_\varepsilon := -\varepsilon \Delta \varphi _\varepsilon + \frac{1}{\varepsilon } W'(\varphi _\varepsilon ). \end{aligned}$$

### Preliminary Relative Entropy Inequality

We derive the first important estimate for the relative entropy.

#### Lemma 4.1

(Relative Entropy Inequality) Let the assumptions and notation of Sect. 2 be valid and $H_\varepsilon$ be defined as in (4.1). More precisely, let $({\textbf{v}}_{m_\varepsilon }^\pm ,p_{m_\varepsilon }^\pm , (\Gamma _t^{m_\varepsilon })_{t\in [0,T_0]})$ for $m_\varepsilon >0$ small be solutions of the adjusted two-phase Navier–Stokes equation (7.1)–(7.7) on $[0,T_0]$, cf. Theorem 7.9 below. Additionally, let $({\textbf{v}}_\varepsilon , \varphi _\varepsilon )$ for $\varepsilon >0$ small be energy dissipating weak solutions to the Navier–Stokes/Allen–Cahn system (1.1a)–(1.1e) on $[0,T_0]$ with constant mobility $m_\varepsilon >0$ as in Remark 1.2. Finally, let $\Gamma ^{m_\varepsilon }, \Omega ^{m_\varepsilon ,\pm }, {\textbf{v}}^{m_\varepsilon }$ be as in (2.1), $\sigma$, $\psi _\varepsilon$, ${\textbf {n}} _\varepsilon$ be as in (2.6)–(2.7), $\xi$, *B* be as in (2.11), $E[{\textbf{v}}_\varepsilon ,\varphi _\varepsilon | {\textbf{v}}^{m_\varepsilon },\Gamma ^{m_\varepsilon }]$ be as in (2.8). Then for a.e. $T\in [0,T_0]$ we obtain

4.2a $$\begin{aligned}&E[{\textbf{v}}_\varepsilon ,\varphi _\varepsilon | {\textbf{v}}^{m_\varepsilon },\Gamma ^{m_\varepsilon }](T)\le E[{\textbf{v}}_\varepsilon ,\varphi _\varepsilon | {\textbf{v}}^{m_\varepsilon },\Gamma ^{m_\varepsilon }](0)-\int _0^T\int _\Omega |\nabla {\textbf{v}}_\varepsilon -\nabla {\textbf{v}}^{m_\varepsilon }|^2\,\textrm{d}x\,\textrm{d}t\nonumber \\&\quad - \int _0^T \int _\Omega \frac{m_\varepsilon }{2\varepsilon }\left| H_\varepsilon + \sqrt{2W(\varphi _\varepsilon )}\nabla \cdot \xi \right| ^2 \textrm{d}x\,\textrm{d}t \end{aligned}$$

4.2b $$\begin{aligned}&\quad - \int _0^T \int _\Omega \frac{m_\varepsilon }{2\varepsilon }\left| H_\varepsilon - \frac{B-{\textbf{v}}^{m_\varepsilon }}{m_\varepsilon }\cdot \xi \,\varepsilon |\nabla \varphi _\varepsilon |\right| ^2 \textrm{d}x\,\textrm{d}t \end{aligned}$$

4.2c $$\begin{aligned}&\quad - \int _0^T \int _\Omega ({\textbf{v}}_\varepsilon -{\textbf{v}}^{m_\varepsilon })\cdot (({\textbf{v}}_\varepsilon -{\textbf{v}}^{m_\varepsilon })\cdot \nabla ){\textbf{v}}^{m_\varepsilon }\,\textrm{d}x\,\textrm{d}t\nonumber \\&\quad - \int _0^T \int _\Omega (\sigma \chi _{\Omega ^{m_\varepsilon ,+}}-\psi _\varepsilon ) (({\textbf{v}}_\varepsilon -{\textbf{v}}^{m_\varepsilon })\cdot \nabla )(\nabla \cdot \xi )\,\textrm{d}x\,\textrm{d}t\nonumber \\&\quad + \int _0^T \int _\Omega m_\varepsilon \left| \frac{B-{\textbf{v}}^{m_\varepsilon }}{m_\varepsilon }\cdot \xi + \nabla \cdot \xi \right| ^2 \varepsilon |\nabla \varphi _\varepsilon |^2\,\textrm{d}x\,\textrm{d}t \end{aligned}$$

4.2d $$\begin{aligned}&\quad + \int _0^T \int _\Omega m_\varepsilon |\nabla \cdot \xi |^2\left( \frac{\sqrt{2W(\varphi _\varepsilon )}}{\sqrt{\varepsilon }} - \sqrt{\varepsilon }|\nabla \varphi _\varepsilon |\right) ^2 \textrm{d}x\,\textrm{d}t\nonumber \\&\quad - \int _0^T \int _\Omega \frac{1}{\sqrt{\varepsilon }}\left( H_\varepsilon +\sqrt{2W(\varphi _\varepsilon )}\nabla \cdot \xi \right) ({\textbf{v}}^{m_\varepsilon }-B)\cdot ({\textbf {n}} _\varepsilon -\xi )\sqrt{\varepsilon }|\nabla \varphi _\varepsilon |\,\textrm{d}x\,\textrm{d}t \end{aligned}$$

4.2e $$\begin{aligned}&\quad - \int _0^T \int _\Omega (\partial _t\xi +(B\cdot \nabla )\xi +(Id -\xi \otimes \xi )(\nabla B)^\top \xi )\cdot ({\textbf {n}} _\varepsilon -\xi )|\nabla \psi _\varepsilon |\,\textrm{d}x\,\textrm{d}t\nonumber \\&\quad - \int _0^T \int _\Omega (\xi \otimes \xi (\nabla B)^\top \xi )\cdot ({\textbf {n}} _\varepsilon -\xi )|\nabla \psi _\varepsilon |\,\textrm{d}x\,\textrm{d}t \end{aligned}$$

4.2f $$\begin{aligned}&\quad - \int _0^T \int _\Omega \left( (\partial _t+B\cdot \nabla )|\xi |^2\right) |\nabla \psi _\varepsilon |\,\textrm{d}x\,\textrm{d}t\nonumber \\&\quad - \int _0^T \int _\Omega \nabla B:(\xi -{\textbf {n}} _\varepsilon )\otimes (\xi -{\textbf {n}} _\varepsilon )|\nabla \psi _\varepsilon |\,\textrm{d}x\,\textrm{d}t\nonumber \\&\quad + \int _0^T \int _\Omega (\nabla \cdot B)(1-\xi \cdot {\textbf {n}} _\varepsilon )|\nabla \psi _\varepsilon |\,\textrm{d}x\,\textrm{d}t\nonumber \\&\quad + \int _0^T \int _\Omega (\nabla \cdot B)\frac{1}{2}\left( \sqrt{\varepsilon }|\nabla \varphi _\varepsilon |-\frac{1}{\sqrt{\varepsilon }}\sqrt{2W(\varphi _\varepsilon )}\right) ^2 \textrm{d}x\,\textrm{d}t\nonumber \\&\quad - \int _0^T \int _\Omega ({\textbf {n}} _\varepsilon \otimes {\textbf {n}} _\varepsilon -\xi \otimes \xi ):\nabla B(\varepsilon |\nabla \varphi _\varepsilon |^2-|\nabla \psi _\varepsilon |)\,\textrm{d}x\,\textrm{d}t\nonumber \\&\quad - \int _0^T \int _\Omega \xi \otimes \xi :\nabla B(\varepsilon |\nabla \varphi _\varepsilon |^2-|\nabla \psi _\varepsilon |)\,\textrm{d}x\,\textrm{d}t. \end{aligned}$$

#### Proof

Unlike in [22], our choice of *B* does not have compact support, but due to the boundary condition $B|_{\partial \Omega }=0$ by Lemma 2.2 we observe that analogous computations as in the proof of [22, Proposition 6] may be carried out. $\square$

#### Remark 4.2

The choice of *B* in (2.11), i.e. $B:={\textbf{v}}^{m_\varepsilon }+m_\varepsilon H{\textbf {n}} {\tilde{\eta }}_{m_\varepsilon }$ with the plateau cutoff ${\tilde{\eta }}_{m_\varepsilon }$, is natural in order to control the term (4.2c), i.e. $\int _0^T \int _\Omega m_\varepsilon \bigg |\frac{B-{\textbf{v}}^{m_\varepsilon }}{m_\varepsilon }\cdot \xi + \nabla \cdot \xi \bigg |^2 \varepsilon |\nabla \varphi _\varepsilon |^2\,\textrm{d}x\,\textrm{d}t$. Note that in Hensel, Liu [22] the projected velocity field $({\textbf {n}} \cdot {\textbf{v}}^{m_\varepsilon }|_{P_{\Gamma ^{m_\varepsilon }}}){\textbf {n}}$ is used within the definition of *B* instead. This is not possible here because one would then obtain from (4.2c) a remainder of the form

$$\begin{aligned} { \frac{1}{m_\varepsilon }\int _0^T\int _\Omega \min \{d_{\Gamma ^{m_\varepsilon }}^2,1\}\varepsilon |\nabla \varphi _\varepsilon |^2\,\textrm{d}x\,\textrm{d}t,} \end{aligned}$$

which is only controlled by $\frac{C}{m_\varepsilon }\int _0^T E[\varphi _\varepsilon |\Gamma ^{m_\varepsilon }](t)\,dt$ due to (2.16). However, with the new choice of *B* it is not clear anymore how to estimate the last term (4.2f), i.e., 

$$\begin{aligned} { \int _0^T \int _\Omega \xi \otimes \xi :\nabla B(\varepsilon |\nabla \varphi _\varepsilon |^2-|\nabla \psi _\varepsilon |)\,\textrm{d}x\,\textrm{d}t.} \end{aligned}$$

To this end we rewrite and estimate the term (4.2f) in a novel way in the following Sect. 4.2. The idea is to write $\xi \otimes \xi :\nabla B$ as a normal derivative and use integration by parts in a suitable way. The other terms in the relative entropy estimate from Lemma 4.1 will turn out to be controllable with the choice of *B* in (2.11), the coercivity properties of the relative entropy and the bulk error functional from Sects. 2.1 and 2.2, cf. Sect. 6 below.

### The Remaining Problematic Term

For a.e. $t\in [0,T_0]$ consider a 1-Lipschitz-function $h=h_\varepsilon (.,t):\Gamma ^{m_\varepsilon }_t\rightarrow (-\delta ,\delta )$. This function *h* will be constructed in Sect. 4.3 below, and it will be used to approximate a suitable level set of $\varphi _\varepsilon (.,t)$. Moreover, the energy outside a strip around the graph

$$\begin{aligned} \Gamma ^{m_\varepsilon }_{t,h}:=\{s+h_\varepsilon (s,t){\textbf {n}} (s,t):s\in \Gamma _t^{m_\varepsilon }\} \end{aligned}$$

over $\Gamma ^{m_\varepsilon }_t$ determined by *h* will be estimated in Sect. 4.4 below. Let us define shifted tubular neighbourhoods for a.e. $t\in [0,T_0], \tilde{\delta }\in (0,\delta ]$:

4.3 $$\begin{aligned} \Gamma ^{m_\varepsilon }_{t,h}({\tilde{\delta }}):=\{x\in \Gamma ^{m_\varepsilon }_t(2\delta ):d_{\Gamma ^{m_\varepsilon }}(x,t)\in h_\varepsilon (P_{\Gamma ^{m_\varepsilon }}(x,t),t)+(-{\tilde{\delta }},{\tilde{\delta }})\}. \end{aligned}$$

In order to estimate the problematic term from Remark 4.2, we need the following lemma whose proof is based on integration by parts and will be postponed.

#### Lemma 4.3

For a.e. $t\in [0,T_0]$ let $h=h_\varepsilon (.,t):\Gamma ^{m_\varepsilon }_t\rightarrow (-\delta ,\delta )$ be a 1-Lipschitz-function. Moreover, let $\delta _\varepsilon \in (0,\frac{\delta }{2}]$ for $\varepsilon >0$ small, $\Gamma ^{m_\varepsilon }_{t,h}(\delta _\varepsilon )$ be defined as in (4.3) and ${\tilde{\eta }}\in C^{0,1}_c(\Gamma ^{m_\varepsilon }_{t,h}(\delta _\varepsilon ))$. Then for a.e. $t\in [0,T_0]$ it holds that

4.4a $$\begin{aligned}&\int _{\Gamma ^{m_\varepsilon }_{t,h}(\delta _\varepsilon )} \left( \frac{\varepsilon }{2}|\partial _n\varphi _\varepsilon |^2-\frac{1}{\varepsilon }W(\varphi _\varepsilon )\right) \partial _n{\tilde{\eta }}\,\textrm{d}x \end{aligned}$$

4.4b $$\begin{aligned}&\quad =\int _{\Gamma ^{m_\varepsilon }_{t,h}(\delta _\varepsilon )}\left( \frac{\varepsilon }{2}|\partial _n\varphi _\varepsilon |^2+\frac{1}{\varepsilon }W(\varphi _\varepsilon )- \sqrt{2W(\varphi _\varepsilon )} \partial _n\varphi _\varepsilon \right) {\tilde{\eta }} \nabla \cdot {\textbf {n}} \,\textrm{d}x \end{aligned}$$

4.4c $$\begin{aligned}&\qquad +\int _{\Gamma ^{m_\varepsilon }_{t,h}(\delta _\varepsilon )} \left( H_\varepsilon + \sqrt{2W(\varphi _\varepsilon )} \nabla \cdot {\textbf {n}} \right) \partial _n\varphi _\varepsilon {\tilde{\eta }}\,\textrm{d}x \end{aligned}$$

4.4d $$\begin{aligned}&\qquad -\int _{\Gamma ^{m_\varepsilon }_{t,h}(\delta _\varepsilon )} \varepsilon \nabla _\tau \varphi _\varepsilon \cdot \nabla _\tau {\tilde{\eta }}\,\partial _n\varphi _\varepsilon \,\textrm{d}x \end{aligned}$$

4.4e $$\begin{aligned}&\qquad -\int _{\Gamma ^{m_\varepsilon }_{t,h}(\delta _\varepsilon )} \varepsilon \nabla _\tau \varphi _\varepsilon \cdot (\nabla {\textbf {n}} ^\top \nabla _\tau \varphi _\varepsilon ){\tilde{\eta }}\,\textrm{d}x \end{aligned}$$

4.4f $$\begin{aligned}&\qquad +\int _{\Gamma ^{m_\varepsilon }_{t,h}(\delta _\varepsilon )} \frac{\varepsilon }{2}|\nabla _\tau \varphi _\varepsilon |^2 \left( {\tilde{\eta }} \nabla \cdot {\textbf {n}} + \partial _n{\tilde{\eta }}\right) \,\textrm{d}x. \end{aligned}$$

We obtain the following important estimate:

#### Lemma 4.4

For a.e. $t\in [0,T_0]$ let $h=h_\varepsilon (.,t):\Gamma ^{m_\varepsilon }_t\rightarrow (-\delta ,\delta )$ be a 1-Lipschitz-function. Moreover, let $\delta _\varepsilon \in (0,\frac{\delta }{2}]$ for $\varepsilon >0$ small and let $\Gamma ^{m_\varepsilon }_{t,h}(\delta _\varepsilon )$ be defined as in (4.3). Then for a.e. $t\in [0,T_0]$ it holds that

4.5a $$\begin{aligned}&\left| \int _{\Omega } \xi \otimes \xi :\nabla B\left( \varepsilon |\nabla \varphi _\varepsilon |^2 - |\nabla \psi _\varepsilon |\right) |_{(x,t)} \,\textrm{d}x\right| \end{aligned}$$

4.5b $$\begin{aligned}&\quad \le C\int _{\Gamma ^{m_\varepsilon }_t(2\delta )\setminus \Gamma ^{m_\varepsilon }_{t,h}(\frac{\delta _\varepsilon }{2})} \left( \varepsilon |\nabla \varphi _\varepsilon |^2+\frac{1}{\varepsilon }W(\varphi _\varepsilon )\right) |_{(x,t)}\,\textrm{d}x \end{aligned}$$

4.5c $$\begin{aligned}&\qquad +C\int _{\Gamma ^{m_\varepsilon }_{t,h}(\delta _\varepsilon )} \frac{\varepsilon }{m_\varepsilon } |\partial _n\varphi _\varepsilon |^2 |d_{\Gamma ^{m_\varepsilon }}-h(P_{\Gamma ^{m_\varepsilon }})|^2|_{(x,t)}\,\textrm{d}x \end{aligned}$$

4.5d $$\begin{aligned}&\qquad +\frac{m_\varepsilon }{4\varepsilon }\int _{\Gamma ^{m_\varepsilon }_{t,h}(\delta _\varepsilon )} \left| H_\varepsilon + \sqrt{2W(\varphi _\varepsilon )} \nabla \cdot \xi \right| ^2|_{(x,t)} \,\textrm{d}x \end{aligned}$$

4.5e $$\begin{aligned}&\qquad +C\int _{\Gamma ^{m_\varepsilon }_{t,h}(\delta _\varepsilon )}(|h|_{P_{\Gamma ^{m_\varepsilon }}}|^2+|\nabla _\tau (h|_{P_{\Gamma ^{m_\varepsilon }}})|^2)\left( \varepsilon |\nabla \varphi _\varepsilon |^2+\frac{1}{\varepsilon }W(\varphi _\varepsilon )\right) |_{(x,t)}\,\textrm{d}x \end{aligned}$$

4.5f $$\begin{aligned}&\qquad +C E[\varphi _\varepsilon | \Gamma ^{m_\varepsilon }](t), \end{aligned}$$

where $C>0$ is independent of $\varepsilon$ and *t*.

#### Proof

First of all, it suffices to show the estimate with (4.5a) replaced by

4.6 $$\begin{aligned} \left| \int _{\Gamma ^{m_\varepsilon }_t(2\delta )} \xi \otimes \xi :\nabla B \left( \frac{\varepsilon }{2}|\partial _n\varphi _\varepsilon |^2-\frac{1}{\varepsilon }W(\varphi _\varepsilon )\right) |_{(x,t)}\,\textrm{d}x\right| \end{aligned}$$

because of the cutoff for $\xi$ in (2.11), the identity

$$\begin{aligned} \varepsilon |\nabla \varphi _\varepsilon |^2-|\nabla \psi _\varepsilon | = \frac{\varepsilon }{2}|\nabla \varphi _\varepsilon |^2-\frac{1}{\varepsilon }W(\varphi _\varepsilon ) + \frac{1}{2}\left( \sqrt{\varepsilon }|\nabla \varphi _\varepsilon |-\frac{1}{\sqrt{\varepsilon }}\sqrt{2W(\varphi _\varepsilon )}\right) ^2, \end{aligned}$$

the coercivity estimate (2.14) and finally the control of the tangential gradient in (2.15) in terms of the relative entropy. In order to rewrite the expression by Lemma 4.3, we use the following idea: for a.e. $t\in [0,T_0]$ we write $\xi \otimes \xi :\nabla B(.,t)$ as $\partial _n\eta$, where $\eta =\eta (.,t)$ is defined as

$$\begin{aligned} \eta (x):= &   \int _{h(P_{\Gamma ^{m_\varepsilon }}(x,t))}^{d_{\Gamma ^{m_\varepsilon }}(x,t)} \xi \otimes \xi :\nabla B (P_{\Gamma ^{m_\varepsilon }}(x,t) \\    &   + r{\textbf {n}} (P_{\Gamma ^{m_\varepsilon }}(x,t),t),t)\,dr\quad \text { for }x\in \Gamma ^{m_\varepsilon }_t(2\delta ). \end{aligned}$$

Moreover, to avoid boundary terms on $\partial \Gamma ^{m_\varepsilon }_t(\delta _\varepsilon )$ we introduce a smooth $\alpha :{\mathbb {R}}\rightarrow [0,1]$ with $\alpha \equiv 1$ on $[-\frac{1}{2},\frac{1}{2}]$ and $\alpha \equiv 0$ on ${\mathbb {R}}\setminus [-\frac{3}{4},\frac{3}{4}]$ and set for a.e. $t\in [0,T_0]$

$$\begin{aligned} {\tilde{\eta }}= &   {\tilde{\eta }}(.,t):=({\tilde{\alpha }}\eta )(.,t),\quad {\tilde{\alpha }}(x,t)\\  := &   \alpha \left( \frac{d_{\Gamma ^{m_\varepsilon }}(x,t)-h(P_{\Gamma ^{m_\varepsilon }}(x,t))}{\delta _\varepsilon }\right) ,\quad \text { for }x\in \Gamma ^{m_\varepsilon }_t(2\delta ). \end{aligned}$$

Note that Theorem 7.9 and the regularity and uniform bounds in Theorem 2.2 yield that ${\tilde{\alpha }}(.,t)$, ${\tilde{\eta }}(.,t)$ and $\eta (.,t)$ are in $C^{0,1}({\overline{\Omega }})$. We rewrite things as follows:

4.7 $$\begin{aligned} \xi \otimes \xi :\nabla B(.,t)=\partial _n\eta =(1-{\tilde{\alpha }})\partial _n\eta - \partial _n{\tilde{\alpha }}\,\eta +\partial _n{\tilde{\eta }}. \end{aligned}$$

Because of the definition of ${\tilde{\alpha }}$, it holds that $1-{\tilde{\alpha }}=0$ on $\Gamma ^{m_\varepsilon }_{t,h}(\frac{\delta _\varepsilon }{2})$. Moreover,

$$\begin{aligned} \partial _n{\tilde{\alpha }}= &   \frac{1}{\delta _\varepsilon }\alpha '\left( \frac{d_{\Gamma ^{m_\varepsilon }}(x,t)-h(P_{\Gamma ^{m_\varepsilon }}(x,t))}{\delta _\varepsilon }\right) =0\\    &   \quad \text { on }\Gamma ^{m_\varepsilon }_{t,h}\left( \tfrac{\delta _\varepsilon }{2}\right) \cup \left[ \Gamma ^{m_\varepsilon }_t(2\delta )\setminus \Gamma ^{m_\varepsilon }_{t,h}\left( \tfrac{3}{4}\delta _\varepsilon \right) \right] \end{aligned}$$

and $|\eta |\le C\delta _\varepsilon$ on $\Gamma ^{m_\varepsilon }_{t,h}(\delta _\varepsilon )$ for a.e. $t\in [0,T_0]$. Therefore the first two terms on the right hand side of (4.7) yield contributions in (4.6) that can be estimated by (4.5b). Hence we can replace $\xi \otimes \xi :\nabla B(.,t)$ by $\partial _n{\tilde{\eta }}$ in (4.6) for a.e. $t\in [0,T_0]$.

For the remaining term

4.8 $$\begin{aligned} \left| \int _{\Gamma ^{m_\varepsilon }_{t,h}(\delta _\varepsilon )} \partial _n{\tilde{\eta }} \left( \frac{\varepsilon }{2}|\partial _n\varphi _\varepsilon |^2-\frac{1}{\varepsilon }W(\varphi _\varepsilon )\right) |_{(x,t)}\,\textrm{d}x\right| \end{aligned}$$

one can apply integration by parts on $\Gamma ^{m_\varepsilon }_{t,h}(\delta _\varepsilon )$ for a.e. $t\in [0,T_0]$ and rewrite in a suitable way, cf. Lemma 4.3. Let us estimate the corresponding terms (4.4b)–(4.4f) from Lemma 4.3 now. Note that (4.4b) and (4.4e)–(4.4f) are directly controlled by (4.5f) because of the coercivity properties (2.15) of the relative entropy.

Moreover, (4.4c) with $\nabla \cdot \xi$ instead of $\nabla \cdot {\textbf {n}}$ can be estimated by (4.5c) and (4.5d) due to Young’s inequality and $|{\tilde{\eta }}|(.,t)\le C|d_{\Gamma ^{m_\varepsilon }}-h(P_{\Gamma ^{m_\varepsilon }})|(.,t)$. Because of the definition of $\xi$ in (2.11) it holds that

$$\begin{aligned} \nabla \cdot {\textbf {n}} =(1-\eta _{m_\varepsilon }) \nabla \cdot {\textbf {n}} - \frac{1}{\delta }{\bar{\eta }}'\left( \frac{d_{\Gamma ^{m_\varepsilon }}}{\delta }\right) + \nabla \cdot \xi , \end{aligned}$$

where $|1-\eta _{m_\varepsilon }|\le C|d_{\Gamma ^{m_\varepsilon }}|^2$ and $|{\bar{\eta }}'\left( \frac{d_{\Gamma ^{m_\varepsilon }}}{\delta }\right) |\le C|d_{\Gamma ^{m_\varepsilon }}|$. Hence, from exchanging $\nabla \cdot {\textbf {n}}$ with $\nabla \cdot \xi$, we obtain the error term

$$\begin{aligned} \int _{\Gamma ^{m_\varepsilon }_{t,h}(\delta _\varepsilon )}\sqrt{W(\varphi _\varepsilon )} |\partial _n\varphi _\varepsilon |\,(|d_{\Gamma ^{m_\varepsilon }}|^2+|h(P_{\Gamma ^{m_\varepsilon }})|^2)|_{(x,t)}\,\textrm{d}x, \end{aligned}$$

which is controlled by (4.5e) and (4.5f) due to the bound $\sqrt{W(\varphi _\varepsilon )} |\partial _n\varphi _\varepsilon |\le |\nabla \psi _\varepsilon |$ and (2.16). Hence (4.4c) is estimated.

Finally, it remains to estimate (4.4d). It holds $\nabla _\tau {\tilde{\eta }}=\nabla _\tau \,{\tilde{\alpha }}\eta +{\tilde{\alpha }}\nabla _\tau \eta$ due to ${\tilde{\eta }}={\tilde{\alpha }}\eta$, where we have because of (2.5)

$$\begin{aligned} \nabla _\tau {\tilde{\alpha }}=&-\frac{1}{\delta _\varepsilon } \alpha '\left( \frac{d_{\Gamma ^{m_\varepsilon }}(.,t)-h(P_{\Gamma ^{m_\varepsilon }}(.,t))}{\delta _\varepsilon }\right) \nabla _\tau (h(P_{\Gamma ^{m_\varepsilon }}))|_{(.,t)},\\ \nabla _\tau \eta =&-\partial _n\eta |_{(P_{\Gamma ^{m_\varepsilon }}(.,t)+h(P_{\Gamma ^{m_\varepsilon }}(.,t)){\textbf {n}} (P_{\Gamma ^{m_\varepsilon }}(.,t),t)} \nabla _\tau (h(P_{\Gamma ^{m_\varepsilon }}))|_{(.,t)} \\&+ \int _{h(P_{\Gamma ^{m_\varepsilon }}(.,t))}^{d_{\Gamma ^{m_\varepsilon }}(.,t)} \nabla _\tau \left[ \xi \otimes \xi :\nabla B(P_{\Gamma ^{m_\varepsilon }}+r{\textbf {n}} (P_{\Gamma ^{m_\varepsilon }}),t)\right] |_{(.,t)}\,dr. \end{aligned}$$

Due to Lemma 2.2 we obtain

$$\begin{aligned} |\nabla _\tau {\tilde{\eta }}| \le C\left( |d_{\Gamma ^{m_\varepsilon }}|+|h|_{P_{\Gamma ^{m_\varepsilon }}}|+|\nabla _\tau (h|_{P_{\Gamma ^{m_\varepsilon }}})|\right) |_{(.,t)}. \end{aligned}$$

Altogether, (4.4d) is controlled by (4.5e) and (4.5f) due to Young’s inequality, (2.15) and (2.16). This shows Lemma 4.4. $\square$

#### Proof of Lemma 4.3

First, we use $\partial _n{\tilde{\eta }}={\textbf {n}} \cdot \nabla {\tilde{\eta }}=\sum _{i=1}^d n_i\partial _{x_i}{\tilde{\eta }}$ and integration by parts on $\Gamma ^{m_\varepsilon }_{t,h}(\delta _\varepsilon )$ with ${\tilde{\eta }}\in C^{0,1}_c(\Gamma ^{m_\varepsilon }_{t,h}(\delta _\varepsilon ))$ for a.e. $t\in [0,T_0]$. This yields

$$\begin{aligned}&\int _{\Gamma ^{m_\varepsilon }_{t,h}(\delta _\varepsilon )} \left( \frac{\varepsilon }{2}|\partial _n\varphi _\varepsilon |^2-\frac{1}{\varepsilon }W(\varphi _\varepsilon )\right) \partial _n{\tilde{\eta }}\,\textrm{d}x \\&\quad =-\int _{\Gamma ^{m_\varepsilon }_{t,h}(\delta _\varepsilon )} \left( \frac{\varepsilon }{2}|\partial _n\varphi _\varepsilon |^2-\frac{1}{\varepsilon }W(\varphi _\varepsilon )\right) (\nabla \cdot {\textbf {n}} ) {\tilde{\eta }}\,\textrm{d}x\\&\qquad -\int _{\Gamma ^{m_\varepsilon }_{t,h}(\delta _\varepsilon )} \partial _n \left( \frac{\varepsilon }{2}|\partial _n\varphi _\varepsilon |^2-\frac{1}{\varepsilon }W(\varphi _\varepsilon )\right) {\tilde{\eta }}\,\textrm{d}x \end{aligned}$$

for a.e. $t\in [0,T_0]$. Note that $\partial _n^2\varphi _\varepsilon =(({\textbf {n}} \cdot \nabla ){\textbf {n}} )\cdot \nabla {\varphi _\varepsilon } + {\textbf {n}} \otimes {\textbf {n}} :D^2\varphi _\varepsilon ={\textbf {n}} \otimes {\textbf {n}} :D^2\varphi _\varepsilon$. Therefore

$$\begin{aligned}&-\int _{\Gamma ^{m_\varepsilon }_{t,h}(\delta _\varepsilon )} \partial _n \left( \frac{\varepsilon }{2}|\partial _n\varphi _\varepsilon |^2-\frac{1}{\varepsilon }W(\varphi _\varepsilon )\right) {\tilde{\eta }}\,\textrm{d}x\\&\quad =-\int _{\Gamma ^{m_\varepsilon }_{t,h}(\delta _\varepsilon )} \left( \varepsilon \Delta \varphi _\varepsilon -\frac{1}{\varepsilon }W'(\varphi _\varepsilon )\right) \partial _n\varphi _\varepsilon {\tilde{\eta }}\,\textrm{d}x\\&\qquad +\int _{\Gamma ^{m_\varepsilon }_{t,h}(\delta _\varepsilon )} \varepsilon \left( Id -{\textbf {n}} \otimes {\textbf {n}} \right) :D^2\varphi _\varepsilon \partial _n\varphi _\varepsilon {\tilde{\eta }}\,\textrm{d}x \end{aligned}$$

for a.e. $t\in [0,T_0]$. In order to rewrite the last term, we compute

$$\begin{aligned}&\nabla \cdot \left( (Id -{\textbf {n}} \otimes {\textbf {n}} )\nabla \varphi _\varepsilon \partial _n\varphi _\varepsilon {\tilde{\eta }}\right) \\&\quad =-(\nabla \cdot {\textbf {n}} ){\textbf {n}} \cdot \nabla \varphi _\varepsilon \partial _n\varphi _\varepsilon {\tilde{\eta }} + (Id -{\textbf {n}} \otimes {\textbf {n}} ):D^2\varphi _\varepsilon \partial _n\varphi _\varepsilon {\tilde{\eta }} +\nabla _\tau \varphi _\varepsilon \cdot \nabla (\partial _n\varphi _\varepsilon {\tilde{\eta }}), \end{aligned}$$

where we used $\nabla \cdot (Id -{\textbf {n}} \otimes {\textbf {n}} )=-(\nabla \cdot {\textbf {n}} ){\textbf {n}}$. Because of

$$\begin{aligned} \nabla (\partial _n\varphi _\varepsilon )=\nabla ({\textbf {n}} \cdot \nabla )\varphi _\varepsilon =(\nabla {\textbf {n}} )^\top \nabla \varphi _\varepsilon +({\textbf {n}} \cdot \nabla )\nabla \varphi _\varepsilon \end{aligned}$$

and integration by parts, we obtain, for a.e. $t\in [0,T_0]$,

$$\begin{aligned}&\int _{\Gamma ^{m_\varepsilon }_{t,h}(\delta _\varepsilon )} \varepsilon \left( Id -{\textbf {n}} \otimes {\textbf {n}} \right) :D^2\varphi _\varepsilon \partial _n\varphi _\varepsilon {\tilde{\eta }}\,\textrm{d}x\\&\quad =\int _{\Gamma ^{m_\varepsilon }_{t,h}(\delta _\varepsilon )}\varepsilon |\partial _n\varphi _\varepsilon |^2(\nabla \cdot {\textbf {n}} ){\tilde{\eta }}\,\textrm{d}x -\int _{\Gamma ^{m_\varepsilon }_{t,h}(\delta _\varepsilon )}\varepsilon \nabla _\tau \varphi _\varepsilon \cdot \nabla {\tilde{\eta }}\,\partial _n\varphi _\varepsilon \,\textrm{d}x\\&\qquad -\int _{\Gamma ^{m_\varepsilon }_{t,h}(\delta _\varepsilon )}\varepsilon \nabla _\tau \varphi _\varepsilon \cdot (\nabla {\textbf {n}} ^\top \nabla \varphi _\varepsilon ){\tilde{\eta }}\,\textrm{d}x -\int _{\Gamma ^{m_\varepsilon }_{t,h}(\delta _\varepsilon )}\varepsilon \nabla _\tau \varphi _\varepsilon \cdot \left( ({\textbf {n}} \cdot \nabla )\nabla \varphi _\varepsilon \right) {\tilde{\eta }}\,\textrm{d}x. \end{aligned}$$

Note that in (4.4b)–(4.4c) the $\sqrt{2W(\varphi _\varepsilon )}$-terms cancel and were added for convenience. Moreover, one can directly prove that $\nabla {\textbf {n}} ^\top \nabla \varphi _\varepsilon =\nabla {\textbf {n}} ^\top \nabla _\tau \varphi _\varepsilon$. Hence it is left to show that for a.e. $t\in [0,T_0]$

4.9 $$\begin{aligned} -\int _{\Gamma ^{m_\varepsilon }_{t,h}(\delta _\varepsilon )}\varepsilon \nabla _\tau \varphi _\varepsilon \cdot \left( ({\textbf {n}} \cdot \nabla )\nabla \varphi _\varepsilon \right) {\tilde{\eta }}\,\textrm{d}x =\int _{\Gamma ^{m_\varepsilon }_{t,h}(\delta _\varepsilon )} \frac{\varepsilon }{2}|\nabla _\tau \varphi _\varepsilon |^2 \left( {\tilde{\eta }} \nabla \cdot {\textbf {n}} + \partial _n{\tilde{\eta }}\right) \textrm{d}x. \end{aligned}$$

To this end we use ${\textbf {n}} \cdot \nabla =\sum _{i=1}^d n_i\partial _{x_i}$ and integration by parts for $\partial _{x_i}$. This yields

$$\begin{aligned} -\int _{\Gamma ^{m_\varepsilon }_{t,h}(\delta _\varepsilon )}\varepsilon \nabla _\tau \varphi _\varepsilon \cdot \left( ({\textbf {n}} \cdot \nabla )\nabla \varphi _\varepsilon \right) {\tilde{\eta }}\,\textrm{d}x&=-\frac{1}{2}\int _{\Gamma ^{m_\varepsilon }_{t,h}(\delta _\varepsilon )} \varepsilon ({\textbf {n}} \cdot \nabla )|\nabla _\tau \varphi _\varepsilon |^2 {\tilde{\eta }}\,\textrm{d}x\\&=\int _{\Gamma ^{m_\varepsilon }_{t,h}(\delta _\varepsilon )} \frac{\varepsilon }{2}|\nabla _\tau \varphi _\varepsilon |^2 \nabla \cdot ({\tilde{\eta }}{\textbf {n}} )\,\textrm{d}x, \end{aligned}$$

where we used $({\textbf {n}} \cdot \nabla )(Id -{\textbf {n}} \otimes {\textbf {n}} )=0$ in the second step. Therefore (4.9) holds and this shows Lemma 4.3. $\square$

### Parametrization of the Majority of the Interface

In order for our estimate on the problematic term (4.2f) provided by Lemma 4.4 to work, we need to represent a suitable level set of the phase-field $\varphi _\varepsilon$ as approximately a graph of a small function $h_\varepsilon$ over the surface $\Gamma ^{m_\varepsilon }_t$, as only then the term (4.5c) becomes controllable in terms of a constant $\varepsilon ^2/m_\varepsilon$ and the relative entropy (see Corollary 4.11 below for details).

In this section we construct for a.e. $t\in [0,T_0]$ a 1-Lipschitz-function $h=h_\varepsilon (.,t):\Gamma ^{m_\varepsilon }_t\rightarrow (-\delta ,\delta )$ that is used to approximate a suitable level set of $\varphi _\varepsilon (.,t)$. To this end, we need the following proposition about local interface errors of a BV-set compared to the strong interface $\Gamma _t^{m_\varepsilon }$ from Sect. 2.

#### Proposition 4.5

Let $T_0>0$, $\delta >0$ and $\Gamma ^{m_\varepsilon }$, $\Omega ^{m_\varepsilon ,\pm }$, ${\textbf{v}}^{m_\varepsilon }$ for $m_\varepsilon >0$ small be as in Sect. 2. Moreover, let ${\textbf {n}}$ be the extension of the normal from (2.2) and $\xi$ be defined as in (2.11). Let $t\in [0,T_0]$ be fixed and ${\tilde{\chi }}\in {\text {BV}}({\mathbb {R}}^d;\{0,1\})$ be arbitrary. We set $\chi :=\chi _{\Omega ^{m_\varepsilon ,+}_t}$.

Let $\theta :[0,\infty )\rightarrow [0,1]$ be a smooth cutoff with $\theta \equiv 0$ outside of $[0,\frac{1}{2}]$ and $\theta \equiv 1$ in $[0,\frac{1}{4}]$. We define the local height of the one-sided interface errors $h^\pm _t=h^\pm _{t,\varepsilon }:\Gamma ^{m_\varepsilon }_t\rightarrow [0,\frac{\delta }{2}]$ in $\pm {\textbf {n}}$-direction as

$$\begin{aligned} h^\pm _t(s):= \int _0^\infty (\chi -{\tilde{\chi }})(s+y{\textbf {n}} (s,t)) \, \theta \Big (\frac{y}{\delta }\Big ) \,{\textrm{d}}y\quad \text { for a.e. }s\in \Gamma ^{m_\varepsilon }_t. \end{aligned}$$

Then $h^\pm _t$ are *BV*-functions and we denote the distributional tangential derivative by $D^tan h^\pm _t$, by $\nabla ^tan h^\pm _t$ the density of the absolutely continuous part of $D^tan h^\pm _t$ with respect to ${\mathcal {H}}^{d-1}$ and by $D^sh^\pm _t$ the singular part. Finally, let ${\tilde{G}}_t:=\{s+(h^+_t-h^-_t)(s){\textbf {n}} (s,t) : s\in \Gamma ^{m_\varepsilon }_t\}$ denote the graph of $h^+_t-h^-_t$.

Then we have the following estimates with constants independent of ${\tilde{\chi }}$ and $t\in [0,T_0]$:

4.10 $$\begin{aligned} \int _{\Gamma ^{m_\varepsilon }_t} |h^\pm _t|^2\,d{\mathcal {H}}^{d-1} \le C \int _{{\mathbb {R}}^d}|\chi -{\tilde{\chi }}| \min \left\{ d_{\Gamma ^{m_\varepsilon }_t},1\right\} \,\textrm{d}x \end{aligned}$$

as well as

4.11 $$\begin{aligned}&\int _{\Gamma ^{m_\varepsilon }_t} \min \{|\nabla ^{\tan } h^\pm _t|^2,|\nabla ^{\tan } h^\pm _t|\} \,d{\mathcal {H}}^{d-1} + |D^s h^\pm _t|(\Gamma ^{m_\varepsilon }_t)\nonumber \\&\quad \le C \int _{{\mathbb {R}}^d}{\Big (}1-\xi (.,t)\cdot \frac{\nabla {\tilde{\chi }}}{|\nabla {\tilde{\chi }}|}{\Big )} \,\textrm{d}|\nabla {\tilde{\chi }}|+ C \int _{{\mathbb {R}}^d}|\chi -{\tilde{\chi }}| \min \left\{ d_{\Gamma ^{m_\varepsilon }_t},1\right\} \,\textrm{d}x, \end{aligned}$$

and

4.12 $$\begin{aligned} \int _{{{\mathbb {R}}^d}\setminus {\tilde{G}}_t} 1 \,\textrm{d}|\nabla {\tilde{\chi }}|&\le C \int _{{{\mathbb {R}}^d}} {\Big (}1-\xi (.,t)\cdot \frac{\nabla {\tilde{\chi }}}{|\nabla {\tilde{\chi }}|}{\Big )} \,\textrm{d}|\nabla {\tilde{\chi }}| \nonumber \\  &\quad + C\int _{{\mathbb {R}}^d}|\chi -{\tilde{\chi }}| \min \left\{ d_{\Gamma ^{m_\varepsilon }_t},1\right\} \,\textrm{d}x. \end{aligned}$$

#### Proof

The assertions up to (4.12) can be shown analogously to [16, Proposition 26a–b]. Moreover, (4.12) may be established readily using the arguments for statement c) of [16, Proposition 26]. $\square$

The plan is to apply the previous Proposition 4.5 to a suitable level set of $\varphi _\varepsilon (.,t)$ for a.e. $t\in [0,T_0]$. The latter is selected in the following lemma:

#### Lemma 4.6

Let $t\in [0,T_0]$ be such that $\varphi _\varepsilon (.,t)\in H^1(\Omega )$. Then the relative entropy $E[\varphi _\varepsilon |\Gamma ^{m_\varepsilon }](t)$ from (2.9) is well-defined and finite. Moreover, there exists a level $b(t)=b_\varepsilon (t)\in (-\frac{1}{2},\frac{1}{2})$ such that the corresponding super-level set $S_{b(t)}:=\{x\in \Omega :\varphi _\varepsilon (x,t)>b(t)\}$ is a set of finite perimeter (possibly empty) and satisfies with $\xi$ as in (2.11) and $\psi$ as in (2.6) the estimate

$$\begin{aligned} \int _{{{\mathbb {R}}^d}} {\Big (}1-\xi (.,t) \cdot \frac{\nabla \chi _{S_{b(t)}}}{|\nabla \chi _{S_{b(t)}}|}{\Big )} \,\textrm{d}|\nabla \chi _{S_{b(t)}}| \le \frac{2}{\psi (\frac{1}{2})-\psi (-\frac{1}{2})} E[\varphi _\varepsilon |\Gamma ^{m_\varepsilon }](t). \end{aligned}$$

#### Proof

Let *t* be as in the lemma. Then $E[\varphi _\varepsilon |\Gamma ^{m_\varepsilon }](t)$ is well-defined and finite because of $\varphi _\varepsilon (.,t)\in H^1(\Omega )$. Moreover, $\varphi _\varepsilon (.,t)\in BV(\Omega )$ yields together with the coarea-formula for BV-functions, cf. Ambrosio, Fusco, Pallara [10, Theorem 3.40] that $S_b:=\{x\in \Omega :\varphi _\varepsilon (x,t)>b\}$ is a set of finite perimeter (possibly empty) for a.e. $b\in {\mathbb {R}}$. Moreover, we use the coarea-formula for BV-functions [10, Theorem 3.40] applied to $\psi _\varepsilon (.,t)=\psi (\varphi _\varepsilon (.,t))\in H^1(\Omega )$ (together with an approximation argument by simple functions) and obtain

$$\begin{aligned} E[\varphi _\varepsilon |\Gamma ^{m_\varepsilon }](t)&\ge \int _{\Omega } \left[ |\nabla \psi _\varepsilon | - \xi \cdot \nabla \psi _\varepsilon \right] \!(.,t)\,\textrm{d}x\\&=\int _{{\mathbb {R}}^d} \left[ 1 - \xi \cdot \frac{\nabla \varphi _\varepsilon }{|\nabla \varphi _\varepsilon |}\right] \!(.,t)\, |\nabla \psi _\varepsilon (.,t)|\,\textrm{d}x\\&=\int _{0}^\sigma \int _{{{\mathbb {R}}^d}} \left[ 1 - \xi \cdot \frac{\nabla \varphi _\varepsilon }{|\nabla \varphi _\varepsilon |}\right] \!(.,t) \,\textrm{d}|\nabla \chi _{S_{\psi ^{-1}(r)}}| \,dr. \end{aligned}$$

This shows the claim by a contradiction argument. $\square$

We combine Proposition 4.5 and Lemma 4.6 in the following lemma:

#### Lemma 4.7

Let $t\in [0,T_0]$ be fixed such that $\varphi _\varepsilon (.,t)\in H^1(\Omega )$. Let the level $b(t)=b_\varepsilon (t)\in (-\frac{1}{2},\frac{1}{2})$ and the super-level set $S_{b(t)}$ be as in Lemma 4.6. We set $\chi :=\chi _{\Omega ^{m_\varepsilon ,+}_t}$. Then there is a 1-Lipschitz function $h_t=h_{t,\varepsilon }:\Gamma ^{m_\varepsilon }_t \rightarrow (-\delta ,\delta )$ subject to the estimate

4.13 $$\begin{aligned}&\int _{\Gamma ^{m_\varepsilon }_t} |h_t|^2 + |\nabla ^{\tan } h_t|^2 \,d{\mathcal {H}}^{d-1} \le C E[\varphi _\varepsilon |\Gamma ^{m_\varepsilon }](t) \nonumber \\  &\quad +C \int _{{\mathbb {R}}^d}|\chi -\chi _{S_{b(t)}}| \min \left\{ d_{\Gamma ^{m_\varepsilon }_t},1\right\} \,\textrm{d}x \end{aligned}$$

such that the graph $G_t:=\{s+h_t(s){\textbf {n}} (s,t):s\in \Gamma ^{m_\varepsilon }_t\}$ approximates the reduced boundary of $S_{b(t)}$ in the following sense:

4.14 $$\begin{aligned} \int _{{{\mathbb {R}}^d}\setminus G_t} 1 \,\textrm{d}|\nabla \chi _{S_{b(t)}}| \le C E[\varphi _\varepsilon |\Gamma ^{m_\varepsilon }](t) +C \int _{{\mathbb {R}}^d}|\chi -\chi _{S_{b(t)}}| \min \left\{ d_{\Gamma ^{m_\varepsilon }_t},1\right\} \,\textrm{d}x. \end{aligned}$$

Finally, constants in the estimates (4.13)–(4.14) are independent of $\varphi _\varepsilon$ and *t*.

#### Proof

By Lemma 4.6 it holds ${\tilde{\chi }}:=\chi _{S_{b(t)}}\in BV({\mathbb {R}}^d;\{0,1\})$. Hence by Proposition 4.5 applied for ${\tilde{\chi }}$ there exist BV-functions $h_t^\pm :\Gamma ^{m_\varepsilon }_t\rightarrow [0,\frac{\delta }{2}]$ such that (4.10)–(4.12) hold. We set ${\tilde{h}}_t:=h^+_t-h^-_t$. It remains to modify ${{\tilde{h}}}$ to obtain a 1-Lipschitz function. To do so, we use a standard Lipschitz truncation strategy (see e.g. [9, 15, Section 6.6.3] or [11]): we consider the maximal operator ${\mathcal {M}}$ over $\Gamma ^{m_\varepsilon }_t$, which can be defined as in [36, Chapter I, §8.1 (ii)] since ${\mathcal {H}}^{d-1}\lfloor \Gamma _t^{m_\varepsilon }$ satisfies the doubling condition with respect to the geodesic balls on $\Gamma _t^{m_\varepsilon }$. Then there is some $C_1>0$ such that

$$\begin{aligned} |u(s_1)-u(s_2)|\le &   C_1|s_1-s_2| \left( {\mathcal {M}}|D^tan u|(s_1)+{\mathcal {M}}|D^tan u|(s_2)\right) \quad \\    &   \text {for }{\mathcal {H}}^{d-1}\text {-a.e. }s_1,s_2\in \Gamma ^{m_\varepsilon }_t \end{aligned}$$

for all $u\in BV(\Gamma ^{m_\varepsilon }_t)$ with some $C_1>0$ independent of *u* and small $m_\varepsilon$. More precisely, in the case that $\Gamma ^{m_\varepsilon }_t$ is replaced by ${\mathbb {R}}^{d-1}$ this inequality is shown in [11, Lemma 2(c)]. Then the estimate in the present case can be shown by localization. Moreover, because of the continuous dependence on $t\in [0,T]$ and $m_\varepsilon \in [0, m_0]$ (cf. Theorem 7.7), the constant can be chosen uniformly in $t\in [0,T_0]$, $m_\varepsilon \in [0,m_0]$ for $\varepsilon >0$ sufficiently small. Using the precise representative for ${\tilde{h}}_t$ we define

$$\begin{aligned} h_t(s):={\tilde{h}}_t(s)\quad \text { for all }\quad s\in A_t:=\{s\in \Gamma ^{m_\varepsilon }_t:({\mathcal {M}}|D^tan \tilde{h}_t|)(s)\le {\overline{c}}\}, \end{aligned}$$

where ${\overline{c}}>0$ is a small constant to be determined. The so-defined function *h* is Lipschitz on $A_t$ with Lipschitz-constant bounded by $c_1{\overline{c}}$. We extend *h* to all of $\Gamma ^{m_\varepsilon }_t$ as a Lipschitz function via the standard extension, cf. [10, Proposition 2.12]), i.e. 

$$\begin{aligned} h_t(s):=\inf _{{\tilde{s}}\in A_t}\{{\tilde{h}}_t({\tilde{s}})+Lip ({\tilde{h}}_t)|s-{\tilde{s}}|\}, \end{aligned}$$

where the Lipschitz constant stays the same. Hence for ${\overline{c}}$ small enough (independent of ${\tilde{h}}_t$, $h_t$, *t* and small $m_\varepsilon$), we obtain that $h_t$ is 1-Lipschitz and bounded by $\frac{3\delta }{4}$. Moreover, using the weak $L^1$-estimate for the maximal operator we obtain

4.15 $$\begin{aligned} \begin{aligned} {\mathcal {H}}^{d-1}\left( \Gamma ^{m_\varepsilon }_t\setminus A_t\right)&\le C \int _{\Gamma ^{m_\varepsilon }_t} |\nabla ^tan {\tilde{h}}_t| \,d{\mathcal {H}}^{d-1} + |D^s{\tilde{h}}_t|(\Gamma ^{m_\varepsilon }_t)\\&\le CE[\varphi _\varepsilon |\Gamma ^{m_\varepsilon }](t) +C\int _{{\mathbb {R}}^d}|\chi -{\tilde{\chi }}| \min \left\{ d_{\Gamma ^{m_\varepsilon }_t},1\right\} \,\textrm{d}x,\end{aligned} \end{aligned}$$

where we used (4.11) and Lemma 4.6 in the last step. Because of $|\nabla ^tan h_t|\le C$ as well as by (4.11) and the fact that $D^tan {\tilde{h}}_t=\nabla ^tan {\tilde{h}}_t=\nabla ^tan h_t$ a.e. on $A_t$, this establishes the bound

$$\begin{aligned}&\int _{\Gamma ^{m_\varepsilon }_t} |\nabla ^{\tan } h_t|^2 \,d{\mathcal {H}}^{d-1} \le C E[\varphi _\varepsilon |\Gamma ^{m_\varepsilon }](t) +C\int _{{\mathbb {R}}^d}|\chi -{\tilde{\chi }}| \min \left\{ d_{\Gamma ^{m_\varepsilon }_t},1\right\} \,\textrm{d}x. \end{aligned}$$

Hence the $|h_t|^2$-part of the bound (4.13) is left to prove. The latter follows because of (4.15), the boundedness of $h_t$ and (4.11). Finally, (4.14) follows from (4.12) in Proposition 4.5, the estimate (4.15) and Lemma 4.6. $\square$

#### Corollary 4.8

Let $t\in [0,T_0]$ be fixed such that $\varphi _\varepsilon (.,t)\in H^1(\Omega )$, let $b(t)=b_\varepsilon (t), S_{b(t)}$ be as in Lemma 4.6 and let $h_t=h_{t,\varepsilon }:\Gamma ^{m_\varepsilon }_t\rightarrow (-\delta ,\delta )$ be as in Lemma 4.7. Then, with $\chi :=\chi _{\Omega ^{m_\varepsilon ,+}_t}$, it holds that

4.16 $$\begin{aligned} \begin{aligned}&\int _{\Gamma ^{m_\varepsilon }_t(2\delta )}(|h_t|_{P_{\Gamma ^{m_\varepsilon }}}|^2+|\nabla _\tau (h_t|_{P_{\Gamma ^{m_\varepsilon }}})|^2)\left( \varepsilon |\nabla \varphi _\varepsilon |^2+\frac{1}{\varepsilon }W(\varphi _\varepsilon )\right) |_{(x,t)}\,\textrm{d}x\\&\quad \le C E[\varphi _\varepsilon |\Gamma ^{m_\varepsilon }](t) +C \int _{{\mathbb {R}}^d}|\chi -\chi _{S_{b(t)}}|\min \left\{ d_{\Gamma ^{m_\varepsilon }_t},1\right\} \,\textrm{d}x. \end{aligned} \end{aligned}$$

#### Proof

Using of the identity (2.5) for $\nabla _\tau$, we can exchange $\nabla _\tau (h_t|_{P_{\Gamma ^{m_\varepsilon }}})$ by $(\nabla ^tan h_t)|_{P_{\Gamma ^{m_\varepsilon }}}$ in the above estimate. Moreover, since both $h_t$ and $\nabla ^tan h_t$ are uniformly bounded, we can consider the estimate with $\partial _n\varphi _\varepsilon$ instead of $\nabla \varphi _\varepsilon$, since tangential derivatives are controlled by (2.15). Then we apply an integral transformation with the tubular neighbourhood coordinates to obtain

$$\begin{aligned}&\int _{\Gamma ^{m_\varepsilon }_t(2\delta )}(|h_t|_{P_{\Gamma ^{m_\varepsilon }}}|^2+|(\nabla ^tan h_t)|_{P_{\Gamma ^{m_\varepsilon }}}|^2)\left( \varepsilon |\partial _n\varphi _\varepsilon |^2+\frac{1}{\varepsilon }W(\varphi _\varepsilon )\right) |_{(x,t)}\,\textrm{d}x\\&\quad =\int _{\Gamma ^{m_\varepsilon }_t}(|h_t|^2+|\nabla ^tan h_t|^2)(s)\\&\qquad \int _{-2\delta }^{2\delta }\left( \varepsilon |\partial _r(\varphi _\varepsilon |_{{X_{m_\varepsilon }}})|^2+\frac{1}{\varepsilon }W(\varphi _\varepsilon |_{{X_{m_\varepsilon }}})\right) |_{(r,s,t)}J_t(r,s)\,dr\,ds, \end{aligned}$$

where the factor $J_t=J_t(m_\varepsilon )$ satisfies for some $c_J,C_J>0$ independent of *t* and $m_\varepsilon$

$$\begin{aligned} c_J\le J_t(r,s)\le C_J \qquad \text {for all }s\in \Gamma ^{m_\varepsilon }_t,r\in (-\delta ,\delta ). \end{aligned}$$

Let $\sigma =\psi (1)$ be as in (2.6). For $s\in \Gamma ^{m_\varepsilon }_t$ such that the inner integral with respect to $r\in (-2\delta ,2\delta )$ is less or equal $4\sigma$, the desired estimate follows from Lemma 4.7. For $s\in \Gamma ^{m_\varepsilon }_t$ for which this is not the case, we use that $|\psi (\varphi )|\le \sigma$ for all $\varphi \in [-1,1]$ and therefore

$$\begin{aligned} \left| \int _{-2\delta }^{2\delta }\partial _r\left( \psi (\varphi _\varepsilon |_{{X_{m_\varepsilon }}(r,s,t)})\right) \,dr\right| \le 2\sigma . \end{aligned}$$

Hence it follows for such $s\in \Gamma ^{m_\varepsilon }_t$ that

$$\begin{aligned}&\int _{-2\delta }^{2\delta }\left( \varepsilon |\partial _r(\varphi _\varepsilon |_{{X_{m_\varepsilon }}})|^2+\frac{1}{\varepsilon }W(\varphi _\varepsilon |_{{X_{m_\varepsilon }}})-\partial _r\left( \psi (\varphi _\varepsilon |_{{X_{m_\varepsilon }}})\right) \right) |_{(r,s,t)}J_t(r,s)\,dr\\&\quad \ge c_J\int _{-2\delta }^{2\delta }\left( \varepsilon |\partial _r(\varphi _\varepsilon |_{{X_{m_\varepsilon }}})|^2+\frac{1}{\varepsilon }W(\varphi _\varepsilon |_{{X_{m_\varepsilon }}})-\partial _r\left( \psi (\varphi _\varepsilon |_{{X_{m_\varepsilon }}})\right) \right) |_{(r,s,t)}\,dr\\&\quad \ge \frac{c_J}{2}\int _{-2\delta }^{2\delta }\left( \varepsilon |\partial _r(\varphi _\varepsilon |_{{X_{m_\varepsilon }}})|^2+\frac{1}{\varepsilon }W(\varphi _\varepsilon |_{{X_{m_\varepsilon }}})\right) |_{(r,s,t)}\,dr\\&\quad \ge \frac{c_J}{2C_J}\int _{-2\delta }^{2\delta }\left( \varepsilon |\partial _r(\varphi _\varepsilon |_{{X_{m_\varepsilon }}})|^2+\frac{1}{\varepsilon }W(\varphi _\varepsilon |_{{X_{m_\varepsilon }}})\right) |_{(r,s,t)}J_t(r,s)\,dr. \end{aligned}$$

Therefore the contribution in this case can be estimated with (2.15) and by using that $h_t$ and $\nabla ^tan h_t$ are uniformly bounded. $\square$

Finally, in order to control the second term in (4.16) in the end, we need the following lemma:

#### Lemma 4.9

Let $t\in [0,T_0]$ be fixed such that $\varphi _\varepsilon (.,t)\in H^1(\Omega )$ and let $b(t)=b_\varepsilon (t), S_{b(t)}$ be as in Lemma 4.6. Then with $\chi :=\chi _{\Omega ^{m_\varepsilon ,+}_t}$ and $E_bulk [\varphi _\varepsilon |\Gamma ^{m_\varepsilon }]$ from Sect. 2.2 it follows that

4.17 $$\begin{aligned} \int _{{\mathbb {R}}^d}|\chi -\chi _{S_{b(t)}}|\min \left\{ d_{\Gamma ^{m_\varepsilon }_t},1\right\} \,\textrm{d}x \le CE_bulk [\varphi _\varepsilon |\Gamma ^{m_\varepsilon }](t). \end{aligned}$$

#### Proof

The integrand is zero on ${\mathbb {R}}^d\setminus {\overline{\Omega }}$. Moreover, note that (2.27) yields

$$\begin{aligned} \int _\Omega \left| \sigma \chi -\psi _\varepsilon (.,t)\right| \min \{d_{\Gamma ^{m_\varepsilon }_t},1\}\,\textrm{d}x \le C E_bulk [\varphi _\varepsilon |\Gamma ^{m_\varepsilon }](t). \end{aligned}$$

Hence we obtain

$$\begin{aligned}  &   \int _{\Omega ^{m_\varepsilon ,+}_t}\left| \sigma -\psi _\varepsilon (.,t)\right| \min \{d_{\Gamma ^{m_\varepsilon }_t},1\}\,\textrm{d}x +\int _{\Omega ^{m_\varepsilon ,-}_t}\left| \psi _\varepsilon (.,t)\right| \min \{d_{\Gamma ^{m_\varepsilon }_t},1\}\,\textrm{d}x\\  &   \quad \le C E_bulk [\varphi _\varepsilon |\Gamma ^{m_\varepsilon }](t). \end{aligned}$$

For a.e. $x\in \Omega ^{m_\varepsilon ,+}_t\cap S_{b(t)}$ it holds $|\chi -\chi _{S_{b(t)}}|(x)=0$. For a.e. $x\in \Omega ^{m_\varepsilon ,+}_t \setminus S_{b(t)}$ we have $|\chi -\chi _{S_{b(t)}}|(x)=1$ and $\psi _\varepsilon (x,t)\le \psi (b(t))\le \psi (\frac{1}{2})$. Hence $|\sigma -\psi _\varepsilon (x,t)|\ge |\sigma -\psi (\frac{1}{2})|>0$ since $b(t)\in (-\frac{1}{2},\frac{1}{2})$. This shows the estimate on $\Omega ^{m_\varepsilon ,+}_t$. Moreover, for a.e. $x\in \Omega ^{m_\varepsilon ,-}_t\setminus S_{b(t)}$ we have $|\chi -\chi _{S_{b(t)}}|(x)=0$. Finally, for a.e. $x\in \Omega ^{m_\varepsilon ,-}_t\cap S_{b(t)}$ it holds $|\chi -\chi _{S_{b(t)}}|(x)=1$ and $\psi _\varepsilon (x,t)>\psi (b(t))\ge \psi (-\frac{1}{2})>0$. This yields the estimate on $\Omega ^{m_\varepsilon ,-}_t$. $\square$

### Estimate of Energy Away from Strip Around Level Set

In this section we estimate the remainder terms (4.5b)–(4.5c) from Lemma 4.4 involving the energy density for $\varphi _\varepsilon$. Therefore we show the following lemma:

#### Lemma 4.10

Let $t\in [0,T_0]$ be fixed such that $\varphi _\varepsilon (.,t)\in H^1(\Omega )$ and let $b(t)=b_\varepsilon (t), S_{b(t)}$ be as in Lemma 4.6. We set $\chi =\chi _{\Omega ^{m_\varepsilon ,+}_t}$. Moreover, let $h=h_{t,\varepsilon }:\Gamma ^{m_\varepsilon }_t\rightarrow (-\delta ,\delta )$ be as in Lemma 4.7. We define the shifted tubular neighbourhood $\Gamma ^{m_\varepsilon }_{t,h}({\tilde{\delta }})$ for ${\tilde{\delta }}\in (0,\delta ]$ as in (4.3). For $\kappa >0$ fixed and $\varepsilon >0$ small we obtain with some constants $c,C>0$ independent of $\varphi _\varepsilon$, *h*, *t* and $\kappa$

$$\begin{aligned}&\int _{\Gamma ^{m_\varepsilon }_t(\delta ) \setminus \Gamma ^{m_\varepsilon }_{t,h}(\kappa \varepsilon )} \left( \varepsilon \frac{|\nabla \varphi _\varepsilon |^2}{2} + \frac{ W(\varphi _\varepsilon )}{\varepsilon }\right) \, \textrm{d}x\\&\quad \le C\left( E[\varphi _\varepsilon |\Gamma ^{m_\varepsilon }](t) +\int _{{\mathbb {R}}^d}|\chi -\chi _{S_{b(t)}}|\min \left\{ d_{\Gamma ^{m_\varepsilon }_t},1\right\} \,\textrm{d}x+ e^{-c\kappa }\right) . \end{aligned}$$

#### Proof

Let $G_t$ be the graph of $h=h_{t,\varepsilon }$ as in Lemma 4.7. Then $R_t:=P_{\Gamma ^{m_\varepsilon }_t}(G_t\cap {\text {supp}}|\nabla \chi _{S_{b(t)}}|)\subseteq \Gamma ^{m_\varepsilon }_t$ satisfies

$$\begin{aligned} {\mathcal {H}}^{d-1}(\Gamma ^{m_\varepsilon }_t\setminus R_t)\le C\left( E[\varphi _\varepsilon |\Gamma ^{m_\varepsilon }](t) +\int _{{\mathbb {R}}^d}|\chi -\chi _{S_{b(t)}}|\min \left\{ d_{\Gamma ^{m_\varepsilon }_t},1\right\} \,\textrm{d}x\right) , \end{aligned}$$

where we used that ${\mathcal {H}}^{d-1}((\Gamma ^{m_\varepsilon }_t\setminus R_t)\cap P_{\Gamma ^{m_\varepsilon }_t}({\text {supp}}|\nabla \chi _{S_{b(t)}}|))$ is controlled by (4.14) and that for the remaining part ${\tilde{R}}_t:=(\Gamma ^{m_\varepsilon }_t\setminus R_t)\setminus P_{\Gamma ^{m_\varepsilon }_t}({\text {supp}}|\nabla \chi _{S_{b(t)}}|)$ we have

$$\begin{aligned} {\mathcal {H}}^{d-1}({\tilde{R}}_t)=\int _{{\tilde{R}}_t}\int _{\frac{\delta }{2}}^\delta \frac{2}{\delta }\,dr\,d{\mathcal {H}}^{d-1}(s) \le C\int _{{\mathbb {R}}^d}|\chi -\chi _{S_{b(t)}}|\min \left\{ d_{\Gamma ^{m_\varepsilon }_t},1\right\} \,\textrm{d}x \end{aligned}$$

since $s\in {\tilde{R}}_t$ implies $|\chi -\chi _{S_{b(t)}}|(s+r{\textbf {n}} (s,t))=1$ either for all $r\in (-\delta ,-\frac{\delta }{2})$ or for all $r\in (\frac{\delta }{2},\delta )$.

Moreover, for $s\in R_t$ it holds that

4.18 $$\begin{aligned} \varphi _\varepsilon (s+h(s){\textbf {n}} (s,t),t)=b(t). \end{aligned}$$

The strategy is to use an ODE inequality argument in normal direction using the control of the relative entropy over the equipartition error. More precisely, by (2.15) we have

4.19 $$\begin{aligned} \int _{\Gamma ^{m_\varepsilon }_t} \int _{-\delta }^{\delta } \frac{1}{2}\left| \sqrt{\varepsilon }\partial _r{\tilde{\varphi }}_\varepsilon -\frac{1}{\sqrt{\varepsilon }}\sqrt{2W({\tilde{\varphi }}_\varepsilon )}\right| ^2\!(.,t) J_t(r,s)\,dr \, d{\mathcal {H}}^{d-1}(s)\le E[\varphi _\varepsilon |\Gamma ^{m_\varepsilon }](t), \end{aligned}$$

where ${\tilde{\varphi }}_\varepsilon :=\varphi _\varepsilon |_{X_{m_\varepsilon }(.,t)}$ with $X_{m_\varepsilon }$ as in Sect. 2 and the factor $J_t=J_t(m_\varepsilon )$ appears due to an integral transformation and satisfies for some $c_J,C_J>0$ independent of *t* and $m_\varepsilon$

$$\begin{aligned} c_J\le J_t(r,s)\le C_J \qquad \text {for all }s\in \Gamma ^{m_\varepsilon }_t,r\in (-\delta ,\delta ). \end{aligned}$$

With $f:=\sqrt{\varepsilon }\partial _r{\tilde{\varphi }}_\varepsilon -\frac{1}{\sqrt{\varepsilon }}\sqrt{2W({\tilde{\varphi }}_\varepsilon )}$ it holds that

$$\begin{aligned} \partial _r{\tilde{\varphi }}_\varepsilon = \frac{1}{\varepsilon }\sqrt{2W({\tilde{\varphi }}_\varepsilon )}- \frac{1}{\sqrt{\varepsilon }} f, \end{aligned}$$

where $\sqrt{W(\varphi )}\ge c(1-\varphi )(1+\varphi )\ge c'(1-\varphi )$ if $\varphi \ge -\frac{3}{4}$. Hence if ${\tilde{\varphi }}_\varepsilon \ge -\frac{3}{4}$ we have

$$\begin{aligned} \partial _r (1-{\tilde{\varphi }}_\varepsilon )^2&=2(1-{\tilde{\varphi }}_\varepsilon )(-\partial _r{\tilde{\varphi }}_\varepsilon )\le - \frac{2c'}{\varepsilon }(1-{\tilde{\varphi }}_\varepsilon )^2+ \frac{2}{\sqrt{\varepsilon }} f(1-{\tilde{\varphi }}_\varepsilon )\\&\le - \frac{c'}{\varepsilon }(1-{\tilde{\varphi }}_\varepsilon )^2+ C_1|f|^2. \end{aligned}$$

We multiply the preceding inequality by $e^{-\frac{c}{\varepsilon }(r-h(s))}$ and integrate from *h*(*s*) to *r*. Then we obtain for $s\in R_t$ because of (4.18)

4.20 $$\begin{aligned} (1-{\tilde{\varphi }}_\varepsilon )^2 (s,r) \le e^{-\frac{c}{\varepsilon }(r-h(s))}(1-b(t))^2 + \frac{C_1}{c_J}\int _{-\delta }^{\delta } |f(s,r)|^2J_t(r,s)\, dr \end{aligned}$$

for all $r\in [h(s),\delta )$ provided that ${\tilde{\varphi }}_\varepsilon ({\tilde{r}},s)\ge -\frac{3}{4}$ for all ${\tilde{r}}\in [h(s),r)$. We define

$$\begin{aligned} S_t:= \left\{ s\in R_t: \frac{C_1}{c_J}\int _{-\delta }^{\delta } |f(s,r)|^2J_t(r,s)\, dr\le \frac{1}{4}\right\} . \end{aligned}$$

Then, due to $b(t)\in (-\frac{1}{2},\frac{1}{2})$ and (4.20) we obtain ${\tilde{\varphi }}_\varepsilon (r,s)\ge -\frac{3}{4}$ for all $r\in [h(s),\delta )$ and $s\in S_t$. Analogously one shows ${\tilde{\varphi }}_\varepsilon (r,s)\le \frac{3}{4}$ for all $r\in (-\delta ,h(s)]$ and $s\in S_t$. Moreover, note that because of (4.19) and the definition of *f* we have

$$\begin{aligned} {\mathcal {H}}^{d-1}(R_t\setminus S_t)\le \frac{4c_J}{C_1} \int _{R_t} \int _{-\delta }^{\delta } |f(s,r)|^2J_t(r,s)\, dr \,d{\mathcal {H}}^{d-1}(s)\le \frac{8c_J}{C_1} E[\varphi _\varepsilon |\Gamma ^{m_\varepsilon }](t). \end{aligned}$$

We use an integral transformation to obtain

$$\begin{aligned}&\int _{\Gamma ^{m_\varepsilon }_t(\delta )\setminus \Gamma ^{m_\varepsilon }_{t,h}(\kappa \varepsilon )} \left( \frac{\varepsilon }{2}|\nabla \varphi _\varepsilon |^2 + \frac{W(\varphi _\varepsilon )}{\varepsilon }\right) \!\textrm{d}x\\&\quad = \int _{\Gamma ^{m_\varepsilon }_t} \int _{h(s)+\kappa \varepsilon }^\delta \left( \frac{\varepsilon }{2}|\nabla \varphi _\varepsilon |^2|_{X_{m_\varepsilon }} + \frac{W({\tilde{\varphi }}_\varepsilon )}{\varepsilon }\right) \!J_t(r,s)\,dr\, \, d{\mathcal {H}}^{d-1}(s)\\&\qquad +\int _{\Gamma ^{m_\varepsilon }_t} \int _{-\delta }^{h(s)-\kappa \varepsilon }\left( \frac{\varepsilon }{2}|\nabla \varphi _\varepsilon |^2|_{X_{m_\varepsilon }} + \frac{W({\tilde{\varphi }}_\varepsilon )}{\varepsilon }\right) \!J_t(r,s)\,dr\, \, d{\mathcal {H}}^{d-1}(s). \end{aligned}$$

We use the definition (2.9) to derive an estimate. First, for the contribution over $\Gamma ^{m_\varepsilon }_t\setminus S_t$ we use an integral transformation as above for the last term in (2.9). Since $\xi$ points in normal direction by definition (2.11), the inner integral is uniformly bounded because $|\psi |\le \sigma$. Hence the estimates for ${\mathcal {H}}^{d-1}(\Gamma ^{m_\varepsilon }_t\setminus R_t)$ and ${\mathcal {H}}^{d-1}(R_t\setminus S_t)$ yield that the contribution over $\Gamma ^{m_\varepsilon }_t\setminus S_t$ is suitably estimated by the right hand side of the estimate in the Lemma. For the remaining part over $S_t$, we note that, due to integration by parts and since $\xi$ points in normal direction by definition (2.11), we have

$$\begin{aligned}&\left| \int _{S_t} \int _{h(s)+\kappa \varepsilon }^{\delta }\xi |_{X_{m_\varepsilon }}\cdot \nabla (\psi _\varepsilon |_{X_{m_\varepsilon }}-\sigma )J_t(r,s)\,dr\, \, {\mathcal {H}}^{d-1}(s)\right| \\&\quad \le C\left| \int _{S_t} \int _{h(s)+\kappa \varepsilon }^{\delta }(\psi ({\tilde{\varphi }}_\varepsilon )-\sigma )\,dr\, \, d{\mathcal {H}}^{d-1}(s)\right| \\&\qquad + \left| \int _{S_t}\left. (\psi ({\tilde{\varphi }}_\varepsilon )-\sigma )J_t(r,s)\right| _{r=h(s)+k\varepsilon }^{\delta }\, \, d{\mathcal {H}}^{d-1}(s)\right| \\&\quad \le C\left( e^{-c\kappa }+ \Vert f\Vert _{L^2(\Gamma ^{m_\varepsilon }_t(\delta ))}^2\right) \le C\left( e^{-c\kappa }+ E[\varphi _\varepsilon |\Gamma ^{m_\varepsilon }](t)\right) , \end{aligned}$$

where we have used

$$\begin{aligned} |\psi ({\tilde{\varphi }}_\varepsilon (r,s))-\sigma |  &   \le C \left( e^{-\frac{c}{\varepsilon }(r-h(s))} +\Vert f(s,.)\Vert ^2_{L^2_{J_t(.,s)}(-\delta ,\delta )}\right) \qquad \\    &   \quad \text {for all }r\in [h(s),\delta ), s\in S_t \end{aligned}$$

due to (4.20) and $|\psi (r)-\sigma |\le C (1-r)^2$ for all $r\in {\mathbb {R}}$. Analogously, one shows

$$\begin{aligned}&\left| \int _{S_t} \int ^{h(s)-\kappa \varepsilon }_{-\delta }\xi |_{X_{m_\varepsilon }}\cdot \nabla \psi _\varepsilon |_{X_{m_\varepsilon }} J_t(r,s)\,dr\, \, {\mathcal {H}}^{d-1}(s)\right| \le C\left( e^{-c\kappa }+ E[\varphi _\varepsilon |\Gamma ^{m_\varepsilon }](t)\right) . \end{aligned}$$

Finally, we have $\left| \int _{\Gamma ^{m_\varepsilon }_t\setminus S_t}\int _{(-\delta ,h(s)-\kappa \varepsilon )\cup (h(s)+\kappa \varepsilon ,\delta )} \xi \cdot \nabla \psi _\varepsilon \,\textrm{d}x\right| \le C{\mathcal {H}}^{d-1}(\Gamma ^{m_\varepsilon }_t\setminus S_t)$. Combining these bounds, this shows the claim. $\square$

As a corollary of Lemma 4.10, we estimate the remaining term (4.5c).

#### Corollary 4.11

Let the assumptions and notation of Lemma 4.10 be in place and let $C_0\ge 1$. Moreover, let $C_1>0$ such that $\int _{\Omega }\varepsilon \frac{|\nabla \varphi _\varepsilon |^2}{2} + \frac{W(\varphi _\varepsilon )}{\varepsilon }\,\textrm{d}x\le C_1$. Then for all $\varepsilon$ small with $\delta _\varepsilon :=C_0|\log \varepsilon |\varepsilon \le \delta$ and for some uniform $C>0$ we have

$$\begin{aligned}&\int _{\Gamma ^{m_\varepsilon }_{t,h}(\delta _\varepsilon )} \frac{1}{m_\varepsilon } \left( \frac{\varepsilon }{2}|\nabla \varphi _\varepsilon |^2+\frac{1}{\varepsilon }W(\varphi _\varepsilon )\right) \!(x,t) \left| d_{\Gamma ^{m_\varepsilon }_t}-h(P_{\Gamma ^{m_\varepsilon }_t})\right| ^2\,\textrm{d}x\\&\quad \le CC_0^3|\log \varepsilon |^3 \frac{\varepsilon ^2}{m_\varepsilon }\left( E[\varphi _\varepsilon |\Gamma ^{m_\varepsilon }](t) +\int _{{\mathbb {R}}^d}|\chi -\chi _{S_{b(t)}}|\min \left\{ d_{\Gamma ^{m_\varepsilon }_t},1\right\} \,\textrm{d}x\right) +CC_1\frac{\varepsilon ^2}{m_\varepsilon }. \end{aligned}$$

#### Proof

Let $C_0\ge 1$ be fixed, then for all $\varepsilon$ sufficiently small it holds $2C_0|\log \varepsilon |\varepsilon \le \delta$. We fix $\varepsilon$ small. Let $N\in {\mathbb {N}}$ be such that $C_0|\log \varepsilon |\le N\le 2C_0|\log \varepsilon |$. Then $\delta _\varepsilon \le N\varepsilon \le \delta$ and

$$\begin{aligned}&\int _{\Gamma ^{m_\varepsilon }_{t,h}(N\varepsilon )}\frac{1}{m_\varepsilon } \left( \varepsilon \frac{|\nabla \varphi _\varepsilon |^2}{2} + \frac{W(\varphi _\varepsilon )}{\varepsilon }\right) \!(x,t)\left| d_{\Gamma ^{m_\varepsilon }_t}-h(P_{\Gamma ^{m_\varepsilon }_t})\right| ^2\, \textrm{d}x\\&\quad \le \sum _{n=2}^N C\int _{\Gamma ^{m_\varepsilon }_{t,h}(n\varepsilon )\setminus \Gamma ^{m_\varepsilon }_{t,h}((n-1)\varepsilon )}\frac{1}{m_\varepsilon } \left( \varepsilon \frac{|\nabla \varphi _\varepsilon |^2}{2} + \frac{W(\varphi _\varepsilon )}{\varepsilon }\right) \!(x,t)\left| d_{\Gamma ^{m_\varepsilon }_t}-h(P_{\Gamma ^{m_\varepsilon }_t})\right| ^2\, \textrm{d}x\\&\qquad + C\int _{\Gamma ^{m_\varepsilon }_{t,h}(\varepsilon )}\frac{1}{m_\varepsilon } \left( \varepsilon \frac{|\nabla \varphi _\varepsilon |^2}{2} + \frac{W(\varphi _\varepsilon )}{\varepsilon }\right) \!(x,t)\left| d_{\Gamma ^{m_\varepsilon }_t}-h(P_{\Gamma ^{m_\varepsilon }_t})\right| ^2\, \textrm{d}x\\&\quad \le C\sum _{n=2}^N \frac{\varepsilon ^2}{m_\varepsilon }n^2 \int _{\Gamma ^{m_\varepsilon }_{t,h}(n\varepsilon )\setminus \Gamma ^{m_\varepsilon }_t((n-1)\varepsilon )} \left( \varepsilon \frac{|\nabla \varphi _\varepsilon |^2}{2} + \frac{W(\varphi _\varepsilon )}{\varepsilon }\right) \!(x,t)\, \textrm{d}x+ CC_1\frac{\varepsilon ^2}{m_\varepsilon }\\&\quad \le C\sum _{n=2}^N \frac{\varepsilon ^2}{m_\varepsilon }n^2 \left( E[\varphi _\varepsilon |\Gamma ^{m_\varepsilon }](t) +\int _{{\mathbb {R}}^d}|\chi -\chi _{S_{b(t)}}|\min \left\{ d_{\Gamma ^{m_\varepsilon }_t},1\right\} \,\textrm{d}x + e^{-c(n-1)}\right) \\&\qquad + CC_1\frac{\varepsilon ^2}{m_\varepsilon }\\&\quad \le C \frac{\varepsilon ^2}{m_\varepsilon }N^3 \left( E[\varphi _\varepsilon |\Gamma ^{m_\varepsilon }](t) +\int _{{\mathbb {R}}^d}|\chi -\chi _{S_{b(t)}}|\min \left\{ d_{\Gamma ^{m_\varepsilon }_t},1\right\} \,\textrm{d}x\right) + CC_1 \frac{\varepsilon ^2}{m_\varepsilon }, \end{aligned}$$

where we used Lemma 4.10. This shows the claim. $\square$

## Bulk Error Identity

For the bulk error functional $E_bulk [\varphi _\varepsilon |\Gamma ^{m_\varepsilon }]$ defined in (2.26) we have the following identity:

### Lemma 5.1

(Bulk Error Identity) Let the assumptions of Sect. 2 hold. More precisely, let $({\textbf{v}}_{m_\varepsilon }^\pm ,p_{m_\varepsilon }^\pm ,(\Gamma _t^{m_\varepsilon })_{t\in [0,T_0]})$ for $m_\varepsilon >0$ small be solutions of the adjusted two-phase Navier–Stokes equation (7.1)–(7.7) on $[0,T_0]$, cf. Theorem 7.9 below. Moreover, let $({\textbf{v}}_\varepsilon , \varphi _\varepsilon )$ for $\varepsilon >0$ small be energy dissipating weak solutions to the Navier–Stokes/Allen–Cahn system (1.1a)–(1.1e) on $[0,T_0]$ with constant mobility $m_\varepsilon >0$ as in Remark 1.2. Finally, let $\Gamma ^{m_\varepsilon }, \Omega ^{m_\varepsilon ,\pm }, {\textbf{v}}^{m_\varepsilon }$ be as in (2.1), $\sigma$, $\psi _\varepsilon$, ${\textbf {n}} _\varepsilon$ be as in (2.6)–(2.7), $\xi$, *B* be as in (2.11), $E_bulk [\varphi _\varepsilon |\Gamma ^{m_\varepsilon }]$ and $\vartheta$ be as in (2.26) and $H_\varepsilon$ be defined as in (4.1). Then, for all $T\in [0,T_0]$,

5.1a $$\begin{aligned}&E_bulk [\varphi _\varepsilon |\Gamma ^{m_\varepsilon }](T)\nonumber \\&\quad =E_bulk [\varphi _\varepsilon |\Gamma ^{m_\varepsilon }](0)+\int _0^T\int _\Omega (\sigma \chi _{\Omega ^{m_\varepsilon ,+}} -\psi _\varepsilon )(\partial _t+B\cdot \nabla )\vartheta \,\textrm{d}x\,\textrm{d}t \end{aligned}$$

5.1b $$\begin{aligned}&\qquad +\int _0^T\int _\Omega (\sigma \chi _{\Omega ^{m_\varepsilon ,+}} -\psi _\varepsilon )\vartheta \,\nabla \cdot B\,\textrm{d}x\,\textrm{d}t \end{aligned}$$

5.1c $$\begin{aligned}&\qquad -\int _0^T\int _\Omega \vartheta (B-{\textbf{v}}^{m_\varepsilon })\cdot ({\textbf {n}} _\varepsilon -\xi )|\nabla \psi _\varepsilon |\,\textrm{d}x\,\textrm{d}t \end{aligned}$$

5.1d $$\begin{aligned}&\qquad +\int _0^T\int _\Omega (\sigma \chi _{\Omega ^{m_\varepsilon ,+}} -\psi _\varepsilon )({\textbf{v}}_\varepsilon -{\textbf{v}}^{m_\varepsilon })\cdot \nabla \vartheta \,\textrm{d}x\,\textrm{d}t \end{aligned}$$

5.1e $$\begin{aligned}&\qquad -\int _0^T\int _\Omega \vartheta (B-{\textbf{v}}^{m_\varepsilon })\cdot \xi (|\nabla \psi _\varepsilon |-\varepsilon |\nabla \varphi _\varepsilon |^2)\,\textrm{d}x\,\textrm{d}t \end{aligned}$$

5.1f $$\begin{aligned}&\qquad +m_\varepsilon \int _0^T\int _\Omega \frac{1}{\sqrt{\varepsilon }}\left( H_\varepsilon -\frac{B-{\textbf{v}}^{m_\varepsilon }}{m_\varepsilon }\cdot \xi \,\varepsilon |\nabla \varphi _\varepsilon |\right) \vartheta \sqrt{\varepsilon }|\nabla \varphi _\varepsilon |\,\textrm{d}x\,\textrm{d}t \end{aligned}$$

5.1g $$\begin{aligned}&\qquad -m_\varepsilon \int _0^T\int _\Omega \vartheta \frac{1}{\sqrt{\varepsilon }}\left( H_\varepsilon +\sqrt{2W(\varphi _\varepsilon )}\nabla \cdot \xi \right) \left( \sqrt{\varepsilon }|\nabla \varphi _\varepsilon |-\frac{\sqrt{2W(\varphi _\varepsilon )}}{\sqrt{\varepsilon }}\right) \,\textrm{d}x\,\textrm{d}t \end{aligned}$$

5.1h $$\begin{aligned}&\qquad -m_\varepsilon \int _0^T\int _\Omega \vartheta (\nabla \cdot \xi )\left| \sqrt{\varepsilon }|\nabla \varphi _\varepsilon |-\frac{\sqrt{2W(\varphi _\varepsilon )}}{\sqrt{\varepsilon }}\right| ^2\,\textrm{d}x\,\textrm{d}t \end{aligned}$$

5.1i $$\begin{aligned}&\qquad +m_\varepsilon \int _0^T\int _\Omega \vartheta \sqrt{\varepsilon }|\nabla \varphi _\varepsilon |(\nabla \cdot \xi )\left( \sqrt{\varepsilon }|\nabla \varphi _\varepsilon |-\frac{\sqrt{2W(\varphi _\varepsilon )}}{\sqrt{\varepsilon }}\right) \,\textrm{d}x\,\textrm{d}t. \end{aligned}$$

### Proof

This can be shown analogously to [22, Lemma 7]. The $m_\varepsilon$-factors appear here because of the Allen–Cahn part (1.1c) through the equation for $\partial _t\psi _\varepsilon$. Note that we use a different sign convention for $\vartheta$ compared to [22], this just changes the signs in all terms. $\square$

## Proof of Theorem 3.1(Stability Estimate)

### Proof of Theorem 3.1

Up to (4.2c), (4.2d), (4.2e) and (4.2f) the terms in the estimate for the relative entropy from Lemma 4.1 can be estimated analogously to [22, Proof of Theorem 1]. Here note that $\Vert \nabla {\textbf{v}}_\varepsilon -\nabla {\textbf{v}}^{m_\varepsilon }\Vert _{L^2(0,T;L^2(\Omega ))}^2$ and (4.2a)–(4.2b) are terms with a good sign. At this point, let us estimate a few terms for the convenience of the reader. For example,

$$\begin{aligned}  &   \left| \int _0^T \int _\Omega (\sigma \chi _{\Omega ^{m_\varepsilon ,+}}-\psi _\varepsilon ) (({\textbf{v}}_\varepsilon -{\textbf{v}}^{m_\varepsilon })\cdot \nabla )(\nabla \cdot \xi )\,\textrm{d}x\,\textrm{d}t\right| \\  &   \quad \le C\int _0^T \int _\Omega |\sigma \chi _{\Omega ^{m_\varepsilon ,+}}-\psi _\varepsilon | |{\textbf{v}}_\varepsilon -{\textbf{v}}^{m_\varepsilon }|\,\textrm{d}x\,\textrm{d}t, \end{aligned}$$

where the latter term can be estimated with (2.28) by using $\frac{1}{2}\Vert \nabla {\textbf{v}}_\varepsilon -\nabla {\textbf{v}}^{m_\varepsilon }\Vert _{L^2(0,T;L^2(\Omega ))}^2$ for absorption. Moreover, due to (2.21) it holds that

$$\begin{aligned} \left| \int _0^T \int _\Omega \left( (\partial _t+B\cdot \nabla )|\xi |^2\right) |\nabla \psi _\varepsilon |\,\textrm{d}x\,\textrm{d}t\right| \le \int _0^T \int _\Omega \min \{d_{\Gamma ^{m_\varepsilon }}^2,1\}|\nabla \psi _\varepsilon |\,\textrm{d}x\,\textrm{d}t, \end{aligned}$$

which is controlled due to (2.16). Finally, let us estimate

$$\begin{aligned}  &   \left| \int _0^T \int _\Omega ({\textbf {n}} _\varepsilon \otimes {\textbf {n}} _\varepsilon -\xi \otimes \xi ):\nabla B(\varepsilon |\nabla \varphi _\varepsilon |^2-|\nabla \psi _\varepsilon |)\,\textrm{d}x\,\textrm{d}t\right| \\  &   \quad \le C\int _0^T \int _\Omega \sqrt{1-{\textbf {n}} _\varepsilon \cdot \xi }\left| \varepsilon |\nabla \varphi _\varepsilon |^2-|\nabla \psi _\varepsilon |\right| \,\textrm{d}x\,\textrm{d}t, \end{aligned}$$

where we used $|({\textbf {n}} _\varepsilon \otimes {\textbf {n}} _\varepsilon -\xi \otimes \xi ):\nabla B|=|{\textbf {n}} _\varepsilon \otimes ({\textbf {n}} _\varepsilon -\xi ):\nabla B+(\xi \cdot \nabla )B\cdot ({\textbf {n}} _\varepsilon -\xi )|\le |{\textbf {n}} _\varepsilon -\xi |$ and $|{\textbf {n}} _\varepsilon -\xi |^2=2(1-{\textbf {n}} _\varepsilon \cdot \xi )$. Hence the above term is controlled via (2.17).

Let us consider the remaining four terms (4.2c), (4.2d), (4.2e) and (4.2f) for which new estimates are needed. First, the new choice (2.11) of $\xi$ and *B* compared to [22, (75)–(76)] yields (2.20) and enables us to estimate (4.2c) with (2.16). Moreover, for the estimate of (4.2d) it remains to control

$$\begin{aligned} \int _0^T\int _\Omega \frac{1}{m_\varepsilon }\left| ({\textbf{v}}^{m_\varepsilon }-B)\cdot ({\textbf {n}} _\varepsilon -\xi )\right| ^2 \varepsilon |\nabla \varphi _\varepsilon |^2\,\textrm{d}x\,\textrm{d}t \end{aligned}$$

due to absorption with (4.2a). However, we have ${\textbf{v}}^{m_\varepsilon }-B=-m_\varepsilon H{\textbf {n}} {\tilde{\eta }}_{m_\varepsilon }$ by definition (2.11) of *B*, hence the term is controlled by $m_\varepsilon \int _0^TE[{\textbf{v}}_\varepsilon ,\varphi _\varepsilon | {\textbf{v}}^{m_\varepsilon },\Gamma ^{m_\varepsilon }](t)\,dt$ because of (2.16). Additionally, to estimate the term (4.2e) we write $(\xi \otimes \xi (\nabla B)^\top \xi )\cdot ({\textbf {n}} _\varepsilon -\xi )=(\xi ^\top (\nabla B)^\top \xi )\xi \cdot ({\textbf {n}} _\varepsilon -\xi )$. Hence because of $\xi \cdot ({\textbf {n}} _\varepsilon -\xi )={\textbf {n}} _\varepsilon \cdot \xi -1+1-|\xi |^2$ and (2.10), the term (4.2e) is controlled via (2.16). Finally, we estimate (4.2f) with Lemma 4.4, where the 1-Lipschitz-function $h=h_\varepsilon (.,t)$ for a.e. $t\in [0,T_0]$ is taken from Lemma 4.7 and we consider $\delta _\varepsilon :=C_0|\log \varepsilon |\varepsilon$ for some $C_0\ge 1$ and $\varepsilon$ small. It remains to estimate (4.5b)–(4.5e). First, note that (4.5d) is absorbed by (4.2a). Moreover, (4.5e) is controlled via Corollary 4.8 and Lemma 4.9. More precisely, we have

$$\begin{aligned}&\int _{\Gamma ^{m_\varepsilon }_{t,h}(\delta _\varepsilon )}(|h|_{P_{\Gamma ^{m_\varepsilon }}}|^2+|\nabla _\tau (h|_{P_{\Gamma ^{m_\varepsilon }}})|^2)\left( \varepsilon |\nabla \varphi _\varepsilon |^2+\frac{1}{\varepsilon }W(\varphi _\varepsilon )\right) |_{(x,t)}\,\textrm{d}x\\&\quad \le C E[\varphi _\varepsilon |\Gamma ^{m_\varepsilon }](t) +C \int _{{\mathbb {R}}^d}|\chi -\chi _{S_{b(t)}}|\min \left\{ d_{\Gamma ^{m_\varepsilon }_t},1\right\} \,\textrm{d}x\\&\quad \le C(E[\varphi _\varepsilon |\Gamma ^{m_\varepsilon }](t)+E_bulk [\varphi _\varepsilon |\Gamma ^{m_\varepsilon }](t)), \end{aligned}$$

where $b(t)=b_\varepsilon (t)$ and $S_{b(t)}$ are as in Lemma 4.6. Moreover, (4.5b) is suitably estimated by Lemma 4.10 provided that $C_0\ge 1$ is large enough such that $e^{-cC_0|\log \varepsilon |}\le \varepsilon ^2$. Finally, (4.5c) is controlled by Corollary 4.11 and Lemma 4.9. Altogether, we obtain that

$$\begin{aligned}&\frac{1}{2}\Vert \nabla {\textbf{v}}_\varepsilon -\nabla {\textbf{v}}^{m_\varepsilon }\Vert _{L^2(0,T;L^2(\Omega ))}^2+E[{\textbf{v}}_\varepsilon ,\varphi _\varepsilon | {\textbf{v}}^{m_\varepsilon },\Gamma ^{m_\varepsilon }](T) \\&\quad \le E[{\textbf{v}}_\varepsilon ,\varphi _\varepsilon | {\textbf{v}}^{m_\varepsilon },\Gamma ^{m_\varepsilon }](0) +C\frac{\varepsilon ^2}{m_\varepsilon }\\&\qquad +C\left( \frac{|\log \varepsilon |^3\varepsilon ^2}{m_\varepsilon }+1\right) \int _0^T E[{\textbf{v}}_\varepsilon ,\varphi _\varepsilon | {\textbf{v}}^{m_\varepsilon },\Gamma ^{m_\varepsilon }](t)+E_bulk [\varphi _\varepsilon |\Gamma ^{m_\varepsilon }](t)\,dt. \end{aligned}$$

Next, we estimate the terms on the right hand side in the identity for $E_bulk [\varphi _\varepsilon |\Gamma ^{m_\varepsilon }]$ from Lemma 5.1. The terms (5.1a)–(5.1b) are controlled by the bulk error itself due to (2.32), (2.30) and (2.27). Moreover, (5.1c) and (5.1e) can be estimated by the relative entropy because of (2.16) and (2.17), respectively. The term (5.1d) is controlled by the bulk functional due to (2.28). Moreover, we estimate (5.1f)–(5.1g) via Young’s inequality in order to absorb one part with the positive terms (4.2b) and (4.2a), respectively. The remainders as well as (5.1h)–(5.1i) are controlled by the relative entropy because of (2.16) and (2.14). Finally, this yields

$$\begin{aligned} E_bulk [\varphi _\varepsilon |\Gamma ^{m_\varepsilon }](t)&\le E_bulk [\varphi _\varepsilon |\Gamma ^{m_\varepsilon }](0) \\&\quad +C\int _0^T E[{\textbf{v}}_\varepsilon ,\varphi _\varepsilon | {\textbf{v}}^{m_\varepsilon },\Gamma ^{m_\varepsilon }](t)+E_bulk [\varphi _\varepsilon |\Gamma ^{m_\varepsilon }](t)\,dt. \end{aligned}$$

Let $m_\varepsilon =m_0\varepsilon ^{\beta }$ with $m_0>0$ and $\beta \in (0,2)$ be as in the theorem. Then it holds $\frac{|\log \varepsilon |^3\varepsilon ^2}{m_\varepsilon }\le \frac{C_\beta }{m_0}$ for some constant $C_\beta >0$ independent of $\varepsilon$ for $\varepsilon >0$ small. Finally, the Gronwall inequality yields Theorem 3.1. $\square$

## Existence for the Approximate Two-Phase Flow

In this section we consider the existence of strong solutions for the modified two-phase flow

7.1 $$\begin{aligned} \partial _t {\textbf{v}}_m^\pm +{\textbf{v}}_m^\pm \cdot \nabla {\textbf{v}}_m^\pm -\Delta {\textbf{v}}_m^\pm +\nabla p_m^\pm&= 0&\quad&\text {in }\Omega ^{m,\pm }_t, t\in [0,T_0], \end{aligned}$$

7.2 $$\begin{aligned} {\text {div}}{\textbf{v}}_m^\pm&= 0&\quad&\text {in }\Omega ^{m,\pm }_t, t\in [0,T_0],\end{aligned}$$

7.3 $$\begin{aligned} -\llbracket 2 D{\textbf{v}}_m^\pm -p_m^\pm {\textbf{I}}\rrbracket {\textbf{n}}_{\Gamma ^m_t}&= \sigma H_{\Gamma ^m_t}{\textbf{n}}_{\Gamma ^m_t}  &   \text {on }\Gamma ^m_t, t\in [0,T_0],\end{aligned}$$

7.4 $$\begin{aligned} \llbracket {\textbf{v}}_m^\pm \rrbracket&=0  &   \text {on }\Gamma ^m_t, t\in [0,T_0],\end{aligned}$$

7.5 $$\begin{aligned} V_{\Gamma ^m_t}-{\textbf{n}}_{\Gamma ^m_t}\cdot {\textbf{v}}_m^\pm&= m H_{\Gamma ^m_t}  &   \text {on }\Gamma ^m_t, t\in [0,T_0],\end{aligned}$$

7.6 $$\begin{aligned} {\textbf{v}}_m^-|_{\partial \Omega }&= 0  &   \text {on }\partial \Omega \times (0,T_0),\end{aligned}$$

7.7 $$\begin{aligned} \Gamma ^m_0=\Gamma ^0,\quad {\textbf{v}}_m^\pm |_{t=0}&= {\textbf{v}}_0^\pm  &   \text {in }\Omega ^\pm _0, \end{aligned}$$

where $T_0>0$ is such that (1.2a)–(1.2g) possesses a smooth solution and $m>0$ is sufficiently small. Here, analogously as for $\Omega ^\pm _t$ and $\Gamma _t$, the domain $\Omega$ is the disjoint union of two sufficiently smooth domains $\Omega ^{m,\pm }_t$ and an evolving interface $\Gamma ^m_t=\partial \Omega ^{m,+}_t$ for every $t\in [0,T]$. Furthermore, ${\textbf{n}}_{\Gamma ^m_t}$, $V_{\Gamma ^m_t}$, and $H_{\Gamma ^m_t}$ denote interior normal (with respect to $\Omega ^{m,+}_t$), the normal velocity, and the mean curvature of $\Gamma ^m_t$, respectively. We note that before $m=m_\varepsilon >0$ depends on $\varepsilon >0$ and $m_\varepsilon \rightarrow _{\varepsilon \rightarrow 0} 0$. But in the system above only the value of $m>0$ enters. Therefore we skip the $\varepsilon$-dependence. The goal is to show that then for every $m>0$ sufficiently small also (7.1)–(7.7) possesses a strong solution on the same time interval $[0,T_0]$, which is close to the solution of (1.2a)–(1.2g) in a certain sense. The idea for the proof is to use the interface $\Gamma _t$ of the solution of (1.2a)–(1.2g) for every $t\in [0,T_0]$ as a reference surface and to transform the modified system (7.1)–(7.7) with the aid of the Hanzawa transformation with respect to $\Gamma _t$ to a perturbed two-phase flow problem in $\Omega ^\pm _t$, $t\in [0,T_0]$. To show solvability of the transformed system for small $m>0$ we reduce the system to a fixed-point problem with the aid of the invertibility of the principal part of the linearized system and apply the contraction mapping principle as usual. But to apply this strategy invertibility of the principal part of the linearized system to (7.1)–(7.7) together with uniform estimates in $m>0$ are essential. These results are obtained in the next subsection.

Throughout this section we will use the notation from [35]. In particular, we note that for a Banach space *X*, $1\le p\le \infty$, and $T_0>0$

### Analysis of the Linearized System

In this subsection $(\Gamma _t)_{t\in [0,T_0]}$ is a smooth evolving family of $(d-1)$-dimensional submanifolds such that $\Gamma _t= \partial \Omega ^+_t \subseteq \Omega$ and $\Omega$ is the disjoint union of $\Gamma _t$ and two smooth domains $\Omega ^+_t$ and $\Omega ^-_t$ for every $t\in [0,T_0]$. Moreover, we assume that there is a continuous $X:{\overline{\Omega }}\times [0,T_0]\rightarrow {\overline{\Omega }}$ such that $(X|_{\Omega _0^\pm \times [0,T_0]},{\text {id}}):\overline{\Omega _0^\pm } \times [0,T_0] \rightarrow \overline{\Omega ^\pm }$ are smooth and

1. $X_t:=X(\cdot ,t):\overline{\Omega _0^\pm }\rightarrow \overline{\Omega _t^\pm }$ are smooth diffeomorphisms for all $t\in [0,T_0]$,
2. $$\det (D_\xi X_t(\xi ))=1$$ for all $$\xi \in \Omega _0\setminus \Gamma _0$$, $$t\in [0,T_0]$$.

Here we use the same notation as in Sect. 2. In particular, we have $X_t(\Gamma _0)= \Gamma _t$ for all $t\in [0,T_0]$. In the later applications $(\Gamma _t)_{t\in [0,T_0]}$ is given by the smooth solution of (1.2a)–(1.2g) and $X_t=X(\cdot ,t)$ can be obtained as the solutions of

$$\begin{aligned} \begin{array}{cl} \frac{d}{dt} X_t(\xi ) = {\textbf{v}}_0^\pm (X_t(\xi ),t) & \quad \text {for all }\xi \in \overline{\Omega ^\pm _0}, t\in [0,T_0],\\ X_0(\xi ) = \xi & \quad \text {for all }\xi \in \overline{\Omega ^\pm _0}. \end{array} \end{aligned}$$

Since $\llbracket {\textbf{v}}^\pm _0 \rrbracket =0$, $X:\overline{\Omega _0}\times [0,T_0]\rightarrow \overline{\Omega _0}$ is well defined and continuous.

We consider the linearized system

7.8 $$\begin{aligned} \partial _t {\textbf{v}}^\pm -\Delta {\textbf{v}}^\pm +\nabla p^\pm&= {\textbf{f}}&\qquad&\text {in }\Omega ^\pm _t, t\in [0,T_0], \end{aligned}$$

7.9 $$\begin{aligned} {\text {div}}{\textbf{v}}^\pm&= g&\qquad&\text {in }\Omega ^\pm _t, t\in [0,T_0],\end{aligned}$$

7.10 $$\begin{aligned} -\llbracket 2D{\textbf{v}}^\pm -p^\pm {\textbf{I}}\rrbracket {\textbf{n}}_{\Gamma _t}&= \sigma \Delta _{\Gamma _t} h {\textbf{n}}_{\Gamma _t} + {\textbf{a}}  &   \text {on }\Gamma _t, t\in [0,T_0],\end{aligned}$$

7.11 $$\begin{aligned} \llbracket {\textbf{v}}^\pm \rrbracket&=0  &   \text {on }\Gamma _t, t\in [0,T_0],\end{aligned}$$

7.12 $$\begin{aligned} \partial _t^\bullet h&= {\textbf{n}}_{\Gamma _t} \cdot {\textbf{v}}+ m \Delta _{\Gamma _t} h+ b  &   \text {on }\Gamma _t, t\in [0,T_0],\end{aligned}$$

7.13 $$\begin{aligned} {\textbf{v}}_0^-|_{\partial \Omega }&= 0  &   \text {on }\partial \Omega \times [0,T_0],\end{aligned}$$

7.14 $$\begin{aligned} h|_{t=0}=h_0,\qquad {\textbf{v}}^\pm |_{t=0}&= {\textbf{v}}_0^\pm  &   \text {in }\Omega ^\pm _0, \end{aligned}$$

where ${\textbf{v}}^\pm = {\textbf{v}}|_{\Omega ^\pm }$ and $\partial _t^\bullet h$ denotes the material time derivative of $h:\Gamma \rightarrow {\mathbb {R}}$ defined by

$$\begin{aligned} \partial _t^\bullet h = \partial _t {\tilde{h}} +\nabla {\tilde{h}}\cdot (\partial _t X_t)\circ X_t^{-1}\qquad \text {on }\Gamma , \end{aligned}$$

where ${\tilde{h}}:\Gamma (\delta )\rightarrow {\mathbb {R}}$ with ${\tilde{h}}(x,t)= h(P_{\Gamma _t}(x),t)$ for all $x\in \Gamma _t(\delta ), t\in [0,T_0]$ for a sufficiently small $\delta$.

The main result of this subsection is

#### Theorem 7.1

Let $$q>d+2$$, $$T_0\in (0,\infty )$$,

$m\in (0,1)$, and $\Omega , \Gamma _t, \Omega ^\pm _t$, $t\in [0,T_0]$, be as before. Moreover, let

$$\begin{aligned} {\textbf{f}}&\in L^q(0,T_0; L^q(\Omega ))^d,\quad g\in L^q(0,T_0; W^1_q(\Omega \setminus \Gamma _t))\cap W^1_q(0,T_0;{\dot{W}}^{-1}_q(\Omega )), \\&\quad {\textbf{v}}_0 \in W^{2-\frac{2}{q}}_q(\Omega \setminus \Gamma _0)^d,\\ {\textbf{a}}&\in W^{\frac{1}{2}-\frac{1}{2q}}_q(0,T_0;L^q(\Gamma _t))^d\cap L^q(0,T_0; W^{1-\frac{1}{q}}_q(\Gamma _t))^d,\quad h_0 \in W^{3-\frac{2}{q}}_q(\Gamma _0),\\ b&\in W^{1-\frac{1}{2q}}_q(0,T_0;L^q(\Gamma _t))\cap L^q(0,T_0; W^{2-\frac{1}{q}}_q(\Gamma _t)) \end{aligned}$$

satisfy the compatibility conditions

1. $${\textbf{v}}_0|_{\partial \Omega }=0$$, $$\llbracket {\textbf{v}}_0^\pm \rrbracket =0$$,
2. $${\text {div}} {\textbf{v}}_0 = g|_{t=0}$$,
3. $$-P_{T\Gamma _0}\llbracket 2D{\textbf{v}}^\pm _0\rrbracket {\textbf{n}}_{\Gamma _t}= P_{T\Gamma _0} {\textbf{a}}|_{t=0}$$,

then there is a unique solution $({\textbf{v}}, p, h)$ such that

$$\begin{aligned} {\textbf{v}}&\in W^1_q(0,T_0; L^q(\Omega ))^d\cap L^q(0,T_0;W^2_q(\Omega \setminus \Gamma _t))^d,p\in L^q(0,T_0; {\dot{W}}^1_q(\Omega \setminus \Gamma _t)),\\ \llbracket p \rrbracket&\in W^{\frac{1}{2}-\frac{1}{2q}}_q(0,T_0;L^q(\Gamma _t))\cap L^q(0,T_0; W^{1-\frac{1}{q}}_q(\Gamma _t)),\\ h&\in W^{2-\frac{1}{2q}}_q(0,T_0; L^q(\Gamma _t))\cap W^1_q(0,T_0; W^{2-\frac{1}{q}}_q(\Gamma _t))\cap L^q(0,T_0; W^{4-\frac{1}{q}}_q(\Gamma _t)). \end{aligned}$$

Moreover, there is some $C>0$ independent of $m\in (0,1)$ and the data $({\textbf{f}}, g, {\textbf{a}}, b,{\textbf{v}}_0 , h_0)$ such that

7.15 $$\begin{aligned}&\Vert {\textbf{v}}\Vert _{W^1_q(0,T_0; L^q(\Omega ))}+ \Vert {\textbf{v}}\Vert _{L^q(0,T_0;W^2_q(\Omega \setminus \Gamma _t))} + \Vert p\Vert _{L^q(0,T_0; {\dot{W}}^1_q(\Omega \setminus \Gamma _t))}\nonumber \\&\qquad + \Vert \llbracket p \rrbracket \Vert _{W^{\frac{1}{2}-\frac{1}{2q}}_q(0,T_0;L^q(\Gamma _t))} + \Vert \llbracket p \rrbracket \Vert _{L^q(0,T_0; W^{2-\frac{1}{q}}_q(\Gamma _t))}+\Vert h\Vert _{W^{2-\frac{1}{2q}}_q(0,T_0; L^q(\Gamma _t))} \nonumber \\&\qquad + \Vert h\Vert _{W^1_q(0,T_0; W^{1-\frac{1}{q}}_q(\Gamma _t))}+ \Vert h\Vert _{L^q(0,T_0; W^{3-\frac{1}{q}}_q(\Gamma _t))} + m\Vert h\Vert _{L^q(0,T_0; W^{4-\frac{1}{q}}_q(\Gamma _t))}\nonumber \\&\quad \le C\left( \Vert {\textbf{f}}\Vert _{L^q(0,T_0; L^q(\Omega ))}+\Vert g\Vert _{L^q(0,T_0; W^1_q(\Omega \setminus \Gamma _t))}\right. \nonumber \\&\qquad \left. +\Vert {\textbf{a}}\Vert _{W^{\frac{1}{2}-\frac{1}{2q}}_q(0,T_0;L^q(\Gamma _t))\cap L^q(0,T_0; W^{2-\frac{1}{q}}_q(\Gamma _t))}\right. \nonumber \\&\qquad \left. +\Vert b\Vert _{L^q(0,T_0; W^{2-\frac{1}{q}}_q(\Gamma _t))} +\Vert {\textbf{v}}_0\Vert _{W^{2-\frac{2}{q}}_q(\Omega \setminus \Gamma _0)}+\Vert h_0\Vert _{ W^{4-\frac{3}{q}}_q(\Gamma _0)} \right) \end{aligned}$$

Here we define $L^q(0,T_0; W^m_q(\Omega \setminus \Gamma _t))$ such that $g\in L^q(0,T_0; W^m_q(\Omega \setminus \Gamma _t))$ if and only if $g\circ (X,{\text {id}}_{(0,T_0)})\in L^q(0,T_0; W^m_q(\Omega \setminus \Gamma _0))$ for $m\in {\mathbb {N}}_0$ and analogously for the other function spaces involving $\Gamma _t$.

### Case of a Flat Interface

Throughout this section we assume that $\Omega ^\pm _t= {\mathbb {R}}^d_\pm$, $\Gamma _t= {\mathbb {R}}^{d-1}\times \{0\}$, and $\Omega = {\mathbb {R}}^d$. We follow the strategy of [35, Section 8.2]. To this end we first assume that ${\textbf{f}}=g={\textbf{a}}={\textbf{v}}_0=h_0=0$, use a spectral shift, and consider, for $\omega >0$,

7.16 $$\begin{aligned} (\partial _t +\omega ) {\textbf{v}}^\pm -\Delta {\textbf{v}}^\pm +\nabla p^\pm&=0&\qquad&\text {in }{\mathbb {R}}^d_\pm \times (0,\infty ), \end{aligned}$$

7.17 $$\begin{aligned} {\text {div}}{\textbf{v}}^\pm&= 0&\qquad&\text {in }{\mathbb {R}}^d_\pm \times (0,\infty ),\end{aligned}$$

7.18 $$\begin{aligned} \llbracket 2D{\textbf{v}}^\pm -p^\pm {\textbf{I}}\rrbracket e_d&= -\sigma \Delta _{{\mathbb {R}}^{d-1}} h e_d  &   \text {on }{\mathbb {R}}^{d-1}\times \{0\}\times (0,\infty ),\end{aligned}$$

7.19 $$\begin{aligned} \llbracket {\textbf{v}}^\pm \rrbracket&=0  &   \text {on }{\mathbb {R}}^{d-1}\times \{0\}\times (0,\infty ),\end{aligned}$$

7.20 $$\begin{aligned} (\partial _t+ \omega ) h + {\textbf{v}}_d - m \Delta _{{\mathbb {R}}^{d-1}} h&= b  &   \text {on }{\mathbb {R}}^{d-1}\times \{0\}\times (0,\infty ),\end{aligned}$$

7.21 $$\begin{aligned} ({\textbf{v}}, h)|_{t=0}&= (0,0), \end{aligned}$$

where $${\textbf{v}}^\pm ={\textbf{v}}|_{{\mathbb {R}}^d_\pm }$$, $$p^\pm =p|_{{\mathbb {R}}^d_\pm }$$.

The goal of this subsection is to prove

#### Theorem 7.2

For any $$\omega >0$$, $$1<q<\infty $$,

$$\begin{aligned} b \in  _0W^{1-\frac{1}{2q}}_q(0,\infty ;L^q({\mathbb {R}}^{d-1}))\cap L^q(0,\infty ; W^{2-\frac{1}{q}}_q({\mathbb {R}}^{d-1})) \end{aligned}$$

there is a unique solutionsatisfying7.22uniformly in $m\in [0,1]$.

Analogously as in [35] one obtains that $({\textbf{v}}^\pm , p^\pm , h)$ solves (7.16)–(7.21) if and only if

7.23 $$\begin{aligned} (\partial _t+\omega ) h + e_d \cdot ({\mathcal {D}}{\mathcal {N}})^{-1} \begin{pmatrix} 0\\ -\sigma \Delta _{{\mathbb {R}}^{d-1}} h \end{pmatrix}\nonumber \\\nonumber - m \Delta _{{\mathbb {R}}^{d-1}} h&= b\quad \text{ on } {\mathbb {R}}^{d-1}\times \{0\}\times (0,\infty ),\nonumber \\ ({{\textbf {v}}}, h)|_{t=0}&= (0,0), \end{aligned}$$

where ${\mathcal {D}}{\mathcal {N}}$ is the Dirichlet-to-Neumann operator for the two-phase Stokes problem as in [35, Section 8.3.2] and $({\textbf{v}}^\pm , p^\pm )$ is determined by (7.16)–(7.19) and ${\textbf{v}}|_{t=0}=0$ in dependence on *h*. More precisely, the Laplace-Fourier transform of $$u=e_d \cdot ({\mathcal {D}}{\mathcal {N}})^{-1} \begin{pmatrix} 0\\ -\sigma \Delta _{{\mathbb {R}}^{d-1}} h \end{pmatrix}$$ is given by

$$\begin{aligned} {\hat{u}}(\lambda ,\xi )= \frac{-\sigma |\xi |^2}{2\lambda /|\xi |+ 2(\lambda +|\xi |^2)^{\frac{1}{2}}+|\xi |} {\hat{h}}(\lambda ,\xi ), \end{aligned}$$

cf. [35, Equation (8.34)], where we note that $\eta _1=\eta _2= (\lambda +|\xi |^2)^{\frac{1}{2}}+|\xi |$ since $\rho _1=\rho _2=\mu _1=\mu _2=1$ in our case. Hence the Fourier-Laplace transformation of (7.23) is $s_m(\lambda ,|\xi |){\hat{h}}(\lambda ,\xi )= {\hat{b}}(\lambda ,\xi )$, where

$$\begin{aligned} s_m(\lambda ,\tau ) = \lambda + m\tau ^2 +\frac{\sigma \tau ^2}{2\lambda /\tau + 2(\lambda +\tau ^2)^{\frac{1}{2}}+\tau }\quad \text {for }\lambda \in {\mathbb {C}}, \tau \in {\mathbb {C}}\setminus \{0\}. \end{aligned}$$

We note that $s_0(\lambda ,|\xi |)$ coincides with the symbol $s_{0,0}(\lambda ,\xi )$ studied in [35, Section 8.3.4], for which it was shown

$$\begin{aligned} |s_{0,0}(\lambda ,\xi )|\le C_\eta (|\lambda |+|\xi |) \qquad \text {for all }\lambda \in \Sigma _{\pi /2+\eta }, \xi \in \left( \Sigma _\eta \cup -\Sigma _\eta \right) ^{n-1}, \end{aligned}$$

cf. [35, Equation (8.50)] for every $\eta \in [0,\frac{\pi }{2})$. Hence for every $\eta \in [0,\frac{\pi }{2})$ there is some $C_\eta$ such that

$$\begin{aligned} |s_m(\lambda ,\tau )|\le C_\eta (|\lambda |+|\tau |+m|\tau |^2) \qquad \text {for all }\lambda \in \Sigma _{\pi /2+\eta }, \tau \in \Sigma _\eta , \end{aligned}$$

uniformly in $m\in [0,1]$. The essential point is that we have the same kind of lower bound.

#### Lemma 7.3

For any $\omega _0>0$ there is some $\eta >0$ and $c>0$ such that

7.24 $$\begin{aligned} |s_m(\lambda ,\tau )|\ge c(|\lambda |+|\tau |+m|\tau |^2) \qquad \text {for all }\lambda \in \Sigma _{\pi /2+\eta },|\lambda |\ge \omega _0, \tau \in \Sigma _\eta ,\nonumber \\ \end{aligned}$$

#### Proof

First of all, we note that

$$\begin{aligned} s_m(\lambda ,\tau ) = \lambda + m\tau ^2+\sigma \tau k(z) \qquad \text {with } z= \frac{\lambda }{\tau ^2}, \tau \ne 0, \end{aligned}$$

where it was shown in [35, p. 392] that for any $\vartheta \in [0,\pi )$ there is some $C_\vartheta >0$ such that

$$\begin{aligned} |k(z)|\le \frac{C_\vartheta }{1+|z|}\qquad \text {for all }z\in \Sigma _\vartheta . \end{aligned}$$

Moreover, $${\text {Re}} k(z)>0$$ if $${\text {Re}} z\ge 0$$.

We first show that (7.24) holds for $\lambda \in \Sigma _{\pi /2-\delta }, \tau \in \Sigma _\eta$ and any $\delta \in (0,\pi /4)$ and sufficiently small $\eta >0$ (depending on $\delta$). To this end we use that for any $\eta >0$ there is some $C_\eta >0$ such that

$$\begin{aligned} |z|\le C_\eta {\text {Re}} z \qquad \text {for all } z\in \Sigma _{\pi /2-\eta }. \end{aligned}$$

Furthermore, observe that

$$\begin{aligned} \frac{2\lambda }{\tau }+ 2(\lambda +\tau ^2)^{\frac{1}{2}}+\tau \in \Sigma _{\pi /2-\delta /2}\quad \text {for all }\lambda \in \Sigma _{\pi /2-\delta }, \tau \in \Sigma _{\delta /4} \end{aligned}$$

and therefore

$$\begin{aligned} \frac{\sigma \tau ^2}{2\lambda /\tau + 2(\lambda +\tau ^2)^{\frac{1}{2}}+\tau }\in \Sigma _{\pi /2-\delta /3}\quad \text {for all }\lambda \in \Sigma _{\pi /2-\delta }, \tau \in \Sigma _{\eta } \end{aligned}$$

if $\eta >0$ is sufficiently small. Since $\lambda , m\tau ^2\in \Sigma _{\pi /2-\delta /3}$ for all $\lambda \in \Sigma _{\pi /2-\delta }, \tau \in \Sigma _{\eta }$ as well, we conclude

$$\begin{aligned} |s_m(\lambda ,\tau )|&\ge {\text {Re}} s_m(\lambda ,\tau ) = {\text {Re}} \lambda + m{\text {Re}}\tau ^2+ {\text {Re}} \frac{\sigma \tau ^2}{2\lambda /\tau + 2(\lambda +\tau ^2)^{\frac{1}{2}}+\tau }\\&\ge c_\eta \left( |\lambda |+ m|\tau |^2+ |\tau ||k(z)|\right) \qquad \text {with }z=\frac{\lambda }{\tau ^2} \end{aligned}$$

provided $\eta >0$ is sufficiently small. Now, if $|z|\le 1$, there is some $c>0$ such that $|k(z)|\ge c$ if ${\text {Re}} z\ge 0$ and $|z|\le 1$ and we conclude

$$\begin{aligned} |s_m(\lambda ,\tau )|\ge c_\eta \left( |\lambda |+ m|\tau |^2+ |\tau |\right) \qquad \text {if }|z|\le 1. \end{aligned}$$

On the other hand, if $|z|\ge 1$, then $|\lambda |\ge |\tau |^2$ and therefore

$$\begin{aligned} |s_m(\lambda ,\tau )|\ge c_\eta \left( |\lambda |+ m|\tau |^2+ |\tau |\right) \quad \text {for all }\lambda \in \Sigma _{\pi /2-\delta },|\lambda |\ge \omega _0, \tau \in \Sigma _{\eta } \end{aligned}$$

uniformly in $m\in [0,1]$ if $\eta >0$ is sufficiently small.

Next we consider $\lambda \in \Sigma _{\pi /2+\delta }\setminus \Sigma _{\pi /2-\delta }$ for $\delta >0$ sufficiently small. To this end we note that, since ${\text {Re}} k(z)>0$ if ${\text {Re}} z \ge 0$ and $k(z)\rightarrow _{z\in \Sigma _{\vartheta },|z|\rightarrow \infty } 0$ for every $\vartheta \in [0,\pi )$, we have that

$$\begin{aligned} K:= \overline{\left\{ k(z): z\in \Sigma _{\pi /2+\eta }\right\} }\subseteq \{z\in {\mathbb {C}}: {\text {Re}} z>0\} \end{aligned}$$

if $\eta >0$ is sufficiently small. Moreover, *K* is compact. Therefore, there is some $\delta >0$ such that

$$\begin{aligned} K \subseteq \Sigma _{\pi /2-3\delta }. \end{aligned}$$

Moreover, it is easy to prove that there is some $C_\delta >0$ such that

$$\begin{aligned} |z|+|w|\le C_\delta |z+w|\qquad \text {for all }z \in \Sigma _{\pi /2+\delta }\setminus \Sigma _{\pi /2-\delta }, w\in \Sigma _{\delta } \end{aligned}$$

provided that $\delta \in (0,\pi /4)$. Hence

$$\begin{aligned} |s_m(\lambda ,\tau )|&\ge c_{\delta } \left( |\lambda |+|m\tau ^2+\sigma \tau k(z)|\right) \\&\ge c_{\delta } \left( |\lambda |+m{\text {Re}} (\tau ^2)+\sigma {\text {Re}}(\tau k(z))\right) \\&\ge c_{\delta } \left( |\lambda |+m|\tau ^2|+\sigma |\tau ||k(z)|\right) , \end{aligned}$$

and, with the same arguments as before,

$$\begin{aligned} |s_m(\lambda ,\tau )|&\ge c_{\delta } \left( |\lambda |+m|\tau ^2|+|\tau |\right) \end{aligned}$$

for all $\lambda \in \Sigma _{\pi /2+\delta }\setminus \Sigma _{\pi /2-\delta }$, $|\lambda |\ge \omega _0$, $\tau \in \Sigma _\eta$ uniformly in $m\in [0,1]$ if $\eta >0$ is sufficiently small. This finishes the proof. $\square$

Now we can proceed as in [35, Section 8.3.3]. Let $D_n^{\frac{1}{2}}=(-\Delta _{x'})^{\frac{1}{2}}$ be the Fourier multiplication operator with symbol $|\xi |$, $\xi \in {\mathbb {R}}^{d-1}$. Then $D_n^{\frac{1}{2}}$ possesses an ${\mathcal {R}}$-bounded functional calculus in $W^{2-\frac{1}{q}}_q({\mathbb {R}}^{d-1})$, for any $1<q<\infty$. Therefore

$$\begin{aligned} (\lambda + D_n^{\frac{1}{2}}+ m D_n) s_{m}^{-1}(\lambda , D_n^{\frac{1}{2}}) \end{aligned}$$

is ${\mathcal {R}}$-bounded and possesses a bounded ${\mathcal {H}}^\infty$-calculus on $\Sigma _{\pi /2+\eta }\setminus B_{\omega _0}(0)$ for some $\eta >0$ and any $\omega _0>0$, which is also uniformly bounded in $m\in [0,1]$. Hence the operator-valued ${\mathcal {H}}^\infty$-calculus of $G=\partial _t +\omega$ on $_0H^r_q(0,\infty ; K_q({\mathbb {R}}^{d-1}))$ for $\omega >0$, $s,r\in {\mathbb {R}}$ and $K=H,W$ yields that

$$\begin{aligned} (\partial _t +\omega + D_n^{\frac{1}{2}}+ m D_n) s_{m}^{-1}(\partial _t +\omega , D_n^{\frac{1}{2}}) \in {\mathcal {L}} \left(  _0H^r_q(0,\infty ; K_q({\mathbb {R}}^{d-1}))\right) \end{aligned}$$

is uniformly bounded in $m\in [0,1]$ for any $s,r\in {\mathbb {R}}$. Hence we obtain for $h= s_{m}^{-1}(\lambda , D_n^{\frac{1}{2}})b$ and $b\in {\mathbb {E}}$ that *h* solves (7.23) and satisfies

$$\begin{aligned} \Vert \omega h\Vert _{{\mathbb {E}}}+ \Vert \partial _t h\Vert _{{\mathbb {E}}}+ \Vert \nabla _{x'} h\Vert _{{\mathbb {E}}}+m \Vert \nabla _{x'}^2 h\Vert _{{\mathbb {E}}}\le C_{r,s,p} \Vert b\Vert _{{\mathbb {E}}} \end{aligned}$$

uniformly in $m\in [0,1]$, where ${\mathbb {E}}:=L^q(0,\infty ; W^{2-\frac{1}{q}}_q({\mathbb {R}}^{d-1}))$ or ${\mathbb {E}}:= _0H^k_q(0,\infty ; L^q({\mathbb {R}}^{d-1}))$, $k=0,1$. By real interpolation we obtain that the same is true for $b\in {\mathbb {E}}= _0W^{1-\frac{1}{2q}}_q(0,\infty ; L^q({\mathbb {R}}^{d-1}))$. This shows existence of a solution as in Theorem 7.2 satisfying (7.22) uniformly in $m\in [0,1]$, where the existence of $({\textbf{v}},p)$ and the corresponding estimates follow from [35, Corollary 8.3.3]. Uniqueness can be shown by a standard duality argument. Hence Theorem 7.2 is proved.

### Proof of Theorem 7.1

First we assume that $\Gamma _t=\Gamma$ is independent of $t\in [0,T_0]$. In this case one proves the result by the same localization and perturbation argument and reduction to semi-homogeneous data as in [35, Section 8.2] using the result for a flat interface due to Theorem 7.2. The only difference is related to the new term “$m\Delta _{\Gamma } h$” in the evolution equation for *h*. These extra-terms can be controlled by $m\Vert h\Vert _{L^q(0,T_0;W^{4-\frac{1}{q}}_q)}$ (uniformly in $m\in [0,1]$), while all other terms can be controlled by $\Vert h\Vert _{L^q(0,T_0;W^{3-\frac{1}{q}}_q)}$ as in the case $m=0$. Here we note that in the proof one can reduce to $h_0=0$ by subtracting some ${\tilde{h}}\in L^q(0,T_0;W^{4-\frac{1}{q}}_q(\Gamma ))\cap W^1_q(0,T_0; W^{2-\frac{1}{q}}_q(\Gamma ))$ from *h*, which exists because of

$$\begin{aligned} W^{4-\frac{3}{q}}_q(\Gamma )=(W^{2-\frac{1}{q}}_q(\Gamma ), W^{4-\frac{1}{q}}_q(\Gamma ))_{1-\frac{1}{q},q} \end{aligned}$$

since $1-\frac{1}{q}, 1-\frac{3}{q}\not \in {\mathbb {Z}}$ due to $q>d+2>3$ and by the trace method for real interpolation spaces.

Now, if $\Gamma _t$, $t\in [0,T_0]$, are smoothly evolving interfaces as before, we fix an arbitrary $t_0\in [0,T_0]$ and define $J_{t_0}:= [\max (t_0-\delta ,0), \min (T_0,t_0+\delta )]$ for $\delta >0$ and ${\tilde{X}}:{{\overline{\Omega }}}\times J_{t_0} \rightarrow {{\overline{\Omega }}} \times J_{t_0}$ by ${\tilde{X}}(x,t)= (X_t(X_{t_0}^{-1} (x)),t)$ for all $x\in \Omega$, $t\in J_{t_0}$. Then (7.8)–(7.13) with $J_{t_0}$ instead of $[0,T_0]$ is equivalent to a system of the form

7.25 $$\begin{aligned} \partial _t {{\tilde{{\textbf{v}}}}}^\pm -\Delta {{\tilde{{\textbf{v}}}}}^\pm +\nabla {\tilde{p}}^\pm&= \tilde{{\textbf{f}}} +K_{{\textbf{f}}}&\qquad&\text {in }\Omega ^\pm _{t_0}\times J_{t_0}, \end{aligned}$$

7.26 $$\begin{aligned} {\text {div}}{\textbf{v}}^\pm&= g +K_g&\qquad&\text {in }\Omega ^\pm _{t_0}\times J_{t_0},\end{aligned}$$

7.27 $$\begin{aligned} -\llbracket 2D{\textbf{v}}^\pm -p^\pm {\textbf{I}}\rrbracket {\textbf{n}}_{\Gamma _t}&= \sigma \Delta _{\Gamma _t} h {\textbf{n}}_{\Gamma _t} + {\textbf{a}}+K_{{\textbf{a}}}  &   \text {on }\Gamma _{t_0}\times J_{t_0},\end{aligned}$$

7.28 $$\begin{aligned} \llbracket {\textbf{v}}^\pm \rrbracket&=0  &   \text {on }\Gamma _{t_0}\times J_{t_0},\end{aligned}$$

7.29 $$\begin{aligned} \partial _t h&= {\textbf{n}}_{\Gamma _t} \cdot {\textbf{v}}+ m \Delta _{\Gamma _{t_0}} h+ b +K_b  &   \text {on }\Gamma _{t_0}\times J_{t_0},\end{aligned}$$

7.30 $$\begin{aligned} {\textbf{v}}_0^-|_{\partial \Omega }&= 0  &   \text {on }\partial \Omega \times J_{t_0}, \end{aligned}$$

where $(K_{{\textbf{f}}}, K_g, K_{{\textbf{a}}}, K_b)$ depend linearly on $({\textbf{v}}^\pm ,p^\pm , h)$ and

$$\begin{aligned}&\Vert K_{\textbf{f}}\Vert _{L^q(J_{t_0}; L^q(\Omega ))}+\Vert K_g\Vert _{L^q(J_{t_0}; W^1_q(\Omega \setminus \Gamma _{t_0}))}+\Vert K_b\Vert _{L^q(J_{t_0}; W^{2-\frac{1}{q}}_q(\Gamma _{t_0}))}\\&\qquad +\Vert K_{\textbf{a}}\Vert _{W^{\frac{1}{2}-\frac{1}{2q}}_q(J_{t_0};L^q(\Gamma _{t_0}))\cap L^q(J_{t_0}; W^{2-\frac{1}{q}}_q(\Gamma _{t_0}))}\\&\quad \le C(\delta )\left( \Vert {\textbf{v}}\Vert _{W^1_q(J_{t_0}; L^q(\Omega ))}+ \Vert {\textbf{v}}\Vert _{L^q(J_{t_0};W^2_q(\Omega \setminus \Gamma _{t_0}))} + \Vert p\Vert _{L^q(J_{t_0}; W^1_q(\Omega \setminus \Gamma _{t_0}))}\right. \\&\qquad + \Vert \llbracket p \rrbracket \Vert _{W^{\frac{1}{2}-\frac{1}{2q}}_q(J_{t_0};L^q(\Gamma _{t_0}))}+ \Vert \llbracket p \rrbracket \Vert _{L^q(J_{t_0}; W^{2-\frac{1}{q}}_q(\Gamma _{t_0}))}+ \Vert h\Vert _{L^q(J_{t_0}; W^{3-\frac{1}{q}}_q(\Gamma _{t_0}))}\\&\qquad \left. + \Vert h\Vert _{W^1_q(J_{t_0}; W^{1-\frac{1}{q}}_q(\Gamma _{t_0}))}+ m\Vert h\Vert _{L^q(J_{t_0}; W^{4-\frac{1}{q}}_q(\Gamma _{t_0}))}\right) , \end{aligned}$$

where

$$\begin{aligned} {\tilde{{\textbf{v}}}}^\pm&:= {\textbf{v}}^\pm \circ {\tilde{X}}|_{\Omega ^\pm _{t_0}},\quad p^\pm := p^\pm \circ {\tilde{X}}|_{\Omega ^\pm _{t_0}}, \quad {\tilde{h}}:= h\circ {\tilde{X}}|_{\Gamma _{t_0}},\\ \tilde{{\textbf{f}}}&:= {\textbf{f}}\circ {\tilde{X}},\quad {\tilde{g}}:= g \circ {\tilde{X}}, \quad \tilde{{\textbf{a}}}:= {\textbf{a}}\circ {\tilde{X}}|_{\Gamma _{t_0}} , \quad {\tilde{b}}:= b\circ {\tilde{X}}|_{\Gamma _{t_0}} \end{aligned}$$

and $C(\delta )\rightarrow 0$ as $\delta \rightarrow 0$ since ${\tilde{X}}\rightarrow I$ in $C^k(\Omega ^\pm _{t_0}\times J_{t_0})$ as $\delta \rightarrow 0$ for any $k\in {\mathbb {N}}$. Hence by a standard Neumann series argument (7.25)–(7.30) together with ${{\tilde{{\textbf{v}}}}}|_{t={\tilde{t}}_0}={\textbf{v}}_0\circ {\tilde{X}}(\cdot ,{\tilde{t}}_0), {\tilde{h}}|_{t={\tilde{t}}_0}=h_0\circ {\tilde{X}}(\cdot ,{\tilde{t}}_0)|_{\Gamma _{{\tilde{t}}_0}}$, ${\tilde{t}}_0:= \max (0,t_0-\delta )$, possesses a unique solution $({\tilde{{\textbf{v}}}}^\pm ,{\tilde{p}}^\pm , {\tilde{h}})$ for every $({\textbf{f}},g,{\textbf{a}}, b, {\textbf{v}}_0,h_0)$ as in Theorem 7.1 with $[0,T_0]$ replaced by $J_{t_0}$ and the initial time 0 replaced by ${\tilde{t}}_0=\max (0,t_0-\delta )$ provided $\delta =\delta (t_0)>0$ is sufficiently small. Transforming back, this yields a unique solution $({\textbf{v}}^\pm ,p^\pm ,{h})$ of (7.8)–(7.13) together with ${\textbf{v}}|_{t=\max (0,t_0-\delta )}={\textbf{v}}_0, {h}|_{t=\max (0,t_0-\delta )}=h_0$ for every $({\textbf{f}},g,{\textbf{a}}, b, {\textbf{v}}_0,h_0)$ as in Theorem 7.1 with $[0,T_0]$ replaced by $J_{t_0}$ and the initial time 0 replaced by ${\tilde{t}}_0$. Moreover, the Neumann series argument also shows that (7.15) with the same replacements as before holds true uniformly in $m\in [0,1]$.

Since $\{(t_0-\delta (t_0), t_0+\delta (t_0)): t_0\in [0,T_0]\}$ is an open covering of $[0,T_0]$, there are finitely many $0=t_0< t_1<\ldots< t_N<T_0$ and $\delta _j>0$ such that $[0,T_0] =\bigcup _{j=0}^N J_{t_j}$ and we can solve (7.8)–(7.13) on $J_{t_j}=[\max (0,t_j-\delta ),\min (t_j+\delta ,T_0)]$ together with ${{\tilde{{\textbf{v}}}}}|_{t=\max (0,t_j-\delta _j)}={\textbf{v}}_0, {\tilde{h}}|_{t=\max (0,t_j-\delta _j)}=h_0$ uniquely as before. Hence we can solve (7.8)–(7.14) by solving successively (7.8)–(7.13) on $J_{t_j}$, $j=0,\ldots , N$ with initial values $({\textbf{v}}_0,h_0)$, $({\textbf{v}}|_{t=t_{j-1}}, h|_{t=t_{j-1}})$ for $j=2,\ldots , N$. Finally, since (7.15) holds true uniformly in $m\in [0,1]$ on the intervals $J_{t_j}$, $j=0,\ldots , N$, one also obtains (7.15) on $[0,T_0]$ uniformly in $m\in [0,1]$.

### Existence of Solutions for the Transformed System

The idea of the proof is to represent $\Gamma ^m_t$ from the solution of (7.1)–(7.7) for every $t\in [0,T_0]$ as a graph over $\Gamma _t$, where $\Gamma _t$, $t\in [0,T_0]$, is from the smooth solution of (1.2a)–(1.2g) and to transform (7.1)–(7.7) to a corresponding perturbed system in $\Omega _t^\pm$, $t\in [0,T_0]$ with the aid of the Hanzawa-Transformation associated to $\Gamma _t$. To this end, let us denote that

$$\begin{aligned} \Gamma _t (3\delta ) = \{ x\in {\mathbb {R}}^d: |d_{\Gamma _t}(x)|<3\delta \}, \qquad t\in [0,T_0], \end{aligned}$$

where

$$\begin{aligned} d_{\Gamma _t}(x) = {\left\{ \begin{array}{ll} {\text {dist}}(x,\Gamma _t)& \text {if }x\in \Omega ^+_t,\\ -{\text {dist}}(x,\Gamma _t)& \text {else} \end{array}\right. } \end{aligned}$$

is the signed distance to $\Gamma _t$. Since $(\Gamma _t)_{t\in [0,T_0]}$ are smoothly evolving, compact, and $[0,T_0]$ is compact, there is some $\delta >0$ such that for every $x\in \Gamma _t(3\delta )$, $t\in [0,T_0]$ there is a unique closest point $P_{\Gamma _t}(x)\in \Gamma _t$ and

$$\begin{aligned} \Gamma (3\delta ):=\bigcup _{t\in [0,T_0]} \Gamma _t(3\delta )\times \{t\} \ni (x,t)\mapsto (d_{\Gamma _t}, P_{\Gamma _t})\in {\mathbb {R}}^{d+1} \end{aligned}$$

is smooth. Moreover, we choose $\delta >0$ so small that $\Gamma (3\delta )\subseteq \Omega \times [0,T_0]$.

Furthermore, for a given continuous “height function” $h:\Gamma \rightarrow {\mathbb {R}}$ let

$$\begin{aligned} \theta _h:\Gamma \rightarrow {\mathbb {R}}^n:x\mapsto x+h(x,t){\textbf{n}}_{\Gamma _t}(x). \end{aligned}$$

Here $\Gamma$ is defined as in (1.3). Then $\theta _h$ is injective provided that $\Vert h\Vert _{C^0(\Gamma )}<\delta$. Moreover, we define the *Hanzawa transformation* associated to $\Gamma$ as

7.31 $$\begin{aligned} \Theta _h(x,t)= x+\chi (d_{\Gamma _t}(x)/\delta )h(P_{\Gamma _t}(x),t){\textbf{n}}_{\Gamma _t}(P_{\Gamma _t}(x)), \end{aligned}$$

where $\chi \in C^\infty ({\mathbb {R}})$ such that $\chi (s)=1$ for $|s|\le 2\delta$ and $\chi (s)=0$ for $|s|>\frac{2}{3}$ as well as $|\chi '(s)|\le 4$ for all $s\in {\mathbb {R}}$, and $\Vert h\Vert _{C^0(\Gamma )}<\delta$. Then $\Theta _h(.,t):\Omega \rightarrow \Omega$ is a smooth diffeomorphism for every $t\in [0,T_0]$, cf. e.g. [35, Chapter 1, Section 3.2]. Hence for any $h:\Gamma \rightarrow {\mathbb {R}}$ that is continuously differentiable with $\Vert h\Vert _{C^0(\Gamma )}<\delta$ we have that $(\Gamma ^h_t)_{t\in [0,T_0]}:=(\theta _h(\Gamma _t,t))_{t\in [0,T_0]}$ is an oriented, compact evolving $C^1$-manifold, such that $\Gamma _t^h$ is a $C^2$-manifold for every $t\in [0,T_0]$ if $h(\cdot ,t):\Gamma _t\rightarrow {\mathbb {R}}$ is twice continuously differentiable.

In the following we look for a solution of (7.1)–(7.7) such that $\Gamma _t^m= \Gamma ^h_t$ for all $t\in [0,T_0]$ a sufficiently regular $h:\Gamma \rightarrow {\mathbb {R}}$ with $\Vert h\Vert _{C^0(\Gamma )}<\delta$. Then $({\textbf{v}}_m^\pm , p_m^\pm , (\Gamma _t^m)_{t\in [0,T_0]})$ solves (7.1)–(7.7) if and only if

$$\begin{aligned} \begin{array}{ll} {{\textbf {v}}}^\pm (x,t):={{\textbf {v}}}_m^\pm (\Theta _{h}(x,t),t),\quad p^\pm (x,t)=p^\pm _m(\Theta _h(x,t),t) &  \ \text{ for } x\in \Omega ^\pm _t,t\in [0,T_0],\\ \quad h(x,t) := h_m(P_{\Gamma _t}(\theta _h(x,t)),t) &  \ \text{ for } x\in \Gamma _t,t\in [0,T_0] \end{array} \end{aligned}$$

solves the transformed system

7.32 $$\begin{aligned} \partial _t {\textbf{v}}^\pm -\Delta {\textbf{v}}^\pm +\nabla p^\pm&= a^\pm (h;D_x)({\textbf{v}}^\pm ,p^\pm )\nonumber \\&\quad + \partial _t \Theta _h\cdot \nabla _h {\textbf{v}}^\pm - {\textbf{v}}^\pm \cdot \nabla _h {\textbf{v}}^\pm&\quad&\text {in} \ \Omega ^\pm , \end{aligned}$$

7.33 $$\begin{aligned} {\text {div}}{\textbf{v}}^\pm&= {\text {tr}}((I-A(h))\nabla {\textbf{v}}^\pm )=:g(h){\textbf{v}}^\pm&\quad&\text {in} \ \Omega ^\pm ,\end{aligned}$$

7.34 $$\begin{aligned} \llbracket {\textbf{v}}\rrbracket&= 0&\quad&\text {on} \ \Gamma ,\end{aligned}$$

7.35 $$\begin{aligned} \llbracket 2D{\textbf{v}}^\pm -p^\pm {\textbf{I}}\rrbracket {\textbf{n}}_{\Gamma _t}&= t(h;D_x) ({\textbf{v}},p) + \sigma K(h) {\textbf{n}}_{\Gamma ^h_t}\circ \theta ^h_t&\quad&\text {on} \ \Gamma ,\end{aligned}$$

7.36 $$\begin{aligned} {\textbf{v}}^-|_{\partial \Omega }&=0  &   \text {on}\ \partial \Omega \times (0,T_0), \end{aligned}$$

7.37 $$\begin{aligned} \partial _t^\bullet h -{\textbf{n}}_{\Gamma _t} \cdot {\textbf{v}}&= m K(h) +\big ({\textbf{n}}_{\Gamma ^h_t}\circ \theta ^h_t -{\textbf{n}}_{\Gamma _t}\big )\cdot {\textbf{v}}  &   \text {on }\Gamma , \end{aligned}$$

7.38 $$\begin{aligned} {\textbf{v}}|_{t=0}&= {\textbf{v}}_0&\quad&\text {on} \ \Omega _0^\pm , \end{aligned}$$

where

$$\begin{aligned} a^\pm (h;D_x)({\textbf{v}}^\pm ,p^\pm )&= {\text {div}}_h(\nabla _h {\textbf{v}}^\pm )-\Delta {\textbf{v}}^\pm +(\nabla -\nabla _h)p^\pm ,\\ \nabla _h&= A(h)\nabla ,\quad {\text {div}}_h u = {\text {tr}} (\nabla _h u),\quad A(h)=D_x\Theta _h^{-T},\\ t(h,D_x)({\textbf{v}},p)&= [\![({\textbf{n}}_{\Gamma _0}-{\textbf{n}}_h)\cdot (2\mu ^\pm D {\textbf{v}}-pI)+2{\textbf{n}}_h\cdot {\text {sym}}(\nabla {\textbf{v}}-\nabla _h {\textbf{v}})]\!],\\ {\textbf{n}}_h&= \frac{A(h){\textbf{n}}_{\Gamma _0}}{|A(h){\textbf{n}}_{\Gamma _0}|},\quad K(h)= H_{\Gamma ^h_t}\circ \theta ^h_t. \end{aligned}$$

For the fixed-point argument we introduce the solution space

$$\begin{aligned} {\mathbb {E}}_m(T_0)&:= {\mathbb {E}}_1(T_0)\times {\mathbb {E}}_2(T_0)\times {\mathbb {E}}_3(T_0)\times {\mathbb {E}}_{4,m}(T_0),\\ {\mathbb {E}}(T_0)&:= {\mathbb {E}}_1(T_0)\times {\mathbb {E}}_2(T_0)\times {\mathbb {E}}_3(T_0)\times {\mathbb {E}}_4(T_0),\\ {\mathbb {E}}_1(T_0)&:= _0W^1_q(0,T_0; L^q(\Omega ))^d\cap L^q(0,T_0;W^2_q(\Omega \setminus \Gamma _t))^d,\\ {\mathbb {E}}_2(T_0)&:= L^q(0,T_0; {\dot{W}}^1_q(\Omega \setminus \Gamma _t)),\\ {\mathbb {E}}_3(T_0)&:= _0 W^{\frac{1}{2}-\frac{1}{2q}}_q(0,T_0;L^q(\Gamma _t))\cap L^q(0,T_0; W^{1-\frac{1}{q}}_q(\Gamma _t)),\\ {\mathbb {E}}_{4,m}(T_0)&:= W^{2-\frac{1}{2q}}_q(0,T_0;L^q(\Gamma _t))\cap  _0W^1_q(0,T_0; W^{2-\frac{1}{q}}_q(\Gamma _t))\cap L^q(0,T_0; W^{4-\frac{1}{q}}_q(\Gamma _t)),\\ {\mathbb {E}}_4(T_0)&:= W^{2-\frac{1}{2q}}_q(0,T_0;L^q(\Gamma _t))\cap  _0W^1_q(0,T_0; W^{2-\frac{1}{q}}_q(\Gamma _t))\cap L^q(0,T_0; W^{3-\frac{1}{q}}_q(\Gamma _t)), \end{aligned}$$

where ${\mathbb {E}}_{4,m}(T_0)$ is normed by

$$\begin{aligned} \Vert h\Vert _{{\mathbb {E}}_{4,m}(T_0)}&:= \Vert h\Vert _{{\mathbb {E}}_{4}(T_0)} + m \Vert h\Vert _{L^q(0,T_0; W^{4-\frac{1}{q}}_q(\Gamma _t))}+m\Vert h\Vert _{W^{1-\frac{1}{2q}}_q(0,T_0; W^2_q(\Gamma _t))}\\&\quad +m\Vert h|_{t=0}\Vert _{W^{4-\frac{3}{q}}_q(\Gamma _0)}\\ \Vert h\Vert _{{\mathbb {E}}_{4}(T_0)}&:= \Vert h\Vert _{W^{2-\frac{1}{q}}_q(0,T_0; L^q(\Gamma _t))} + \Vert h\Vert _{W^1_q(0,T_0; W^{2-\frac{1}{q}}_q(\Gamma _t))} \\&\quad + \Vert h\Vert _{L^q(0,T_0; W^{3-\frac{1}{q}}_q(\Gamma _t)}+\Vert h|_{t=0}\Vert _{W^{3-\frac{3}{q}}_q(\Gamma _0)} \end{aligned}$$

and ${\mathbb {E}}_1(T_0)$, ${\mathbb {E}}_2(T_0)$ are normed in a standard manner. We note that in comparison to [27] the conditions ${\textbf{w}}|_{t=0}=0$, $h|_{t=0}=0$ are already included in the definition of ${\mathbb {E}}_m(T_0)$. Moreover, we define the space for the right-hand side as

$$\begin{aligned} {\mathbb {F}}(T_0)&:= {\mathbb {F}}_1(T_0)\times {\mathbb {F}}_2(T_0)\times {\mathbb {F}}_3(T_0)\times {\mathbb {F}}_4(T_0)\\ {\mathbb {F}}_1(T_0)&:=L^q(0,T_0; L^q(\Omega ))^d,\quad {\mathbb {F}}_2(T_0):=L^q(0,T_0; W^1_q(\Omega \setminus \Gamma _t))\cap W^1_q(0,T;{\dot{W}}^{-1}_q(\Omega )),\\ {\mathbb {F}}_3(T_0)&:= W^{\frac{1}{2}-\frac{1}{2q}}_q(0,T_0;L^q(\Gamma _t))^d\cap L^q(0,T; W^{1-\frac{1}{q}}_q(\Gamma _t))^d,\\ {\mathbb {F}}_4(T_0)&:= W^{1-\frac{1}{2q}}_q(0,T_0;L^q(\Gamma _t))\cap L^q(0,T; W^{2-\frac{1}{q}}_q(\Gamma _t)) \end{aligned}$$

normed in the standard manner.

Then (7.32)–(7.37) can be written as

7.39 $$\begin{aligned} L z + m {\bar{L}} z&= N(z)+ m{\bar{N}}(z), \end{aligned}$$

for $z= ({\textbf{v}},p, \llbracket p \rrbracket , h)\in {\mathbb {E}}(T_0)$ with $\Vert h\Vert _{C^0(\Gamma )}<\delta$, where

$$\begin{aligned} Lz&= (L_1({\textbf{v}}, p), L_2 {\textbf{v}}, L_3 ({\textbf{v}},\pi ,h), L_4({\textbf{v}}, h)), \\ N(z)&= (N_1({\textbf{v}}, p), N_2 ({\textbf{v}},h), N_3 ({\textbf{v}},\pi , h), N_4({\textbf{v}}, h)) \end{aligned}$$

for $z=({\textbf{v}},p,\pi ,h)$ with $\Vert h\Vert _{C^0(\Gamma )}<\delta$ are defined as in [27, Section 4]. More precisely,

$$\begin{aligned} L_1 ({\textbf{v}}, p)&:= \partial _t {\textbf{v}}-\Delta {\textbf{v}}+\nabla p,\quad \\ L_2 {\textbf{v}}&:= {\text {div}}{\textbf{v}},\\ L_3 ({\textbf{v}},p,h)&:= -\llbracket 2D{\textbf{v}}^\pm \rrbracket {\textbf{n}}_{\Gamma _t}- \pi {\textbf{n}}_{\Gamma _t} -(\Delta _{\Gamma _t} h){\textbf{n}}_{\Gamma _t}\\ L_4 ({\textbf{v}},h)&:= \partial _t^\bullet h - {\textbf{n}}_{\Gamma _t} \cdot {\textbf{v}}+ {\textbf{v}}_0\cdot \nabla _{\Gamma _t} h \end{aligned}$$

and

$$\begin{aligned} N_1 ({\textbf{v}}, p,h)&:= F(h,{\textbf{v}})\nabla {\textbf{v}}+ M_4(h):\nabla ^2{\textbf{v}}+M_1(h)\nabla p,\quad N_2 ({\textbf{v}},h) := M_1(h):\nabla {\textbf{v}},\\ N_3 ({\textbf{v}},h)&:= G_\tau (h) \nabla {\textbf{v}}+(G_{\nu }(h)\nabla {\textbf{v}}+ G_\gamma (h)){\textbf{n}}_{\Gamma _t}\\ N_4 ({\textbf{v}},h)&:= ([M_0(h)-I]\nabla _{\Gamma _t} h)\cdot {\textbf{v}}+({\textbf{v}}-{\textbf{v}}_0)\cdot \nabla _{\Gamma _t} h, \end{aligned}$$

where $F, M_1,\ldots , M_4$ are defined as in [27, Section 2], with the only difference that the time-independent reference surface $\Sigma$ is replaced by the smoothly evolving reference surface $\Gamma _t$, $t\in [0,T_0]$ and $\partial _t h$ is replaced by $\partial _t^\bullet h$. Moreover,

$$\begin{aligned} {\bar{L}} z&= (0,0,0, \Delta _{\Gamma _t} h),\\ {\bar{N}} z&= (0,0,0, K(h)-\Delta _{\Gamma _t}h) \end{aligned}$$

for $z=({\textbf{v}},p,\pi , h)$. Let us note that

$$\begin{aligned} L z_0 = N(z_0), \end{aligned}$$

for $z_0 = ({\textbf{v}}_0, p_0,0)$, where ${\textbf{v}}_0|_{\Omega ^\pm }= {\textbf{v}}_0^{\pm }$, $p_0|_{\Omega ^\pm }= p_0^{\pm }$ is the solution of the limit system (1.2a)–(1.2g). Therefore (7.39) is equivalent to

7.40 $$\begin{aligned} {\mathcal {L}}_mw&:= L w + m {\bar{L}} w- DN(z_0)w \nonumber \\  &= N(w+z_0)-N(z_0)-DN(z_0)w+m {\bar{N}}(w+z_0)=: {\mathcal {N}}_m(w) \end{aligned}$$

for $w = ({\textbf{u}}, \pi , \llbracket \pi \rrbracket , h)$ with ${\textbf{u}}= {\textbf{v}}-{\textbf{v}}_0$, $\pi = p-p_0$, where

$$\begin{aligned} DN(z_0)w&= \begin{pmatrix} {\textbf{v}}_0 \cdot \nabla {\textbf{w}}+{\textbf{w}}\cdot \nabla {\textbf{v}}_0 + (DM_1(0)h+DM_2(0)h+ DM_3(0)h)\nabla {\textbf{v}}_0\\ (DM_1(0) h) : \nabla {\textbf{v}}_0\\ (DG_\tau (0)h)\nabla {\textbf{v}}_0+ ((DG_\nu (0)h)\nabla {\textbf{v}}_0){\textbf{n}}_{\Gamma _t}\\ 0 \end{pmatrix} \end{aligned}$$

since ${\bar{L}}z_0=0$, $M_0(0)=I$, $M_j(0)=0$ for $j=1,\ldots , 4$, $G_\tau (0)=G_\nu (0)=G_\gamma (0)=0$, and $DG_\gamma (0)h= \sigma (DH_{\Gamma _t}(0)- DH_{\Gamma _t}(0))h=0$. Hence $DN(z_0)$ is a linear operator of lower order with respect to *w* compared to $L + m {\bar{L}}$.

As in [27, Proposition 3] we have

#### Proposition 7.4

Let $q>d+2$. Then $N\in C^\omega ({\mathcal {U}},{\mathbb {F}}(T))$ and ${\bar{N}}\in C^\omega ({\mathcal {U}}_m,{\mathbb {F}}(T))$, where

$$\begin{aligned} {\mathcal {U}}:=\{({\textbf{u}}, \pi ,r,h)\in {\mathbb {E}}(T): \Vert h\Vert _{{\mathbb {E}}_4(T)}<r_0\},\quad {\mathcal {U}}_m= {\mathcal {U}}\cap {\mathbb {E}}_m(T) \end{aligned}$$

for $r_0>0$ sufficiently small. Moreover, for every $r_1\in (0,r_0)$ there is some $C>0$ such that

7.41 $$\begin{aligned} \Vert m{\bar{N}}(w_1)- m{\bar{N}}(w_2)\Vert _{{\mathbb {F}}(T)}\le CR \Vert w_1-w_2\Vert _{{\mathbb {E}}_m(T)} \end{aligned}$$

for all $w_1,w_2\in {\mathcal {U}}_m$ with $\Vert w_j\Vert _{{\mathbb {E}}_m(T)}\le R$, $j=1,2$, where $R\in (0,r_1]$.

#### Proof

The statement for *N* is proved in the same way as in [27, Proposition 3], where the only difference is that the reference surface is time-dependent. But because of the compactness of $[0,T_0]$ all relevant norms are uniformly controlled. For the statement on ${\bar{N}}$ we use the quasilinear structure of *K*(*h*), i.e.,

$$\begin{aligned} K(h)= {\mathcal {C}}_0 (h): \nabla ^2_{\Gamma _t} h +{\mathcal {C}}_1(h), \end{aligned}$$

where ${\mathcal {C}}_j(h)$ depend only on *h* and $\nabla _{\Gamma _t}h$ and are analytic in $(h,\nabla _{\Gamma _t}h)$ (pointwise), cf. e.g. [35, Section 2.2.5]. Moreover, due to [33, Lemma 6.1] ${\mathbb {F}}_4(T_0)$ is a Banach algebra and $m\Vert \nabla ^2_{\Gamma _t} h\Vert _{{\mathbb {F}}_4(T_0)}\le C\Vert w\Vert _{{\mathbb {E}}_m(T_0)}$ uniformly in $m\in (0,1]$. From this ${\bar{N}}\in C^\omega ({\mathcal {U}}_m,{\mathbb {F}}(T_0))$ easily follows and one obtains (7.41) in a straight forward manner. $\square$

The central technical results are

#### Proposition 7.5

Let $r_0$ be as in Proposition 7.4. There are some $C_N>0$, $R_0\in (0,r_0)$ such that for every $R\in (0,R_0]$, $m\in (0,1]$ we have

7.42 $$\begin{aligned} \Vert {\mathcal {N}}_m(w_1)-{\mathcal {N}}_m(w_2)\Vert _{{\mathbb {F}}(T_0)}\le C_NR\Vert w_1-w_2\Vert _{{\mathbb {E}}_m(T_0)} \end{aligned}$$

for all $w_1,w_2\in {\mathbb {E}}_m(T_0)$ with $\Vert w_j\Vert _{{\mathbb {E}}_m(T_0)}\le R$ for $j=1,2$.

#### Proof

Let $R_0\in (0,1]$ be at least so small that $R_0< r_0$. Then, by the definition of ${\mathcal {N}}_m$,

$$\begin{aligned} {\mathcal {N}}_m(w_1)-{\mathcal {N}}_m(w_2) =&N(w_1+z_0)-N(w_2+z_0)-DN(z_0)(w_1-w_2)\\&+m\left( {\bar{N}}(w_1+z_0)-{\bar{N}}(w_2+z_0)\right) \end{aligned}$$

for all $w_j\in {\mathcal {U}}$, $j=1,2$. Now using the power series expansion for $N(w_j+z_0)$ and ${\bar{N}}(w_j+z_0)$, we obtain for $R_0$ sufficiently small

$$\begin{aligned} \Vert {\mathcal {N}}_m(w_1)-{\mathcal {N}}_m(w_2)\Vert _{{\mathbb {F}}(T_0)}&\le C\Vert w_1-w_2\Vert _{{\mathbb {E}}(T_0)}^2 + CR\Vert w_1-w_2\Vert _{{\mathbb {E}}_m(T_0)}\\&\le CR \Vert w_1-w_3\Vert _{{\mathbb {E}}_m(T_0)} \end{aligned}$$

for all $w_j\in {\mathcal {U}}_m$ with $\Vert w_j\Vert _{{\mathbb {E}}_m(T_0)}\le R$, $j=1,2$. $\square$

#### Proposition 7.6

Let ${\mathcal {L}}_m$ be defined as in (7.40). Then there are some $m_1\in (0,1]$, $C_L>0$ such that ${\mathcal {L}}_m:{\mathbb {E}}_m(T_0)\rightarrow {\mathbb {F}}(T_0)$ is invertible and

$$\begin{aligned} \Vert {\mathcal {L}}_m^{-1}\Vert _{{\mathcal {L}}({\mathbb {F}}(T_0),{\mathbb {E}}_m(T_0))}\le C_L \qquad \text {for all }m\in (0,m_1]. \end{aligned}$$

#### Proof

First of all $L+m{\bar{L}}:{\mathbb {E}}_m(T)\rightarrow {\mathbb {F}}(T)$ is invertible for all $m\in (0,m_1]$, $m_1\in (0,1]$ sufficiently small, and all $T\in (0,T_0]$ because of Theorem 7.1. Moreover, there is some $C_L'>0$ such that

$$\begin{aligned} \Vert (L+m{\bar{L}})^{-1}\Vert _{{\mathcal {L}}({\mathbb {F}}(T),{\mathbb {E}}_m(T))}\le C_L' \qquad \text {for all }m\in (0,m_1]\text { and }T\in (0,T_0]. \end{aligned}$$

Using

$$\begin{aligned} {\mathbb {E}}_4(T)&\hookrightarrow  _0W^1_q(0,T;W^{2-\frac{1}{q}}_q(\Gamma _t))\cap W^1_r(0,T;W^1_q(\Gamma _t))\\  &\hookrightarrow C^{1-\frac{1}{q}}\left( [0,T];W^{2-\frac{1}{q}}_q(\Gamma _t)\right) \end{aligned}$$

uniformly in $T\in (0,T_0]$ for some $r>q$ and the smoothness of ${\textbf{v}}_0$ one can show

$$\begin{aligned}&\Vert {\textbf{v}}_0 \cdot \nabla {\textbf{w}}+{\textbf{w}}\cdot \nabla {\textbf{v}}_0 + (DM_1(0)h+DM_2(0)h+ DM_3(0)h)\nabla {\textbf{v}}_0\Vert _{L^q(\Omega \times (0,T))}\\&\quad \le CT^{\frac{1}{q}} \Vert h\Vert _{L^\infty (0,T;W^{2-\frac{1}{q}}_q(\Gamma _t))}\le C'T^{\frac{1}{q}}\Vert h\Vert _{{\mathbb {E}}_4(T)} \end{aligned}$$

and

$$\begin{aligned}&\Vert (DM_1(0) h) : \nabla {\textbf{v}}_0\Vert _{{\textbf{F}}_2(T)}\le C \Vert (DM_1(0) h) : \nabla {\textbf{v}}_0\Vert _{W^1_q(\Omega )\times (0,T))}\le CT^{\frac{1}{q}-\frac{1}{r}}\Vert h\Vert _{{\mathbb {E}}_4(T)},\\&\Vert (DG_\tau (0)h)\nabla {\textbf{v}}_0+ ((DG_\nu (0)h)\nabla {\textbf{v}}_0){\textbf{n}}_{\Gamma _t}\Vert _{{\mathbb {F}}_3(T)}\le CT^\alpha \Vert h\Vert _{{\mathbb {E}}_4(T)} \end{aligned}$$

uniformly in $T\in (0,T_0]$ and $h\in {\mathbb {E}}_4(T)$ for some $\alpha >0$ by straightforward estimates. Hence

$$\begin{aligned} \Vert DN(z_0)(L+m{\bar{L}})^{-1}\Vert _{{\mathcal {L}}({\mathbb {F}}(T))}\le CT^{\alpha } \qquad \text {for all }m\in (0,m_1]\text { and }T\in (0,T_0] \end{aligned}$$

for some $\alpha >0$. Hence by a Neumann series argument ${\mathcal {L}}_m$ is invertible and

$$\begin{aligned} \Vert {\mathcal {L}}_m^{-1}\Vert _{{\mathcal {L}}({\mathbb {F}}(T),{\mathbb {E}}_m(T))}\le C_L \qquad \text {for all }m\in (0,m_1] \end{aligned}$$

provided that $T\in (0,T_*]$ for some $T_*>0$ sufficiently small. Finally, the invertibility of ${\mathcal {L}}_m$ and the uniform estimate on the time interval $(0,T_0)$ can be shown by the same arguments as in the end of the proof of Theorem 7.1 by dividing $(0,T_0)$ in finitely many intervals $(0,T_1)$, $(T_1,T_2), \ldots ,(T_{N-1},T_N)$ of length less than $T_*$ and solving the system iteratively on the intervals. $\square$

Now let

$$\begin{aligned} R_m:=2m C_L\Vert {\bar{N}}(z_0)\Vert _{{\mathbb {F}}(T)} \end{aligned}$$

and choose $m_0\in (0,m_1]$ so small that

$$\begin{aligned} C_L C_N (R_{m_0} +m_0)\le \frac{1}{2}\quad \text {and}\quad R_{m_0}\le R_0. \end{aligned}$$

Then we have for every $m\in (0,m_0]$

$$\begin{aligned} \Vert {\mathcal {L}}_m^{-1}\left( {\mathcal {N}}_m(w_1)-{\mathcal {N}}_m(w_2)\right) \Vert _{{\mathbb {E}}_m(T)}&\le C_LC_N(R_m+m)\Vert w_1-w_2\Vert _{{\mathbb {E}}_m(T)}\\&\le \frac{1}{2}\Vert w_1-w_2\Vert _{{\mathbb {E}}_m(T)} \end{aligned}$$

for all $w_1,w_2\in {\mathbb {E}}_m(T)$ with $\Vert w_j\Vert _{{\mathbb {E}}_m(T)}\le R_m$ for $j=1,2$ and

$$\begin{aligned} \Vert {\mathcal {L}}_m^{-1}{\mathcal {N}}_m(w)\Vert _{{\mathbb {E}}_m(T)}&\le \Vert {\mathcal {L}}_m^{-1}\left( {\mathcal {N}}_m(w)-{\mathcal {N}}_m(0)\right) \Vert _{{\mathbb {E}}_m(T)}+ C_L\Vert {\mathcal {N}}_m(0)\Vert _{{\mathbb {E}}_m(T)}\\&\le \frac{R_m}{2}+ m C_L\Vert {\bar{N}}(z_0)\Vert _{{\mathbb {F}}(T)}\le R_m \end{aligned}$$

for all $w\in {\mathbb {E}}_m(T)$ with $\Vert w\Vert _{{\mathbb {E}}_m(T)}\le R_m$. Hence ${\mathcal {L}}_m^{-1}{\mathcal {N}}_m$ is a contraction on $\overline{B_{R_m}(0)}$ in ${\mathbb {E}}_m(T)$ for all $m\in (0,m_0]$ and we obtain for every $m\in (0,m_0]$ a unique $w_m\in \overline{B_{R_m}(0)}$ such that

7.43 $$\begin{aligned} w_m = {\mathcal {L}}_m^{-1}({\mathcal {N}}_m w_m). \end{aligned}$$

In summary, we obtain

#### Theorem 7.7

There is some $m_0>0$ such that for every $m\in (0,m_0]$ the transformed system (7.32)–(7.38) possesses a solution $({\textbf{v}},p,\llbracket p\rrbracket ,h)\in {\mathbb {E}}_m(T_0)$, which satisfies

$$\begin{aligned} \Vert ({\textbf{v}}-{\textbf{v}}_0,p-p_0,\llbracket p - p_0\rrbracket ,h)\Vert _{{\mathbb {E}}_m(T_0)}\le Cm \end{aligned}$$

for some $C>0$ independent of $m\in (0,m_0]$.

Transforming back with $\Theta _h^{-1}$ finally yields a solution $({\textbf{v}}_m^\pm ,p_m^\pm , (\Gamma ^m_t)_{t\in [0,T_0]})$ of (7.1)–(7.7).

#### Remark 7.8

Since ${\mathbb {E}}_{4,m}(T_0)\hookrightarrow C^0([0,T_0];C^2(\Gamma _t))$ uniformly in $m\in (0,1)$, one can show in a straight forward manner that there are $m_1\in (0,m_0]$ and $\delta >0$ such that the signed distance function $d_{\Gamma ^m_t}$ and the orthogonal projection $P_{\Gamma ^m_t}$ on $\Gamma _t$ for every $t\in [0,T_0]$ are well-defined and smooth in $\Gamma ^m(2\delta )= \{(x,t)\in \Omega \times [0,T_0]: |{\text {dist}}(x,\Gamma ^m_t)|<2\delta \}$ for every $m\in (0,m_1]$. Moreover,

$$\begin{aligned} \Gamma ^m(2\delta )\subseteq \Gamma (3\delta ) \quad \text {for all }m\in (0,m_1] \end{aligned}$$

and $\delta >0$ can be chosen (as before) such that the signed distance function $d_{\Gamma _t}$ and the orthogonal projection $P_{\Gamma _t}$ on $\Gamma _t$ for every $t\in [0,T_0]$ are smooth in $\Gamma (3\delta )$.

### Uniform Regularity

The goal of this section is to prove

#### Theorem 7.9

Let $q>d+5$. There is some $m_0>0$ such that for every $m\in (0,m_0]$ (7.1)–(7.6) possesses a solution $({\textbf{v}}_m^\pm , p_m^\pm , \Gamma ^m)$ with $\Gamma ^m\subseteq \Gamma (\delta )$, $d_{\Gamma ^m_t}\in C^1_t C^1_x\cap C^0_tC^3_x$ in $\overline{\Gamma (\delta )}$ and ${\textbf{v}}_m\in L^{q}(0,T;W^1_{q}(\Omega )^d)\cap L^{q}(0,T;W^2_{q}(\Omega \setminus \Gamma _t^m))\cap W^1_{q}(0,T;L^{q}_\sigma (\Omega ))$ and $\nabla _\tau \nabla {\textbf{v}}_m\in L^\infty (\Omega \times (0,T))$, $\nabla _\tau p_m\in L^{q}(0,T;W^1_{q}(\Omega \setminus \Gamma _t))$ with uniformly bounded norms with respect to $m\in (0,m_0]$.

#### Proof

We use the so-called parameter-trick to obtain that the tangential derivative of the solution belongs locally to the same function space as the solution itself (together with uniform bounds). To this end we follow the arguments in [35, Section 9.4.2] with modifications to our time dependent reference manifold $\Gamma _t$. Let $(t_0,x_0)\in \Gamma$ be arbitrary and ${\tilde{X}}:{\overline{\Omega }}\times J_{t_0} \rightarrow {\overline{\Omega }}$ with ${\tilde{X}}(x,t)= X_t(X^{-1}_{t_0}(x))$ for all $x\in \Omega$, $t\in J_{t_0}:= [\max (t_0-\delta ,0), \min (T_0,t_0+\delta )]$ as in Section 7.3. Moreover, let $\varphi :B_{3R}(0)\subseteq {\mathbb {R}}^{d-1}\rightarrow {\mathbb {R}}^d$ be a local parametrization of $\Gamma _{t_0}$ with $\varphi (0)= x_0$. Furthermore, for $t\in J_{t_0}$ let $\phi _t :B_{3R}(0) \times (-2\delta _0, 2\delta _0)\rightarrow {\mathbb {R}}^d$ be defined by

$$\begin{aligned} \phi _t(s,\rho )= &   {\tilde{X}}(\varphi (s),t)+ \rho {\textbf{n}}_{\Gamma _t}\\    &   \quad \text {for all }(s,\rho )\in B_{3R}(0)\times (-2\delta _0, 2\delta _0). \end{aligned}$$

Then $P_{\Gamma _t}(\phi _t(s,\rho ))= {\tilde{X}}(\varphi (s),t)$. We define the truncated shift $\tau _\xi :J_{t_0}\times B_{3R}(0)\times (-2\delta _0, 2\delta _0)\rightarrow B_{3R}(0)\times (-2\delta _0, 2\delta _0)$

$$\begin{aligned} \tau _\xi (t,s,\rho )= &   (s+\xi \eta (t)\chi _0(s)\zeta _0(\rho ),\rho )\quad \text {for all }(t,s,\rho )\in J_{t_0}\\  &   \times B_{3R}(0)\times (-2\delta _0, 2\delta _0), \end{aligned}$$

where $\xi \in B_r(0)\subseteq {\mathbb {R}}^{d-1}$, $0<r\le R$, $\chi _0\in C^\infty _0({\mathbb {R}}^{d-1})$ with $0\le \chi _0\le 1$, ${\text {supp}}\chi _0\subseteq B_{2R}(0)$ and $\chi _0(s)=1$ if $|s|\le R$, $\zeta _0\in C_0^\infty ({\mathbb {R}})$ with ${\text {supp}}\zeta _0 \subseteq [-5\delta /2, 5\delta /2]$ and $\zeta _0(\rho )=1$ if $|\rho |\le 2\delta _0$, $\eta \in C_0^\infty ({\mathbb {R}})$ with $\eta (t)=0$ if $t\in [t_0-\delta /2,t_0+\delta /2]$ and ${\text {supp}}\eta \subseteq (t_0-\delta , t_0+\delta )$. Finally, we define

$$\begin{aligned}  &   \varvec{\tau }_\xi (t,x)= \phi _t(\tau _\xi (t,\phi _t^{-1}(x)))\\  &   \quad \text {for all } (t,x)\in U:=\bigcup _{t\in J_{t_0}}\{t\}\times U_t,\ U_t:= \phi _t\left( B_{3R}(0)\times (-\tfrac{3\delta _0}{2}, \tfrac{3\delta _0}{2})\right) . \end{aligned}$$

and $\varvec{\tau }_\xi (t,x)= (t,x)$ for all $t\in J_{t_0}$, $x\in \Omega \setminus U_t$ and $t\in [0,T]\setminus J_{t_0}$, $x\in \Omega$. Note that $P_{\Gamma _t}(\varvec{\tau }_\xi (t,x))= \tau _\xi (t,P_{\Gamma _t}(x))$ for all $x\in U_t$, $t\in J_{t_0}$ and therefore

$$\begin{aligned} h\circ ({\text {id}},P_{\Gamma _\cdot })\circ \varvec{\tau }_\xi (t,x)= h(t, P_{\Gamma _t} (\varvec{\tau }_{\xi }(t,x)))= (h(t,\cdot )\circ \tau _\xi )\circ ({\text {id}},P_{\Gamma _\cdot })(t,x) \end{aligned}$$

for all $(t,x)\in U$ and thus

$$\begin{aligned} \Theta _h(\varvec{\tau }_\xi (t,x),t)= \Theta _{h\circ ({\text {id}}, \tau _\xi )} (x,t)\quad \text {for all }(t,x)\in U \end{aligned}$$

since $d_{\Gamma _t}\circ \varvec{\tau }_\xi (t,\cdot )= d_{\Gamma _t}$ on $U_t$ and $\chi \circ (d_{\Gamma _t}/\delta )=0$ on $\Gamma _t(2\delta )\setminus U_t$ for all $t\in J_{t_0}$.

Altogether we observe that for $r>0$ sufficiently small $\varvec{\tau }_\xi (t,\cdot ):{\overline{\Omega }}\rightarrow {\overline{\Omega }}$ is a smooth diffeomorphism, which depends smoothly on $\xi \in B_r(0)$ and $t\in [0,T]$. We denote by $T_\xi :{\mathbb {E}}_m(T)\rightarrow {\mathbb {E}}_m(T)$ the operator obtained by pointwise composition with $\varvec{\tau }_\xi$. Let $G_m:\overline{B_R(0)}\subseteq {\mathbb {E}}_m(T)\rightarrow \overline{B_R(0)}$ with $G_m(w):=w-{\mathcal {L}}_m^{-1}({\mathcal {N}}_m w)$ and $w_m$ be as in (7.43). Then

$$\begin{aligned} 0= T_\xi G_m (w_m)= T_\xi G_m(T_\xi ^{-1}T_\xi w_m)\qquad \text {for all }\xi \in B_r(0). \end{aligned}$$

Therefore we define ${\mathcal {G}}_m:B_r(0)\times B_R(0)\subseteq {\mathbb {R}}^{d-1}\times {\mathbb {E}}_m(T)\rightarrow {\mathbb {E}}_m$ by

$$\begin{aligned} {\mathcal {G}}_m(\xi , w):= T_\xi G_m(T_\xi ^{-1} w). \end{aligned}$$

Then as in [35, Section 9.4.2] one observes that ${\mathcal {G}}_m$ is continuously differentiable and $\partial _{\xi _j} {\mathcal {G}}_m (0,w_m)\in {\mathbb {E}}_m(T)$ is bounded with respect to $m\in (0,1]$. Furthermore $w_{m,\xi }:= T_\xi w_m$ solves

$$\begin{aligned} {\mathcal {G}}_m(\xi , w_{m,\xi })=0\qquad \text {for all }\xi \in B_r(\xi ). \end{aligned}$$

In particular, ${\mathcal {G}}_m(0, w_m)=0$ and

$$\begin{aligned} D_w{\mathcal {G}}_m(0, w_m) = DG_m(w_m)=I-{\mathcal {L}}_m^{-1}D{\mathcal {N}}_m(w_m):{\mathbb {E}}_m(T)\rightarrow {\mathbb {E}}_m(T) \end{aligned}$$

is invertible since $\Vert {\mathcal {L}}_m^{-1}D{\mathcal {N}}_m(w_m)\Vert _{{\mathcal {L}}({\mathbb {E}}_m(T))}\le \frac{1}{2}$ due to Proposition 7.5 for $m\in (0,m_1]$ with $m_1>0$ sufficiently small as before. Furthermore, $\Vert DG_m(w_m)^{-1}\Vert _{{\mathcal {L}}({\mathbb {E}}_m(T))} \le 2$ uniformly in $m\in (0,m_1]$. Now the Implicit Function Theorem yields that, if $r>0$ is sufficiently small, there are some $r'>0$ and continuously differentiable $\Phi _m:B_r(0)\rightarrow B_{r'}(w_m)\subseteq {\mathbb {E}}_m(T)$ such that $B_{r'}(w_m)\subseteq B_R(0)$ and

1. $${\mathcal {G}}_m(\xi , \Phi _m(\xi ))=0$$ for all $$\xi \in B_r(0)$$.
2. If ${\mathcal {G}}_m(\xi , u)=0$ for some $u\in B_R(0)\subseteq {\mathbb {E}}_m(T)$ and $\xi \in B_r(0)$, then $u= \Phi _m(\xi )$.

Hence $\Phi _m(\xi )= w_{m,\xi }$ for all $\xi \in B_r(0)$. To obtain uniform boundedness of $\partial _{\xi _j} w_{m,\xi }|_{\xi =0}$ we use that

$$\begin{aligned} \partial _{\xi _j} w_{m,\xi }|_{\xi =0} = - D_w {\mathcal {G}}_m(0,w_m)^{-1} \partial _{\xi _j} {\mathcal {G}}_m(0, w_m)= - D G_m(w_m)^{-1} \partial _{\xi _j} {\mathcal {G}}_m(0, w_m), \end{aligned}$$

where $\partial _{\xi _j} {\mathcal {G}}_m(0, w_m)\in {\mathbb {E}}_m(T)$ and $D_w G_m(w_m)^{-1}$ are uniformly bounded in $m\in (0,m_1]$ by the same observations before. Since $\partial _{\xi _j} u\circ \tau _\xi |_{\xi =0} = \partial _{\varvec{\tau }_j} u$ in a neighborhood of $x_0$, where $\varvec{\tau }_j(x)=\partial _{p_j} {\tilde{X}}(\varphi (p),t)$, $j=1,\ldots ,d-1$, (with $x=\phi _t(p,q)$) form a basis of $T_x\Gamma _t$, the statement of the theorem follows. $\square$

## Data Availability

There is no associated data to the manuscript.

## Footnotes

1 The only previous work in this direction [4] only explicitly covers the case of the scaling regime $m_\varepsilon =\varepsilon ^{1/2}$ and works under substantially stronger assumptions on the initial data.
